# Glycoproteomic and Phenotypic Elucidation of *B4GALNT2* Expression Variants in the SID Histo-Blood Group System

**DOI:** 10.3390/ijms23073936

**Published:** 2022-04-01

**Authors:** Linn Stenfelt, Jonas Nilsson, Åsa Hellberg, Yew Wah Liew, Jenny Morrison, Göran Larson, Martin L. Olsson

**Affiliations:** 1 Division of Hematology and Transfusion Medicine, Department of Laboratory Medicine, Lund University, SE 221 85 Lund, Sweden; linn.stenfelt@med.lu.se; 2 Proteomics Core Facility, Sahlgrenska Academy at the University of Gothenburg, SE 405 30 Gothenburg, Sweden; 3 Department of Clinical Immunology and Transfusion Medicine, Office for Medical Services, Region Skåne, SE 221 85 Lund, Sweden; asa.hellberg@med.lu.se; 4 Red Cell Reference Laboratory, Clinical Services and Research, Australian Red Cross Lifeblood, Kelvin Grove, Brisbane, QLD 4059, Australia; yliew@redcrossblood.org.au (Y.W.L.); jmorrison@redcrossblood.org.au (J.M.); 5 Laboratory of Clinical Chemistry, Sahlgrenska University Hospital, SE 413 45 Gothenburg, Sweden; goran.larson@clinchem.gu.se; 6 Department of Laboratory Medicine, Institute of Biomedicine, Sahlgrenska Academy at the University of Gothenburg, SE 413 45 Gothenburg, Sweden

**Keywords:** SID blood group system, carbohydrate biosynthesis, erythrocyte, flow cytometry, gene expression, glycobiology, glycoprotein, glycopeptide, glycosyltransferase, mass spectrometry (MS)

## Abstract

The Sd^a^ histo-blood group antigen (GalNAcβ1-4(NeuAcα2-3)Galβ-R) is implicated in various infections and constitutes a potential biomarker for colon cancer. Sd(a−) individuals (2–4% of Europeans) may produce anti-Sd^a^, which can lead to incompatible blood transfusions, especially if donors with the high-expressing Sd(a++)/Cad phenotype are involved. We previously reported the association of *B4GALNT2* mutations with Sd(a−), which established the SID blood-group system. The present study provides causal proof underpinning this correlation. Sd(a−) HEK293 cells were transfected with different *B4GALNT2* constructs and evaluated by immunostaining and glycoproteomics. The predominant *SID^null^* candidate allele with rs7224888:T>C (p.Cys406Arg) abolished Sd^a^ synthesis, while this antigen was detectable as N- or O-glycans on glycoproteins following transfection of wildtype *B4GALNT2*. Surprisingly, two rare missense variants, rs148441237:A>G and rs61743617:C>T, found in a Sd(a−) compound heterozygote, gave results similar to wildtype. To elucidate on whether Sd(a++)/Cad also depends on *B4GALNT2* alterations, this gene was sequenced in five individuals. No Cad-specific changes were identified, but a detailed erythroid Cad glycoprotein profile was obtained, especially for glycophorin-A (GLPA) O-glycosylation, equilibrative nucleoside transporter 1 (S29A1) O-glycosylation, and band 3 anion transport protein (B3AT) N-glycosylation. In conclusion, the p.Cys406Arg β4GalNAc-T2 variant causes Sd^a^-deficiency in humans, while the enigmatic Cad phenotype remains unresolved, albeit further characterized.

## 1. Introduction

The SID histo-blood group system was acknowledged by the International Society of Transfusion Medicine (ISBT) when an association to the *B4GALNT2* gene was shown [1,2]. There is only one antigen in the system, namely the Sd^a^ antigen, which had already been discovered in 1967 [3,4]. Approximately 91% of the European population carry the antigen on their erythrocytes, although 96–98% express it in tissues such as colon and kidney, or soluble in urine and saliva [5,6]. Among pregnant women, the incidence of Sd(a+) erythrocytes is lower than in normal blood donors [3,6,7], which is similar to the Lewis system, where glycolipid antigens are passively adsorbed from plasma by the erythrocytes [8]. No Sd^a^ is detected on the erythrocytes of newborns, while high levels are detected in their saliva [6]. The 2–4% of adults who are truly Sd^a^ negative may produce naturally-occurring antibodies against the antigen. Although rare, hemolytic transfusion reactions have occurred [9,10], and rising IgG titers have been seen in patients after incompatible transfusions [7]. Especially, transfusion of erythrocytes from donors with particularly high Sd^a^ expression should therefore be avoided in patients with the Sd(a−) phenotype and antibodies to Sd^a^, since this could lead to hemolytic transfusion reactions. The rare Sd(a++) phenotype is defined by erythrocytes being highly reactive with anti-Sd^a^. The most striking example of such a high-expressing phenotype is Cad, first detected in 1968 when erythrocytes of blood group B and O were strongly agglutinated by the *Dolichos biflorus* agglutinin (DBA). This is a plant lectin otherwise used to type for the blood group A antigen, as it binds to terminal GalNAc residues [11]. The structure of the anti-Sd^a^ binding epitope was defined in erythrocytes from Cad individuals as GalNAcβ1-4(NeuAcα2-3)Gal-R, which is found on O-linked core 1 structures carried on glycophorin A (GLPA) and on glycolipids in the shape of elongated sialyl-paragloboside [12,13]. In the urine of Sd(a+) individuals, Sd^a^ is carried on N-linked glycans attached to the Tamm–Horsfall glycoprotein (uromodulin) and on core 3 *O*-GalNAc glycans on mucins in colon [14,15]. However, Sd^a^ epitopes on N-glycans have not, to our knowledge, been described on other major glycoproteins on erythrocytes, such as, for instance, the band 3 anion transport protein (B3AT) [16]. The antigen has been suggested to be used as a colon tumor marker due to its downregulation in cancerous tissue in favor of the sialyl-Lewis X (sLe^x^) epitope, the synthesis of which utilizes the same sialylated precursors as those of the Sd^a^ epitope [17]. Additionally, the structure seems to inhibit various pathogens from adhering or invading human cells [18,19,20].

The *B4GALNT2* gene was cloned in 2003 and found to encode a *β*-1,4-*N*-acetylgalactosaminyltransferase (β4GalNAc-T2) able to synthesize the Sd^a^-specific glycan epitope [21,22]. Two transcripts were detected, differing only in the use of a longer or a shorter alternative exon 1 and thereby generating enzymes with different lengths of the cytoplasmic tail [22]. The gene seems not to be expressed in the erythroid lineage [23] and, despite the revealed identity of the implicated glycosyltransferase, genetic differences between the three main phenotypes, Sd(a−), Sd(a+), and Sd(a++)/Cad, have remained unresolved for decades. In 2019, the nonsynonymous single nucleotide polymorphism (SNP) rs7224888:T>C (GenBank accession no. MK765047) in *B4GALNT2* was found to dominate two different cohorts of nine Sd(a−) individuals each, making up 13 of 18 and 17 of 18 alleles, respectively [1,2]. Even if these data were mainly correlative and descriptive, they led to the decision by the International Society of Blood Transfusion (ISBT) Working Party on Red Cell Immunogenetics and Blood Group Terminology to promote the high-frequency antigen Sd^a^ from the 901 series of blood group antigens to form an independent, new blood group system of its own, SID (ISBT no. 038, established on June 22, 2019). These studies also found one allele each with an intron 8 splice-site polymorphism, rs72835417:G>A (MK797056). In addition, our study found one Sd(a−) individual compound heterozygous for two rare missense mutations, rs148441237:A>G (MK765048) and rs61743617:C>T (MK765049), and one individual in whom no variation was found in the *B4GALNT2* regions investigated [1].

In the present study, we aimed to provide conclusive proof that these missense variants cause Sd^a^ deficiency in humans. In addition, we added glycopeptide LC-MS/MS analyses to expand our knowledge on Sd^a^-carrying glycoproteins and, more specifically, describe the epitope in its different glycoforms. Furthermore, we hypothesized and searched for a possible correlation between *B4GALNT2* sequences, the Sd^a^ antigen, and its erythrocyte glycoprotein carriers in individuals with the Cad phenotype.

## 2. Results

### 2.1. Transfection of B4GALNT2 Constructs in HEK293 Cells

**Figure 1:**
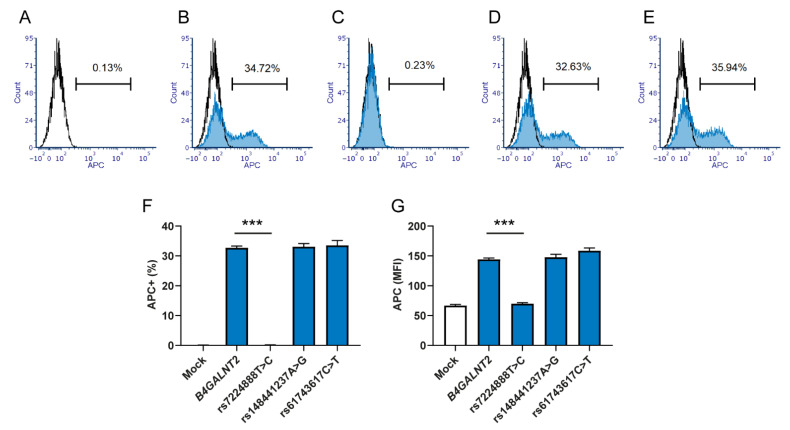
Lectin analysis by flow cytometry of terminal GalNAc residues on HEK293 cells transfected with *B4GALNT2* constructs. HEK293 cells transfected with the bicistronic GFP-expressing vector (CS-2719-M61) carrying *B4GALNT2* constructs were stained with biotinylated DBA lectin followed by APC-conjugated streptavidin. Flow cytometry was used to record the level of GalNAc expression in GFP-positive cells. Cells transfected with the vector carrying (**A**) no construct (Mock) (**B**) *B4GALNT2* wt (GenBank accession number AJ517771), (**C**) *B4GALNT2* with Sd(a−)-associated SNP rs7224888:T > C, (**D**) rs148441237:A > G, or (**E**) rs61743617:C > T. Graphs present data from three separate experiments giving (**F**) the percentage of cells positive for the APC signal and (**G**) the mean MFI of the APC signal, with error bars that represent the standard error of the mean (SEM) and asterisks (***) indicate *p* < 0.001.

**Figure 2:**
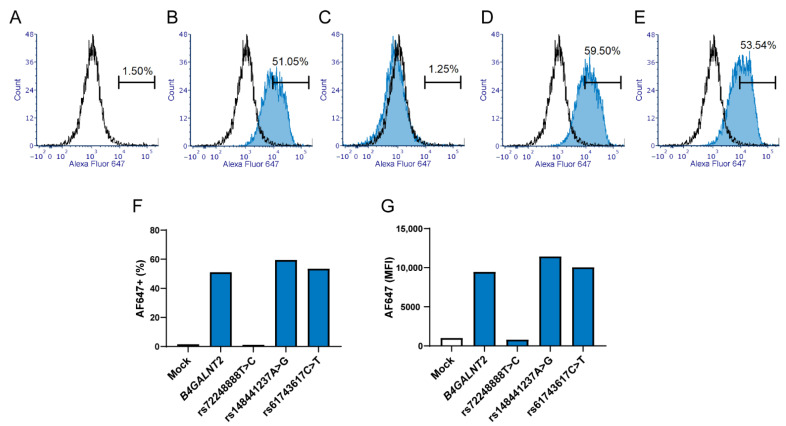
Antibody analysis of Sd^a^ surface expression in HEK293 cells transfected with *B4GALNT2* constructs. One set of transfected cells were stained with human plasma containing anti-Sd^a^ (diluted in PBS) followed by staining with Alexa Fluor 647 conjugated Goat anti-human:IgM. The GFP positive population in HEK293 cells transfected with the vector CS-2719-M61 carrying (**A**) no construct (mock), (**B**) *B4GALNT2* wt (GenBank accession number AJ517771), (**C**) *B4GALNT2* with Sd(a−) associated SNPs rs7224888:T > C, (**D**) rs148441237:A > G or (**E**) rs61743617:C > T. Graphs present (**F**) the percentage of cells positive for the Alexa Fluor 647 signal and (**G**) the MFI of the Alexa Fluor 647 signal. Due to the small amounts of human anti-Sd^a^ available, only one set of transfected samples was run, not allowing for statistical evaluation.

The HEK293 cell line was chosen as a model because of its lack of Sd^a^ epitopes (Figure 1A) and low *B4GALNT2* mRNA expression (data not shown). Cells mock-transfected with the vector pReceiver-M61C1 (GFP positive population) were negative controls for the GalNAc staining, using biotinylated DBA and allophycocyanin (APC)-conjugated streptavidin (Figure 1A). The construct with the consensus sequence of *B4GALNT2* generated DBA binding to a portion of the cells, seen as a bimodal curve in the histogram (Figure 1B), while introducing rs7224888:T>C in the construct showed only background binding with DBA (Figure 1C). The two rare SNPs, rs148441237:A>G and rs61743617:C>T, appeared to cause no alteration in staining compared to consensus (Figure 1D,E). The reduced level of the DBA binding signal in the *B4GALNT2*_rs7224888 was significant (*p* < 0.001), both when comparing the percentage of APC-positive populations (Figure 1F) and the mean MFIs (Figure 1G). Staining controls, in which cells were incubated with streptavidin only, showed equal APC levels to the mock-transfected levels for all cells (data not shown). In one experiment, one set of the triplicates was also subjected to staining with anti-Sd^a^ from human plasma. The antibody reacted with the same transfectants as the DBA (Figure 2A–G). However, unlike the DBA, the whole population of cells, except for the rs7224888:T>C construct which showed only background reactivity, shifted in the histograms, suggesting that all the transfected cells in the samples carried the epitope. The secondary antibody alone was used as a background staining control and so was performing the staining protocol with ABO-matched human plasma lacking anti-Sd^a^.

**Figure 3:**
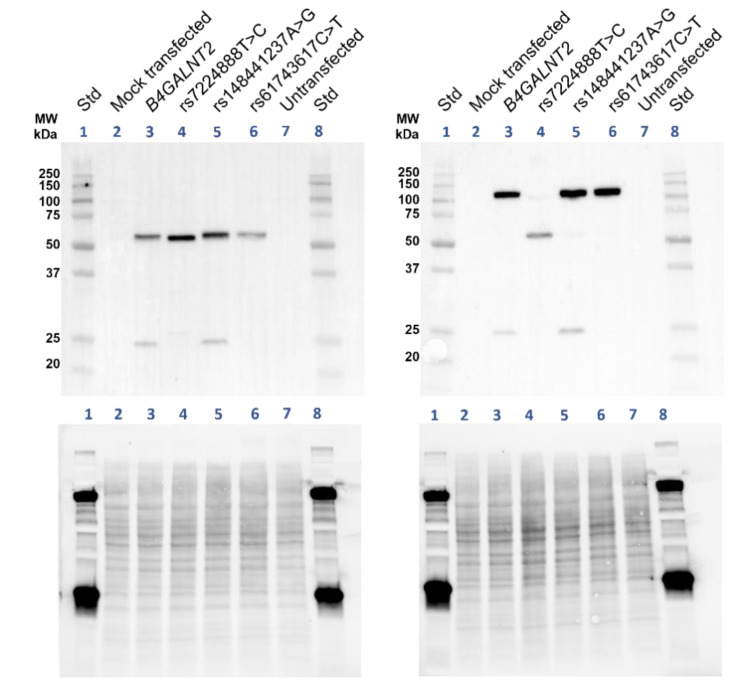
Immunoblot of the *B4GALNT2*-expressed glycosyltransferase in transfected HEK293 cells. Western blot displaying the immunoblotted band corresponding to β4GalNAc-T2 in protein samples from transfected HEK293 cells. In the left blot, samples are dissolved in Laemmli buffer with reducing agent β-mercaptoethanol, while Laemmli buffer without reducing agent has been used for samples of the right blot. The standards (Lane 1 and 8) specify the molecular weights (kDa). Mock-transfected and untransfected cells are negative controls (Lane 2 and 7) with the *B4GALNT2* wt transfected serves as the positive control (Lane 3). The glycosyltransferase is detected in all cell preparations transfected with either of the *B4GALNT2*-mutated constructs, rs7224888:T > C (Lane 4), rs148441237:A > G (Lane 5) or rs61743617:C > T (Lane 6). Colorimetric and chemiluminescent images of the membrane have been merged. The lower panel displays stain-free images of total protein of each blot, ChemiDoc imaging system, Bio-Rad.

To ensure that the *B4GALNT2* constructs were readily expressed as glycosyltransferase in the cells, the β4GalNAc-T2 in the cell preparations was immunoblotted. The transferase (expected size 57 kDa) was present in similar amounts in all samples transfected with a construct carrying the gene, including all three mutants evaluated (Figure 3). Under reducing conditions, all four expression variants migrated as monomers at approximately 57 kDa, although a small shift in size was observed for the rs7224888:T>C mutant. In the absence of a reducing agent, immunoblotting resulted in bands of twice the expected size in the wildtype (wt) and two of the mutants, indicating the formation of homodimers as previously reported for the homologue β4GalNAc-T1 [24,25]. In contrast, the larger band was almost abolished in favor of the 57-kD monomer band encoded by the rs7224888:T>C mutant, in which p.406Cys is exchanged for Arg.

Finally, an additional experiment was performed, in which co-transfection of constructs carrying either of the two rare Sd(a–)-associated alleles with rs148441237:G>A and rs62743617:C>T were introduced simultaneously. However, no alteration of the DBA staining compared to control cells transfected with wt *B4GALNT2* was noted in flow cytometry (data not shown).

### 2.2. Glycoproteomic Analysis of Transfected Cells

**Figure 4:**
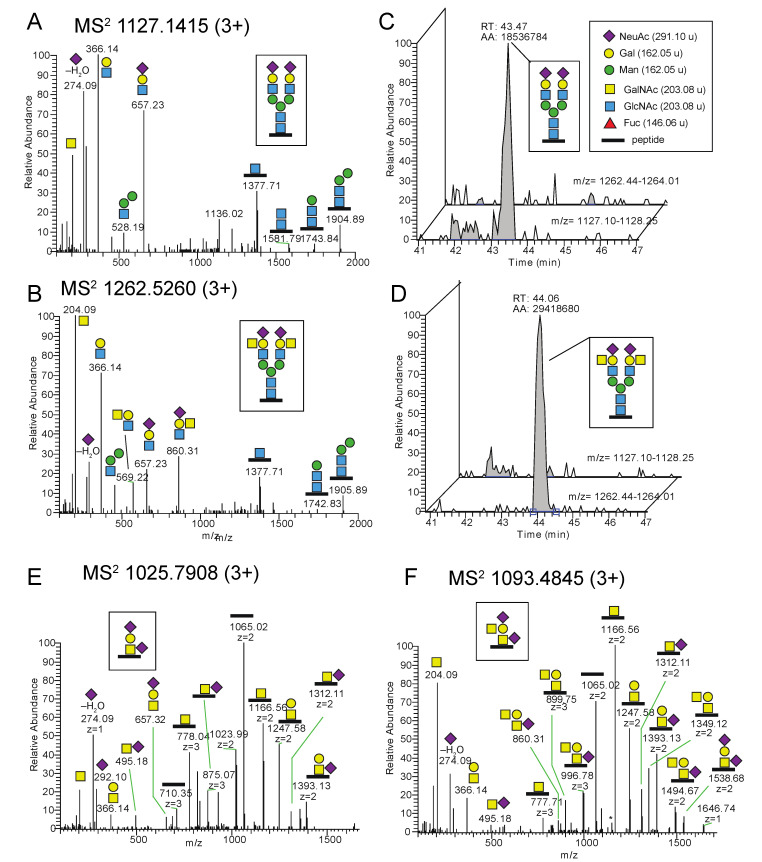
Glycoproteomic analysis of *N*- and O-glycopeptides carrying the Sd^a^ epitope or its precursor structure in transfected HEK293 cells. MS^2^ spectra obtained at NCE 20% for (**A**) a complex type disialo biantennary N-glycopeptide with the amino acid sequence IVDVNLTSEGK including the Asn-174 glycosite (underlined), from transmembrane 9 superfamily member 3 (TM9S3) and for (**B**) a glycopeptide with the same amino acid sequence carrying one Sd^a^ epitope on each of the two antennae. The measured monoisotopic masses for precursor ions are provided in the panel headings (**C**,**D**) Extracted ion chromatograms (XICs) of the two precursor ions demonstrate that (**C**) the mock-transfected sample contains the complex biantennary glycopeptide but not the Sd^a^ epitope glycopeptide, and (**D**) vice versa is true for the wt *B4GALNT2-*transfected sample. (**E**) MS^2^ spectrum obtained at NCE 20% of a glycopeptide, with the amino acid sequence LAGTESPVREEPGEDFPAAR from transferrin receptor protein 1 (TFR1), carrying the disialo core 1 O-glycan, and (**F**) a glycopeptide with the same sequence carrying the Sd^a^ epitope. The glycosite is at Thr-104 or Ser-106. It should be observed that the measured monoisotopic masses for precursor ions at four decimals are provided in the headings of the MS^2^ spectra. Displayed *m*/*z* values of fragment ions are from the largest isotope peaks, not always from the monoisotopic ion. Thus, delta masses in the figures occasionally differ by ±1 u from calculated values.

**Figure 5:**
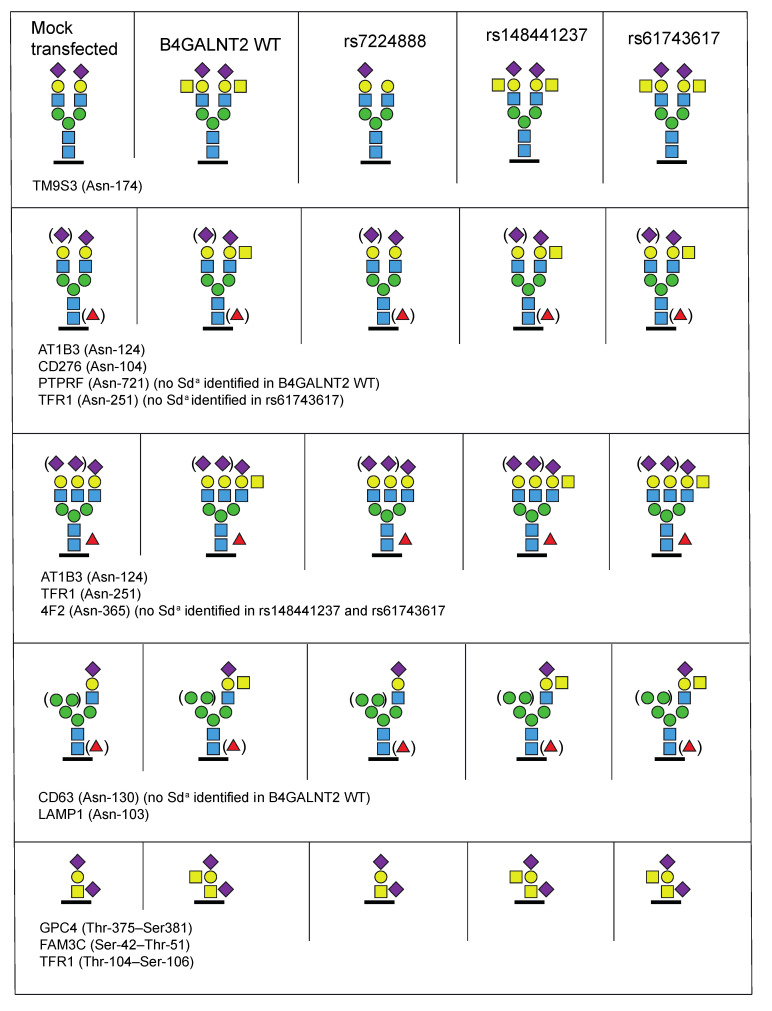
Summary of identified sialic acid containing N- and O-glycopeptides from the transfected HEK293 cell preparations. The transfected HEK293 cells are organized in columns and the identified glycoproteins with their corresponding glycan isoforms in rows. UniProt abbreviations are used and unambiguous Asn- and candidate Ser/Thr-glycosites are indicated by amino acid numbers. The protein names and glycopeptide identities are provided in Table 1.

To further define the causality and differences observed between wt and mutated constructs, and to look for structural glycoproteomic differences between transfectants, LC-MS/MS was applied to characterize the Sd^a^ epitope and the carrier glycoproteins found in the cells transfected with the different *B4GALNT2* constructs. The trypsin-digested samples from the HEK293 preparations were subjected to hydrophilic interaction liquid chromatography (HILIC) to enrich glycopeptides. For the LC-MS/MS analysis, the precursor ions were subjected to higher-energy collision dissociation (HCD) at a relatively low normalized collision energy (NCE) of 20% to enable glycosidic fragmentation of the glycan part while leaving the peptide part intact. Further fragmentation of the same precursor ions at NCEs of 30% and 40% were then carried out to yield peptide backbone fragmentations into characteristic b- and y-ions to identify the peptide sequences and, thus, the carrier glycoproteins and, where possible, the glycan attachment sites using the Byonic software. For instance, a glycopeptide identity from transmembrane 9 superfamily member 3 (TM9S3) carrying a NeuAc_2_Hex_5_HexNAc_4_ structure, in line with a complex type disialo biantennary N-glycan at *m/z* 1127.14, was identified from the mock-transfected sample (Figure 4A). However, the same glycopeptide was not identified in the sample of wt *B4GALNT2* transfected cells. Instead, an alternative glycopeptide from TM9S3 carrying a NeuAc_2_Hex_5_HexNAc_6_ structure was identified after close inspection of these spectra, at *m/z* 1262.53 (Figure 4B), which had the same peptide + HexNAc ion at *m*/*z* 1377.71. At the 20% collision energy level, the NeuAc_2_Hex_5_HexNAc_6_ glycopeptide fragmented into an oxonium ion at *m*/*z* 860.31, having a NeuAcHexHexNAc_2_ composition, convincingly demonstrating the presence of the typical Sd^a^ glycan epitope. This Sd^a^ epitope glycopeptide (at *m*/*z* 1262.53) was not detected in the extracted ion chromatogram (XIC) of the mock-transfected cells where instead the unmodified glycopeptide at *m*/*z m*/*z* 1127.14 was dominating (Figure 4C). In the corresponding XICs, the glycopeptide with an unmodified disialo biantennary structure was thus not detected in the *B4GALNT2* wt transfected sample, where instead the Sd^a^ modified glycan was dominating (Figure 4D). Furthermore, N-glycopeptides from seven additional proteins, which were shared between the five HEK293 preparations, were identified, all demonstrating the presence of the Sd^a^ epitope in the *B4GALNT2* wt, rs148441237 and rs61743617 transfected samples, but lacking in the mock and in the rs7224888 transfected samples (Figure 5 and Table 1).

O-glycopeptides were also identified in these HEK293 transfectants (Table 1). One example is given from transferrin receptor protein 1 (TFR1), where a glycopeptide with a disialo core 1 O-glycan was identified in the mock transfected sample (Figure 4E). In the wt *B4GALNT2* transfected cells, the same peptide appeared, but now with the addition of one more HexNAc in the glycan chain, in line with the Sd^a^ epitope (Figure 4F). Additionally, here the diagnostic ion at *m*/*z* 860.31 appeared, demonstrating the NeuAcHexHexNAc_2_ composition of this glycan fragment. The same glycopeptide of TFR1 carrying the Sd^a^ epitope was identified in the rs148441237 and rs61743617 transfected cells but was not detected in the rs7224888 transfected sample (Figure 5). In addition, two other glycopeptides, from glypican-4 (GPC4) and protein FAM3C showing the same O-glycan carrying the Sd^a^ epitope, were identified. In summary, the Sd^a^ glycan epitope was structurally confirmed mass spectrometrically on complex and hybrid type N-glycans, as well as on core 1 O-glycans (for further details, see Figure 5 and Table 1), equating the results of the flow cytometry analyses.

### 2.3. The B4GALNT2 Sequence of Cad Phenotype Donors

To explore the hypothesis that the Sd^a^-high-expressing Cad phenotype may arise from a hyperactive variant of β4GalNAc-T2, or one with a broader acceptor preference, the *B4GALNT2* gene was sequenced in five individuals phenotyped as Cad. This investigation covered the coding region (exons 1–11), including both the long and short alternative exons 1, corresponding to the long and short transcripts, respectively (GenBank accession numbers AJ517770 and AJ517771). These samples had no alterations in common that could account for their Cad status. One sample was heterozygous for the splice-site mutation rs72835417:G>A previously found heterozygously in two Sd(a−) individuals, one in each of the two hitherto presented studies [1,2]. In the latter study, this SNP was only mentioned in the poster presentation (29th Regional Congress of the International Society of Blood Transfusion, in Basel, Switzerland on 22–26 June 2019) [2]. In the search for regulatory variants, we also analyzed the sequence upstream of the *B4GALNT2* gene covering an area of approx. 2000 nucleotides 5’ of the first base pair in exon 1 of AJ517770. Again, no difference in common for the investigated samples was seen compared to the wt reference gene. Blood samples were available from two individuals with the Cad phenotype, here referred to as samples Cad-a and Cad-b, respectively. Attempts were made to establish the relative quantity of *B4GALNT2* transcripts in RNA extracted from these samples and draw-date-matched control samples from two regular Sd(a+) donors. However, no *B4GALNT2* transcripts could be detected in any of the samples (data not shown). As a positive control, transcripts were detectable by the assay in the gastric cell line MKN-45. All samples had well detectable levels of housekeeping genes.

### 2.4. Characterization of the Sd^a^ Epitope on Erythrocytes from Donors with the Cad Phenotype

**Figure 6:**
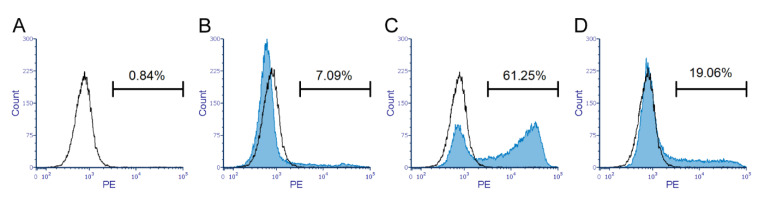
Analysis of Sd^a^ expression on erythrocyte surfaces by flow cytometry. Fixed erythrocytes were stained with human plasma containing anti-Sd^a^ (diluted in PBS) followed by PE-conjugated Goat anti-human:IgM. Erythrocytes from blood donors of (**A**) Sd(a–) phenotype (black line), also seen in the following graphs for comparison with other phenotypes. (**B**–**D**) Blue curve represents the common Sd(a+) phenotype (**B**) and Cad phenotypes, as shown by the Cad-a sample (**C**) and Cad-b (**D**).

The characteristic anti-Sd^a^ binding to erythrocytes from individuals of different SID phenotypes is displayed in the histograms of Figure 6A–D. Only a small proportion of the erythrocytes from common Sd(a+) individuals are stained by anti-Sd^a^ (Figure 6B showing a representative sample from a random donor). When staining erythrocytes of the Sd(a++)/Cad phenotype, a much larger proportion is stained (Figure 6C,D). However, as shown, the Sd^a^ expression varies significantly between the Cad-a and Cad-b individuals.

**Figure 7:**
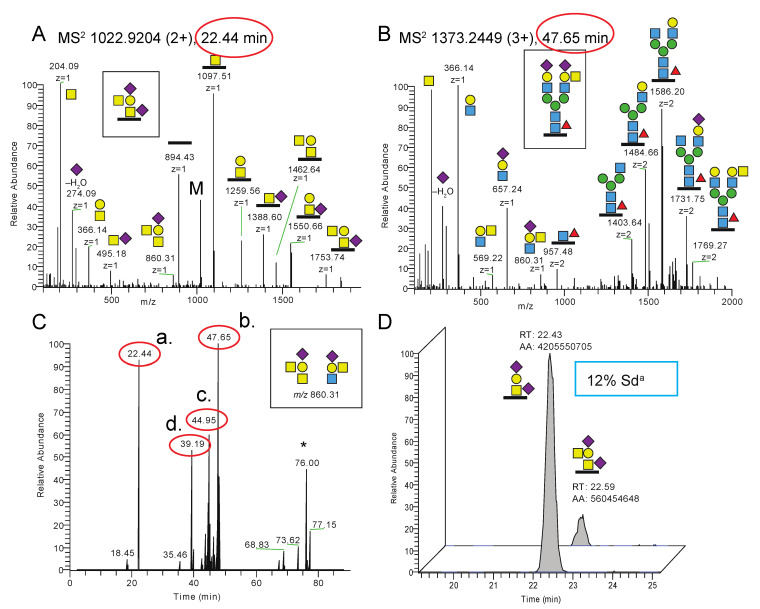
Glycoproteomic analysis of erythrocyte glycoproteins of a Cad sample. (**A**) MS^2^ spectrum of a glycopeptide DTYAATPR (residues 51–58) of GLPA carrying an O-glycan Sd^a^ epitope from the Cad-a sample. M denotes remaining precursor ions at *m*/*z* 1022.92. The MS^2^ spectrum of the corresponding non-Sd^a^ O-glycopeptide is shown in Supplementary Figure S2A. (**B**) MS^2^ spectrum of a glycopeptide LSVPDGFKVSNSSAR.G (residues 632–645) of B3AT with an N-glycan with the Sd^a^ epitope on one of the antennae obtained from the Cad-a individual. (**C**) Extracted MS^2^ ion chromatogram of the diagnostic ion at *m*/*z* 860.31 (occasionally measured as *m*/*z* 860.32, the exact mass is 860.3143 u). The major peaks (red encircled a.–d.) are indicated and identified in panels A and B and in the Supplementary Figure S1C–E. (**D**) Extracted MS^1^ ions of the GLPA glycopeptide (residues 51–58) demonstrated a relative peak intensity of 12% of the Sd^a^ epitope for the Cad-a sample. * Not glycopeptide related.

To characterize the Cad phenotype further, we used our Sd^a^ glycoproteomic methodology on erythrocyte (white ghost) membranes from two individuals with the Cad phenotype (Cad-a and Cad-b), along with ABO-matched non-Cad control samples, i.e., from Sd(a+) individuals. This was accomplished in order to investigate whether differences in the Sd^a^ expression could be identified at the glycoproteomic level and to identify membrane glycoproteins carrying the Sd^a^ epitope. Indeed, as observed for the *B4GALNT2*-transfected HEK293 cells, we identified the Sd^a^ epitope residing on a disialo core 1 O-glycan, this time on a peptide of glycophorin A (GLPA), residues 51–58, from the Cad-a individual (Table 2, Figure 7A). The Sd^a^ epitope was additionally identified on a disialylated N-glycopeptide from band 3 anion transport protein (B3AT) of the same sample (Figure 7B). The diagnostic MS^2^ ion at *m*/*z* 860.31 convincingly showed the presence of the Sd^a^ epitope in both types of glycans, and when this ion was screened for throughout the whole LC-MS/MS chromatogram (Figure 7C), it was obvious that these two glycopeptides represented the two major carriers of the Sd^a^ epitope in the sample (red encircled a. and b. annotations).

Additional glycopeptides carrying the Sd^a^ epitope (Figure 7C, red encircled c. and d. annotations) were identified from glycopeptides of GLPA (residues 59–80) and of equilibrative nucleoside transporter 1 (ENT1, also known as SLC29A1 or UniProt ID S29A1, residues 59–73) (Supplementary Figure S1).

In order to roughly estimate the relative amounts of the Sd^a^ epitope vs. its glycan precursor structure linked to an identical peptide, XICs of the two glycopeptide MS^1^ precursor ions were analyzed, the AUCs integrated and compared. Although it is not possible to assess the exact relative abundances of different peptide glycoforms since the HILIC, ionization and detection efficiencies are not the same for different glycoforms, the relative peak intensities of glycoforms within the same LC-MS experiments provide valuable and reproducible indications of their relative distribution in the sample. For the Cad-a individual, the relative peak intensity of Sd^a^ was 12% measured in two separate preparations for the GLPA glycopeptide (residues 51–58 (Figure 7D)) and ~10% for the other GLPA glycopeptide (residues 59–80 (Supplementary Figure S1A–D)). In contrast, the same Sd^a^ glycopeptides could neither be detected for the Cad-b individual nor for the Sd(a+) control individuals. However, although of low intensity, conclusive identification of Sd^a^ epitope from the Cad-b individual was obtained from a sample that was digested with pronase in which we identified a GLPA glycopeptide (residues 68–76), and was thus part of the longer 59–80 sequence, which carried the Sd^a^ epitope at ~0.1% peak intensity level (Supplementary Figure S2B–D).

When quantifying, in a similar manner, the Sd^a^ epitope of the identified N-glycopeptides of B3AT (Figure 7B) of the Cad-a sample, the modified glycan had a relative peak intensity of 18% (Supplementary Figure S3). However, a NeuAc_2_Hex_5_HexNAc_5_Fuc glycan was also identified in the control samples, but in MS^2^ of this glycopeptide the ion at *m*/*z* 860.31 was lacking (Supplementary Figure S4A) and showed the presence of a bisecting GlcNAc (Supplementary Figure S4B). This indicates that additional glycoforms with NeuAc_2_Hex_5_HexNAc_5_Fuc composition, most likely originating from a bisecting GlcNAc structure, are co-eluting and contribute to the precursor ions (Supplementary Figure S3D). Interestingly, the disialo biantennary N-glycopeptides from the Cad-a erythrocytes (Supplementary Figure S3A), but also from the control samples, were shown to carry a mix of NeuAcα2,3 and NeuAcα2,6 terminated structures (Supplementary Figure S3B,C) [26]. This agrees with the facts that we observed only one Sd^a^ epitope for the complex biantennary N-glycans of these erythrocyte samples and that NeuAcα2,6 is not part of the Sd^a^ epitope. In addition, this contrasts with TM9S3 of the *B4GALNT2*-transfected HEK293 cells which contained only NeuAcα2,3 terminated structures and for which we actually observed Sd^a^ epitopes on both antennae (Figure 4B and Figure 5).

## 3. Discussion

The Sd^a^ histo-blood group antigen and its null phenotype has long withstood attempts to understand its underlying genetic basis. In addition, structural and functional aspects of this glycan are poorly understood. After the cloning of a candidate Sd^a^ synthase more than 15 years ago, we have now proven that polymorphism in this gene affects Sd^a^ expression. Our results demonstrate that rs7224888:T>C is not only commonly found in Sd(a−) individuals but truly is the causative alteration that abolishes the formation of the Sd^a^ epitope. This missense mutation is situated in exon 10 and leads to the amino acid change where a cysteine is replaced by arginine at p.406 (in AJ517771) or p.466 in the enzyme utilizing the long exon 1 (AJ517770) in the globular catalytic domain of the enzyme. While the transferase is detected in cells transfected with this allele, but not its expected carbohydrate product, it is likely that the amino acid shift causes a functional alteration, severely damaging the enzymatic activity of the glycosyltransferase. The β4GalNAc-T2 homologue, human β4GalNAc-T1 (also called GM2 synthase) encoded by *B4GALNT1,* has been evaluated for its structural conformation dependence on its cysteine residues [24]. The homologue occurs as a homodimer and can be found in a soluble form, [25], in analogy with β4GalNAc-T2 where soluble forms are found in urine and plasma from humans [27,28]. All the cysteines of the soluble β4GalNAc-T1 are involved in disulfide bonds, four in intersubunit (dimer) formation and two in intrasubunit bonds. The spacing between all these cysteine residues is conserved, comparing the two enzymes (Supplementary Figure S5). The p.406Cys affected by rs7224888:T>C is highly conserved among several species, as we showed by sequence alignments from multiple species in our previous study [1], and this cysteine corresponds to one of the intrasubunit disulfide bonds in soluble β4GalNAc-T1 (Supplementary Figure S5). The results of the nonreduced immunoblot on lysates from transfected cells support that β4GalNAc-T2 is also involved in homodimer formation, which is almost entirely lost when p.Cys406Arg is introduced (Figure 3). It is therefore reasonable to hypothesize that the role of this cysteine is quite plausibly the same in Sd^a^ synthase as that described for GM2 synthase, and replacement with the large and positively charged arginine must have major consequences for structure and function of the *B4GALNT2*-encoded enzyme.

The structure of β4GalNAc-T2 has not yet been determined by x-ray crystallography or other types of in vitro experiments. To visualize the location of p.Cys406 and other residues affected in the mutants studied here, we interrogated the AlphaFold protein structure database and found a structure model for the long form of β4GalNAc-T2. The catalytic domain is predicted with a high degree of confidence (>90) as judged by the per residue confidence metric (predicted local distance difference test, pLDDT), given on a scale from 0 to 100. In Supplementary Figure S6, an overview of the molecule (Figure S6A–D), its pLDDT values (Figure S6C,D), DXD motif (Figure S6C) and the implicated amino acid positions studied here (Figure S6A–I) are shown, as well as close-ups of each residue which is altered in the mutants. It is particularly interesting to note that AlphaFold predicts a disulfide bridge formed between p.466Cys and p.513Cys, in full analogy with GM synthase (Supplementary Figures S5 and S6B,E,F).

The two rare SNPs, rs148441237:A>G and rs61743617:C>T, that were found in a compound heterozygous Sd(a−) individual, cause amino acid changes not too far from the predominant SNP just discussed (p.406Cys>Arg), namely p.376Glu>Arg and p.463Arg>Trp (Supplementary Figure S6G–I, respectively). Surprisingly, our study shows that overexpression of these two variants produces Sd^a^ epitopes in HEK293 cells with profiles similar to the wt β4GalNAc-T2. This is in sharp contrast to the predominant Sd(a−) allele with rs7224888:T>C, for which all Sd^a^ expression was abolished. Accordingly, the situation for the two rare alleles remains to be explored. If these two SNPs truly do not affect the synthesis of Sd^a^ antigen, the explanation may be found elsewhere. It is possible that a causative regulatory element variant would occur in linkage disequilibrium with one or both of these missense mutations, as recently reported for another carbohydrate blood group antigen [29]. In our previous study, we identified one Sd(a−) individual without any crucial mutation in the coding region of *B4GALNT2*, which may also hint at a regulatory, noncoding defect beyond the scope of this study [1]. One could also speculate as to whether the β4GalNAc-T2 catalytic ability is dependent on homodimerization, as seems to be the case for β4GalNAc-T1 [24]. If so, one could even speculate that the combination of the two different rare alleles could be so unique that the two different β4GalNAc-T2 variants together are incapable of forming a functional unit. However, we would have to assume that if such dimers are required, homodimer formation would not be affected. The co-transfection of the two rare alleles did not indicate that their products would interfere with the functionality of Sd^a^ synthesis. The co-transfection experiment was only performed once and did not include mass spectrometric evaluation or immunoblotting, but the flow cytometric result was virtually identical to the wt construct and the results obtained when each of the rare mutations was assessed separately.

Our LC-MS/MS data show that the Sd^a^ epitope is found on a broad range of membrane glycoproteins from the *B4GALNT2-*transfected HEK293 cells and carried on both *N*- and O-linked glycans (Table 1). This confirms what is known about the human transferase in different tissues, i.e., that it is capable to build upon Neu5Acα2,3Gal both on N-glycans, as in the case of Tamm–Horsfall protein in urine [15], and upon *O*-GalNAc initiated glycans, as seen in the colon as well as on Cad erythrocytes [12,14]. A hypothesis about qualitative differences between the wt enzyme and the two rare variants discussed above could be that they have slightly different acceptor preferences. However, LC-MS/MS did not pinpoint any major differences between the glycan structures of glycoproteins identified in these cell preparations. If anything, the wt showed slightly fewer targets than the two Sd^a^-synthesizing but mutated constructs (Figure 5).

The Cad samples displayed no common crucial genetic alteration in the coding gene sequence or 2000 bp upstream of the gene. We cannot yet rule out that the genetic background of Cad lies in unknown regulatory regions of the *B4GALNT2* gene. Such regions can stretch much further than what has been investigated here and, in the case of Cad, it would be of interest to search systematically for enhancer elements. Methylation in areas of the gene and the transcription factors ETS1 and SP1 have been associated with regulation of *B4GALNT2* in malignant colon tissue [30,31,32] However, it is not known how this may affect Sd^a^ expression on erythrocytes. Among the Cad samples (Cad-a and Cad-b), it was interesting to see that one individual was heterozygous for rs72835417:G>A, the splice-site mutation in intron 8, found earlier heterozygously in two Sd(a−) individuals (1,2), and thereby suggested as a reason to lack the antigen. This is of course still possible, as the genotype that causes the Cad phenotype could be a dominant trait carried on the other allele. Besides, further studies of the rs72835417:G>A allele are needed to determine its functional consequences on Sd^a^ synthesis.

It is also possible that Cad depends on a *B4GALNT2*-independent genetic trait, a hypothesis that would require a genome-wide approach, which is beyond the scope of this study. One can also speculate on whether the access to precursor substrate for β4GalNAc-T2 can differ in some people. It is interesting to note that Cad is more common in Asia and that there is an association between Sd^a^ expression and malaria susceptibility. *B4GALNT2* was also recently identified as the key inhibitory factor for avian influenza A [20], so the variable frequency of Cad in different parts of the world may have multiple evolutionary backgrounds.

The Sd^a^ antigen was also detected in the glycoproteome of membranes from erythrocytes with the Cad phenotype, but not in erythrocytes with the common Sd(a+) phenotype. This confirms earlier studies, as does our finding of the Sd^a^ epitope on GLPA from Cad erythrocytes [12,33]. Additionally, we identified B3AT and S29A1 as carriers of the Sd^a^ epitope on N- and O-glycans, respectively. These erythroid membrane proteins are the carriers of antigens in the Diego and Augustine blood group systems, respectively [34,35]. The peptide epitope has thereby been found on three proteins that are abundant in the erythrocyte membrane and considered to be endogenously synthesized [34,35]. Finding the Sd^a^ epitope on these proteins suggests an erythroid origin, which appears to contradict data that indicate *B4GALNT2* not to be expressed in erythroid tissue [23]. Based on this, we hypothesized that erythroid *B4GALNT2* expression could be restricted to individuals with the Cad phenotype. We were, however, not able to detect transcripts of the gene in whole blood from Cad individuals, but this could be a matter of sensitivity of the method and the available material.

The Sd^a^-decorated proportion of glycopeptides differed substantially between the two samples investigated. For the Cad-a and Cad-b samples, the glycoproteomic analysis showed a relative peak intensity of Sd^a^ glycans for GLPA glycopeptides of 10–12% vs. <0.5%, respectively. This mirrors the Sd^a^ histogram patterns of the stained erythrocytes in Figure 7C,D, where the latter express the epitope on a much smaller number of cells. In line with this observation, the Cad phenotype has historically been divided into subtypes 1–3, where Cad-1 defines erythrocytes that are most reactive and polyagglutinable, Cad-2 has been found in individuals of Asian origin and are only weakly (if at all) polyagglutinable. Finally, Cad-3 was found in two European families in whom polyagglutination does not appear to occur [36,37], although this has been disputed [38]. A previous study of the Sd^a^ epitope on GLPA in three different Cad donors also detected individual variations between the donors in the amount of glycans that carried the epitope. The strongest immunoreactive cells had indeed GLPA decorated with a larger number of Sd^a^ [12]. The two Cad samples in our study are both from Australian individuals of Chinese origin. Although the origin suggests both samples to be of subtype Cad-2, their subtype was not further characterized. It is nevertheless interesting that the relative amounts of Sd^a^ epitopes in erythrocytes of our Cad-a and Cad-b samples vary considerably and that the glycoproteomic methodology could be used so efficiently to distinguish them.

In summary, we evaluated the consequences of three *B4GALNT2* alleles previously found in Sd(a−) individuals, and we were able to present data that conclusively point out a relatively common polymorphism in *B4GALNT2* (rs7224888:T>C) as the main reason underlying complete deficiency of Sd^a^-containing glycans in humans. On the other hand, two rare variants (rs148441237:A>G and rs61743617:C>T) previously associated with the null phenotype did not appear to affect the synthesis of Sd^a^, as detected here. A more detailed investigation may be required to understand their role. We also provide genetic and biochemical insight into the high-expressing Sd(a++)/Cad variant, indicating that future studies of the erythroid regulation of *B4GALNT2* expression may offer an explanation to the underlying basis of this enigmatic phenotype.

## 4. Experimental Procedures

### 4.1. B4GALNT2 Overexpression in HEK293 Cells

Constructs with the *B4GALNT2* wt gene (GenBank accession no. AJ517771) or *B4GALNT2* mutated constructs based on the same sequence, but with SNPs, *B4GALNT2_*rs7224888:T>C, *B4GALNT2*_rs148441237:A>G or *B4GALNT2*_rs61743617:C>T in the bicistronic GFP expressing vector CS-2719-M61, were synthesized by GeneCopeia (Rockville, MD, USA) and distributed by Labomics (Nivelles, Belgium). The plasmid constructs were transformed into One Shot Top10 chemically competent *E. coli* (Thermo Fisher Scientific, Waltham, MA, USA) by adding 50 ng DNA to 25 µL competent cells, incubated on ice for 30 min, followed by a heat shock (42 °C for 30 s), then back on ice for 2 min. The cells were incubated in 250 µL SOB medium (10 mM NaCl, 2,5 mM KCl, 10 mM MgCl_2_, 10 mM MgSO_4_, 20 g/L Tryptone and 5 g/L Yeast extract) for 60 min at 37 °C while shaking. Cells were then spread on SOB–agar plates with ampicillin and cultured overnight (16 h) at 37 °C. Single colonies were picked for further growth in a starter cultures of 2 mL SOB with ampicillin for ca 6 h at 37 °C. The cultures were diluted 1:500 in 25 mL SOB and cultured overnight. The plasmid constructs were extracted using the plasmid midi kit from Qiagen (Hilden, Germany). The sequences were controlled applying Sanger sequencing by Eurofins Genomics (Ebersberg, Germany), with primers M61-F (5´-GCGGTAGGCGTGTACGGT) and M61-R (5´-AGCAGTCCCCAAGTCAGT).

The constructs were transfected into human embryonic kidney cell line HEK293, as this cell line shows no or low levels of endogenous *B4GALNT2* expression and Sd^a^ [17]. Transfections were performed on cell cultures at 80% confluency, plated 24 h previously. JetPRIME transfection reagent from Polyplus transfection (Illkirch, France) was used according to manufacturer’s protocol for cultures in 24- or 6-well plates. The cells were cultured in DMEM high glucose medium (Thermo Fisher Scientific) with 10% FBS. Transfected cells were harvested 24 h post transfection by detaching the adherent cells in TrypLE Express (Thermo Fisher Scientific) for 5 min at 37 °C after rinsing in PBS. For LC-MS/MS and sodium dodecyl sulfate polyacrylamide gel electrophoresis (SDS-PAGE)/Western blot GFP+ cells were sorted on BD FACSAria III (Becton, Dickinson and Company, Franklin Lakes, NJ, USA) 48 h post transfection and, once readily growing, cultured with selection pressure at 500 µL/mL Geneticin (Thermo Fisher Scientific).

### 4.2. Blood Samples

Anonymized whole blood samples were obtained from the Australian Red Cross Lifeblood (Brisbane, Queensland, Sydney, New South Wales and Perth, Western Australia, Australia) and from in-house collection reagent test erythrocytes and anonymized donor blood from the blood group reference laboratory in Lund, Sweden. A Sd(a−) test erythrocyte preparation from a commercially available rare cell panel (special panel from the German Red Cross Blood Donor Service Baden-Wuerttemberg—Hessen, Baden-Baden, Germany) was used as a negative control in flow cytometry experiments. Four Australian Cad samples (from individuals of Asian origin) were initially identified in the routine automated PK Olympus blood grouping reaction (DIAGAST, Loos, France) and further investigated at the Red Cell Reference Laboratory, Clinical Services and Research, Australian Red Cross Lifeblood, Kelvin Grove, Australia and in the blood group reference laboratory at the Department of Clinical Immunology and Transfusion Medicine in Lund, Sweden. Briefly, an apparent discrepancy in the routine ABO blood grouping reactions between forward (erythrocytes) and reverse (plasma) typing led to further evaluation of the underlying phenotype. In Australia, the serology was first investigated with a polyagglutination kit (Inverclyde Biologicals, Bellshill, Scotland, UK), DBA, papain treatment, Hydatid cyst fluid (HCF) and guinea pig urine (GPU) from Serum, Cells and Rare Fluids (SCARF) distribution scheme, *Helix pomatia* agglutinin (HPA) from Immucor Medizinische Diagnostik GmbH (Dreieich, Germany) and 10 different polyclonal Anti-A (in-house from donor plasma). Subsequent investigation of the erythrocytes and Cad phenotyping in Sweden included additional reagents: DBA (Immucor, Norcross, GA, USA), monoclonal Seraclone anti-A1 (Bio-Rad), a panel of lectins from Gamma Biologicals/Immucor including *Salvia horminum*, *Arachis hypogea*, *Glycine soy* and *Salvia sclarea*. Additionally, monoclonal anti-Sd^a^ antibody (clone KM694, Kyowa, BioFrontier Laboratories, Tokyo, Japan) and pooled human urine was used. In flow cytometry, the FITC-conjugated lectin *Helix pomatia*-stained proportion of the erythrocyte population was used as a measure to differentiate common Sd(a+) from Cad status. The serological investigation ruled out other causes of polyagglutination, as well as ABO and FORS blood group systems’ variants, and concluded the irregular, original reaction to be due to the Cad phenotype. Erythrocytes from Sd(a+) donors were analyzed for comparison in glycoproteomic analysis, flow cytometry experiments and detection of *B4GALNT2* transcripts. These were from random Swedish donors or draw-date-matched donors sent together with the Cad-b sample from Australia.

In-house reagents consisting of human plasma samples from Sd(a−) individuals with anti-Sd^a^, used for flow cytometry, were obtained as a kind gift from the Hoxworth Blood Center´s Immunohematology Reference Laboratory (ORL, Cincinnati, OH, USA).

### 4.3. Preparation of Erythrocyte (White Ghost) Membranes

The erythrocytes were lysed and repeatedly washed in cold hypotonic buffer (310 buffer, 100 mM NaH_2_PO_4_, 155 mM Na_2_HPO_4_ and diluted 1:20.5 in H_2_O), until the supernatant was neutral in color and a white fluffy pellet of membranes remained. The membranes were stored at −80 °C.

### 4.4. Flow Cytometry

Transfected HEK293 cells (approximately 4 × 10^5^) were stained with biotinylated DBA (Vector laboratories Inc., Burlingame, CA, USA), diluted 1:400,000 for 30 min at 4 °C in 50 µL FACS buffer (PBS with 1% BSA). The cells were washed and incubated with APC-conjugated streptavidin (Thermo Fisher Scientific), diluted 1:1000 for 30 min at 4 °C. The experiment was repeated three times with three biological replicates in each experiment. For Sd^a^ staining, HEK293 cells or glutaraldehyde (0.1%) fixed erythrocytes were incubated in 50 µL plasma (diluted 1:1 in PBS) for 30 min at room temperature while agitating. After washing in PBS, secondary antibody was added, polyclonal Alexa fluor 647 or phycoerythrin (PE)- conjugated goat anti-human IgM from Jackson ImmunoResearch (West Grove, PA, USA). Incubation conditions were 10 min at room temperature. The staining protocol with human anti-Sd^a^ included a separate incubation with ABO-matched control plasma without anti-Sd^a^. In prior analysis, the HEK293 cells were washed and filtered through 50 µm cup filters (Becton, Dickinson and Company). The GFP and APC/Alexa fluor 647 signals were recorded with BD FACSCanto II (Becton, Dickinson and Company) and the data analyzed in FCS express 6 (De Novo Software, Glendale, CA, USA). For statistical analysis, nonparametric Mann–Whitney U test was performed using the GraphPad prism software v. 8.4.2. (San Diego, CA, USA).

### 4.5. SDS-PAGE and Western Blotting of β4GalNAc-T2

Transfected cells were washed in PBS and one million were pelleted and lysed directly in 50 µL sample buffer, Laemmli (Bio-Rad, Hercules, CA, USA), with or without 5% β-mercaptoethanol (Sigma Aldrich, St. Louis, MA, USA). The lysates were sonicated and incubated for 5 min at 99 °C and SDS-PAGE performed as described elsewhere [39]. In brief, the proteins, samples and standard (precision plus protein dual xtra prestained standard) were separated at 300 V in SDS-PAGE on Mini-PROTEAN any-kD Tris-Glycine extended stain-free precast gel (Bio-Rad), activated by ultraviolet light (UV) in a ChemiDoc Touch camera and transferred to low-fluorescence polyvinylidene difluoride (PVDF) membranes. Total protein image was obtained by UV-light exposure of the PVDF-membrane, which then was subjected to blocking overnight in Casein ×1 solution (Vector laboratories) diluted in H_2_O. Blotting was achieved by incubating the membrane in blocking buffer and the polyclonal rabbit IgG anti-β4GalNAc-T2 (Sigma-Aldrich) diluted to 1:1000 at room temperature for two hours. The membrane was washed in TBS-T and incubated one hour at room temperature with Horseradish peroxidase (HRP)-conjugated polyclonal goat anti-Rabbit IgG (Bio-Rad) diluted to 1:3000. Enhanced chemiluminescent reagents (Bio-Rad) were used to develop the blot, which then was visualized in the ChemiDoc Touch camera as well as a colorimetric image of the standards. The images were annotated in Image Lab Software v 6.1. The SDS-PAGE equipment, camera and software were from Bio-Rad.

### 4.6. Sample Preparation of Cells for LC- MS/MS Analysis

Transfected cells were plated at 5 million cells per 10-cm-diameter dish and cultured until cells reached circa 80% confluency. The adhering cells were rinsed three times in PBS, lysed with 50 mM triethylammonium bicarbonate (TEAB) buffer with 2% SDS and the cell debris detached with a scraper, transferred to tubes and stored at −80 °C. The frozen cell lysates were sent to BioMS, the national infrastructure node for glycomics and glycoproteomics (University of Gothenburg, Sweden), for further sample preparation.

The lysates from either transfected cells or erythrocyte membranes were subjected to filter-aided sample preparation (FASP) with trypsin digestion. Briefly, samples were reduced with 100 mM dithiothreitol at 60 °C for 30 min, and then applied to 30 kDa MWCO filters (Pall Nanosep, Sigma-Aldrich). After centrifugation, the retentate was washed with 8 M urea and then with 0.5% sodium deoxycholate (SDC) in TEAB buffer. Cys residues were methylthio derivatized with 10 mM methyl methanethiosulfonate in 0.5% SDC in TEAB buffer at room temperature for 20 min. The trypsin digestion was performed on the MWCO filter with 4 µg trypsin (Pierce MS grade, Thermo Fisher Scientific) in 0.5% SDC in TEAB buffer at 37 °C overnight, with a new trypsin addition over 2 h the following day. Cleavages were also performed with chymotrypsin and proteinase K (sequencing grade, Promega) on selected samples. After centrifugation of the MWCO spin column, the filtrate was saved and SDC was precipitated by acidification using 10% trifluoroacetic acid (TFA), and the supernatant was saved after centrifugation.

The cleaved samples were desalted with C18 spin columns and dissolved in 80% acetonitrile/1% TFA in water, and glycopeptides enriched with hydrophilic interaction liquid chromatography (HILIC), with slight modifications [40]. Briefly, the samples were applied to in-house packed cartridges containing 20 mg Zic-HILIC particles (10 μm, 200 Å; Sequant/Merck). The flow-through was collected and re-applied three times. The column was then washed with 1.2 mL of 80% acetonitrile and 1% TFA in water. Enriched glycopeptides were eluted with 4× of 50 µL 0.1% TFA, 50 μL of 25 mM ammonium bicarbonate in water and, finally, 50 μL of 50% (*v*/*v*) acetonitrile in water and dried by vacuum centrifugation.

### 4.7. LC-MS/MS Analysis

The samples were dissolved in 20 µL 0.2% formic acid, 3% acetonitrile in water and analyzed on an Orbitrap Lumos Tribrid mass spectrometer interfaced with Easy-nLC1200 liquid chromatography system (Thermo Fisher Scientific). An Acclaim PepMap 100 C18 trap column (100 μm × 2 cm, particle size 5 μm, Thermo Fischer Scientific) was used and peptides were separated at 300 nL/min on an in-house packed analytical column (75 μm × 30 cm, particle size 3 μm, Reprosil-Pur C18, Dr. Maisch) using a linear gradient of 7–35% of solvent B in solvent A over 75 min, then an increase to 100% of solvent B for 5 min and, finally, 100% of solvent B for 10 min. Solvent A was 0.2% formic acid in water and solvent B was 80% acetonitrile and 0.2% formic acid in water. The precursor ion scans were performed at a resolution of 120,000 and the *m*/*z* range was 600–2000. The most abundant precursor ions with a positive charge (z) of 2–7 were selected for MS/MS (MS^2^) over 3 s with a 5 *m*/*z* unit isolation window and subjected sequentially to higher-energy collision induced dissociation (HCD) at normalized collision energies (NCEs) of 20%, 30% and 40%. The MS^2^ spectra were detected in the Orbitrap in centroid mode at 30,000 resolution.

### 4.8. Glycoproteomic Analysis

The LC-MS/MS raw files were analyzed with the Byonic software (Protein Metrics) using a modified list of the modifications “182 human N-glycans” and “6 most common O-glycans”. For instance, N-glycan modifications with NeuAc_2_Hex_5_HexNAc_6_ and NeuAc_2_Hex_5_HexNAc_6_dHex compositions were added, and O-glycan modifications of NeuAcHexHexNAc_2_ and NeuAc_2_HexHexNAc_2_ were added. Additional search criteria included use of the UniProt *Homo sapiens* database (20,369 sequences), C-terminal cleavage after Lys and Arg, two missed cleavages were allowed, accuracy for the MS^1^ precursor ion was set to 10 ppm and for MS^2^ ions it was 20 ppm static modification was a methylthio group on Cys (+45.9877 u), and variable modification was oxidation of Met. For the chymotrypsin digested samples, C-terminal cleavage after Phe, Tyr, Trp, and Leu, with two allowed missed cleavages, were applied. For the pronase digested samples, all possible cleavages were considered, and the protein database was composed of selected glycoproteins: GLPA_HUMAN, GLPB_HUMAN, GLPC_HUMAN, GLPE_HUMAN, B3AT_HUMAN, TFR1_HUMAN, AQP1_HUMAN, RHD_HUMAN, and ACKR1_HUMAN.

For the trypsin and chymotrypsin Byonic analyses, the reversed sequences (decoy database) were included in the analysis, and a cut off score of 300 was used, which resulted in false discovery rates of 0.46–2.00% for the glycopeptide hits (Supplementary Tables S1, S2, S4 and S5). In addition, lower scores were also accepted for alternative glycoforms, which shared the same peptide and had the expected glycosidic fragmentation profile, with glycopeptides having Byonic scores >300 from the same sample. In addition, all glycopeptide MS/MS spectra were verified to contain the correct peptide + HexNAc ion for N-glycopeptides and the correct peptide ion for O-glycopeptides. For NeuAc hits, the diagnostic NeuAc oxonium ions at *m*/*z* 274.09 and *m*/*z* 292.10 had to be present. Extracted ion chromatograms (XICs) were produced with the Xcalibur software (Thermo Fisher Scientific) for the diagnostic ion (NeuAc HexHexNAc_2_)^+^ at *m*/*z* 860.31 to identify additional glycopeptides.

### 4.9. Gene Sequencing

Whole blood derived DNA was prepared by a simple salting out method [41]. Amplification was achieved using modified Expand high-fidelity PCR system (Roche Basel Switzerland) [42]. Briefly, DNA (ca 150 ng) was mixed with 0.2 U Taq Polymerase and 7 pmol of each primer, previously published [1], with the addition of SID-2301F (5′-TAGTTTCTGCCTGTAGCCC), in a total reaction volume of 20 µL. The PCR protocol was initiated for 3 min at 95 °C followed by 35 thermal cycles of 95 °C (20 s), 58 °C (30 s), and 72 °C (40 s or 3 min, depending on amplicon size). The PCR products were run on a 3% gel from which the correct sized amplicons were excised and purified with QIAquick gel extraction kit (Qiagen). Sanger sequencing was performed in house [42] or by Eurofins Genomics (Ebersberg, Germany). Sequences were analyzed in CodonCode aligner software v. 4.2.7 (Barnstable, MA, USA).

### 4.10. Real-Time Quantitative Polymerase Chain Reaction (qPCR)

Frozen whole blood in Trizol^®^ (Invitrogen) was thawed and RNA extracted according to the manufacturer’s instructions, followed by cDNA synthesis with SuperScript IV Vilo (Thermo Fisher Scientific). Real-time qPCR was performed using Taqman gene expressing assays Hs00963127_m1, Hs01060665_g1, and Hs02758991_g1 (Thermo Fisher Scientific), targeting *B4GALNT2*, *ACTB* (reference), and *GAPDH* (control), respectively. The gastric adenocarcinoma cell line MKN-45 cDNA was used as positive control.

### 4.11. β4GalNAc-T2 Structure Prediction

Three-dimensional molecular modelling of β4GalNAc-T2 (identifier AF-Q8NHY0-F1 including amino acids 1–566) was performed using the AlphaFold Protein Structure Database (developed by DeepMind and EMBL-EBI), and the resulting PDB file was downloaded and analyzed in PyMOL (version 2.3.4) [43,44,45]. Numbering of amino acids in the model is based on the long form, i.e., p.466Cys>Arg corresponds to p.406Cys>Arg in the short form.

### 4.12. Ethical Consideration

Ethical review and approval were not required for this study since only leftover, anonymized blood samples taken as part of routine blood donation from healthy blood donors were used. This is in accordance with the Swedish law (2003:460) on research on humans and biological material from humans.

## Figures and Tables

**Table 1:** Identified glycoproteins/glycopeptides carrying the Sd^a^ epitope in *B4GALNT2* wt-transfected HEK293 cells. Detailed information on precursor masses, charges and glycan compositions and additional glycopeptides encompassing the same glycosites are presented in Supplementary Tables S1 and S2. Annotated MS^2^ spectra are shown in the Supplementary Spectra collections 1 and 2.

UniProt ID *	Glycoprotein **	Glycopeptide ***	Glycosite
AT1B3	Sodium/potassium-transporting ATPase subunit beta-3	FLKPYTLEEQKNLTVCPDGALFEQK	Asn-124
CD276	CD276 antigen	TALFPDLLAQGNASLR	Asn-104
CD63	CD63 antigen	NNHTASILDR	Asn-130
4F2	4F2 cell-surface antigen heavy chain	DIENLKDASSFLAEWQNITK	Asn-365
FAM3C	Protein FAM3C	SALDTAARSTKPPR	Ser-42–Thr-51
GPC4	Glypican-4	FRPHHPEERPTTAAGTSLDR	Thr-375–Ser-381
LAMP1	Lysosome-associated membrane glycoprotein 1	GHTLTLNFTR	Asn-103
PTPRF	Receptor-type tyrosine-protein phosphatase F	KVEVEPLNSTAVHVYWK	Asn-721
TFR1	Transferrin receptor protein 1	DFEDLYTPVNGSIVIVR	Asn-251
TFR1	Transferrin receptor protein 1	LAGTESPVREEPGEDFPAAR	Thr-104–Ser-106
TM9S3	Transmembrane 9 superfamily member 3	IVDVNLTSEGK	Asn-174

* The UniProt IDs are shortened from the official format, i.e., AT1B3_HUMAN, etc. ** The glycan structures related to HEK293 cells transfected with different constructs are shown in Figure 5, and the glycopeptide hits are presented in Supplementary Tables S1–S3. *** Underlined amino acids define unambiguous glycosites.

**Table 2:** Identified glycoproteins/glycopeptides carrying the Sd^a^ epitope in RBCs from an individual with the Cad phenotype (Cad-a). Detailed information on precursor masses, charges and glycan compositions and additional glycopeptides encompassing the same glycosites are presented in Supplementary Tables S4 and S5. Annotated MS^2^ spectra are shown in the Supplementary Spectra collections 1 and 2.

UniProt ID *	Glycoprotein	Glycopeptide **	Glycosite
B3AT	Band 3 anion transport protein	LSVPDGFKVSNSSAR	Asn-642
GLPA	Glycophorin A	DTYAATPR	Thr-52–Thr-56
GLPA	Glycophorin A	AHEVSEISVRTVYPPEEETGER	Ser-63–Thr-77
S29A1	Equilibrative nucleoside transporter 1	DAQASAAPAAPLPER	Ser-63

* The UniProt IDs are shortened from the official format, i.e., B3AT_HUMAN, etc. ** Underlined amino acids define unambiguous glycosites.

## Data Availability

The MS data have been deposited to the ProteomeXchange Consortium via the PRIDE partner repository (https://www.ebi.ac.uk/pride/) with the dataset identifier PXD023943. MS files, scan numbers, and additional information for all the presented glycopeptides are provided in the Supporting Information Table S1 and at PRIDE.

## Supplementary Materials

The following supporting information can be downloaded at: https://www.mdpi.com/article/10.3390/ijms23073936/s1. References [46,47] are cited in the Supplementary Materials.

## Abbreviations

Nomenclature, abbreviations, and symbols for monosaccharide residues follow the IUPAC-adopted and NCBI-based Symbol Nomenclature for Glycans (https//www.ncbi.nlm.nih.gov/glycans/snfg.html) accessed on 5 February 2022. Protein abbreviations follow the UniProt IDs and are summarized in Table 1 and Table 2.

## References

[1] Stenfelt L., Hellberg Å., Möller M., Thornton N., Larson G., Olsson M.L. (2019). Missense mutations in the C-terminal portion of the B4GALNT2-encoded glycosyltransferase underlying the Sd(a-) phenotype. Biochem. Biophys. Rep..

[2] Veldhuisen B., Ligthart P., van der Mark-Zoet J., Javadi A., Tissoudali A., Dengerink I., Folman C., van der Shoot C.E. (2019). Identification of a single homozygous mutation in the B4GALNT2 gene in individuals lacking the Sd(a) (SID) antigen on red blood cells. Vox Sang..

[3] Macvie S.I., Morton J.A., Pickles M.M. (1967). The reactions and inheritance of a new blood group antigen, Sd^a^. Vox Sang..

[4] Renton P.H. (1967). Anti-Sd^a^ new blood group antibody. Vox Sang..

[5] Morton J.A., Pickles M.M., Vanhegan R.I. (1988). The Sda antigen in the human kidney and colon. Immunol. Investig..

[6] Morton J.A., Pickles M.M., Terry A.M. (1970). The Sda blood group antigen in tissues and body fluids. Vox Sang..

[7] Spitalnik S., Cox M.T., Spennacchio J., Guenther R., Blumberg N. (1982). The serology of Sd^a^ effects of transfusion and pregnancy. Vox Sang..

[8] Marcus D.M., Cass L.E. (1969). Glycosphingolipids with Lewis blood group activity: Uptake by human erythrocytes. Science.

[9] Peetermans M.E., Cole-Dergent J. (1970). Haemolytic transfusion reaction due to anti-Sd^a^. Vox Sang..

[10] Reznicek M.J., Cordle D.G., Strauss R.G. (1992). A hemolytic reaction implicating Sd^a^ antibody missed by immediate spin crossmatch. Vox Sang..

[11] Cazal P., Monis M., Caubel J., Brives J. (1968). Herediatry dominant polyagglutinability: Private antigen (Cad) corresponding to a public antibody and a lectin of Dolichos biflorus. Rev. Fr. Transfus..

[12] Blanchard D., Capon C., Leroy Y., Cartron J.-P., Fournet B. (1985). Comparative study of glycophorin A derived O-glycans from human Cad, Sd(a+) and Sd(a−) erythrocytes. Biochem. J..

[13] Blanchard D., Piller F., Gillard B., Marcus D., Cartron J.-P. (1985). Identification of a novel ganglioside on erythrocytes with blood group Cad specificity. J. Biol. Chem..

[14] Capon C., Maes E., Michalski J.C., Leffler H., Kim Y.S. (2001). Sd(a)-antigen-like structures carried on core 3 are prominent features of glycans from the mucin of normal human descending colon. Biochem. J..

[15] Donald A.S., Yates A.D., Soh C.P., Morgan W.T., Watkins W.M. (1983). A blood group Sda-active pentasaccharide isolated from Tamm-Horsfall urinary glycoprotein. Biochem. Biophys. Res. Commun..

[16] Fukuda M., Dell A., Oates J.E., Fukuda M.N. (1984). Structure of branched lactosaminoglycan, the carbohydrate moiety of band 3 isolated from adult human erythrocytes. J. Biol. Chem..

[17] Groux-Degroote S., Wavelet C., Krzewinski-Recchi M.A., Portier L., Mortuaire M., Mihalache A., Trinchera M., Delannoy P., Malagolini N., Chiricolo M. (2014). B4GALNT2 gene expression controls the biosynthesis of Sda and sialyl Lewis X antigens in healthy and cancer human gastrointestinal tract. Int. J. Biochem. Cell Biol..

[18] Cartron J.P., Prou O., Luilier M., Soulier J.P. (1983). Susceptibility to invasion by Plasmodium falciparum of some human erythrocytes carrying rare blood group antigens. Br. J. Haematol..

[19] Serafini-Cessi F., Monti A., Cavallone D. (2005). N-Glycans carried by Tamm-Horsfall glycoprotein have a crucial role in the defense against urinary tract diseases. Glycoconj. J..

[20] Heaton B.E., Kennedy E.M., Dumm R.E., Harding A.T., Sacco M.T., Sachs D., Heaton N.S. (2017). A CRISPR Activation Screen Identifies a Pan-avian Influenza Virus Inhibitory Host Factor. Cell Rep..

[21] Presti L.L., Cabuy E., Chiricolo M., Dall’Olio F. (2003). Molecular cloning of the human beta1,4 *N*-Acetylgalactosaminyltransferase responsible for the biosynthesis of the Sd^a^ histo-blood group antigen: The sequence predicts a very long cytoplasmic domain. J. Biochem..

[22] Montiel M.D., Krzewinski-Recchi M.A., Delannoy P., Harduin-Lepers A. (2003). Molecular cloning, gene organization and expression of the human UDP-GalNAc:Neu5Acalpha2-3Galbeta-R beta1,4-N-acetylgalactosaminyltransferase responsible for the biosynthesis of the blood group Sd^a^/Cad antigen: Evidence for an unusual extended cytoplasmic domain. Biochem. J..

[23] Jöud M., Möller M., Olsson M.L. (2018). Identification of human glycosyltransferase genes expressed in erythroid cells predicts potential carbohydrate blood group loci. Sci. Rep..

[24] Li J., Yen T.Y., Allende M.L., Joshi R.K., Cai J., Pierce W.M., Jaskiewicz E., Darling D.S., Macher B.A., Young W.W. (2000). Disulfide bonds of GM2 synthase homodimers. Antiparallel orientation of the catalytic domains. J. Biol. Chem..

[25] Jaskiewicz E., Zhu G., Bassi R., Darling D.S., Young W.W. (1996). Beta1,4-N-acetylgalactosaminyl-transferase (GM2 synthase) is released from Golgi membranes as a neuraminidase-sensitive, disulfide-bonded dimer by a cathepsin D-like protease. J. Biol. Chem..

[26] Pett C., Nasir W., Sihlbom C., Olsson B.M., Caixeta V., Schorlemer M., Zahedi R.P., Larson G., Nilsson J., Westerlind U. (2018). Effective Assignment of α2,3/α2,6-Sialic Acid Isomers by LC-MS/MS-Based Glycoproteomics. Angew. Chem. Int. Ed. Engl..

[27] Serafini-Cessi F., Malagolini N., Dall’Olio F. (1988). Characterization and partial purification of beta-N-acetylgalactosaminyltransferase from urine of Sd(a+) individuals. Arch. Biochem. Biophys..

[28] Takeya A., Hosomi O., Kogure T. (1987). Identification and characterization of UDP-GalNAc: NeuAc alpha 2-3Gal beta 1-4Glc(NAc) beta 1-4(GalNAc to Gal)N-acetylgalactosaminyltransferase in human blood plasma. J. Biochem..

[29] Westman J.S., Stenfelt L., Vidovic K., Moller M., Hellberg A., Kjellstrom S., Olsson M.L. (2018). Allele-selective RUNX1 binding regulates P1 blood group status by transcriptional control of A4GALT. Blood.

[30] Wang H.R., Hsieh C.Y., Twu Y.C., Yu L.C. (2008). Expression of the human Sd(a) beta-1,4-N-acetylgalactosaminyltransferase II gene is dependent on the promoter methylation status. Glycobiology.

[31] Pucci M., Malagolini N., Dall’Olio F. (2021). Glycosyltransferase B4GALNT2 as a Predictor of Good Prognosis in Colon Cancer: Lessons from Databases. Int. J. Mol. Sci..

[32] Wavelet-Vermuse C., Groux-Degroote S., Vicogne D., Cogez V., Venturi G., Trinchera M., Brysbaert G., Krzewinski-Recchi M.A., Bachir E.H., Schulz C. (2021). Analysis of the proximal promoter of the human colon-specific B4GALNT2 (Sd(a) synthase) gene: B4GALNT2 is transcriptionally regulated by ETS1. Biochim. Biophys. Acta Gene Regul. Mech..

[33] Blanchard D., Cartron J.P., Fournet B., Montreuil J., van Halbeek H., Vliegenthart J.F. (1983). Primary structure of the oligosaccharide determinant of blood group Cad specificity. J. Biol. Chem..

[34] Reid M.E., Lomas-Francis C., Olsson M.L. (2012). The Blood Group Antigen FactsBook.

[35] Daniels G., Ballif B.A., Helias V., Saison C., Grimsley S., Mannessier L., Hustinx H., Lee E., Cartron J.P., Peyrard T. (2015). Lack of the nucleoside transporter ENT1 results in the Augustine-null blood type and ectopic mineralization. Blood.

[36] Cazal P., Monis M., Bizot M. (1971). The Cad antigens and their relation to A antigens. Rev. Fr. Transfus..

[37] Sringarm S., Chiewsilp P., Tubrod J. (1974). Cad receptor in Thai blood donors. Vox Sang..

[38] Lopez M., Gerbal A., Bony V., Salmon C. (1975). Cad antigen: Comparative study of 50 samples. Vox Sang..

[39] Stenfelt L., Westman J.S., Hellberg Å., Olsson M.L. (2019). The P1 histo-blood group antigen is present on human red blood cell glycoproteins. Transfusion.

[40] Parker B.L., Thaysen-Andersen M., Solis N., Scott N.E., Larsen M.R., Graham M.E., Packer N.H., Cordwell S.J. (2013). Site-specific glycan-peptide analysis for determination of N-glycoproteome heterogeneity. J. Proteome Res..

[41] Miller S.A., Dykes D.D., Polesky H.F. (1988). A simple salting out procedure for extracting DNA from human nucleated cells. Nucleic Acids Res..

[42] Hellberg Å., Steffensen R., Yahalom V., Nilsson-Sojka B., Heier H.E., Levene C., Poole J., Olsson M.L. (2003). Additional molecular bases of the clinically important p blood group phenotype. Transfusion.

[43] Jumper J., Evans R., Pritzel A., Green T., Figurnov M., Ronneberger O., Tunyasuvunakool K., Bates R., Žídek A., Potapenko A. (2021). Highly accurate protein structure prediction with AlphaFold. Nature.

[44] Varadi M., Anyango S., Deshpande M., Nair S., Natassia C., Yordanova G., Yuan D., Stroe O., Wood G., Laydon A. (2022). AlphaFold Protein Structure Database: Massively expanding the structural coverage of protein-sequence space with high-accuracy models. Nucleic Acids Res..

[45] (2019). The PyMOL Molecular Graphics System, Version 2.3.4.

[46] Altschul S.F., Madden T.L., Schäffer A.A., Zhang J., Zhang Z., Miller W., Lipman D.J. (1997). Gapped BLAST and PSI-BLAST: A new generation of protein database search programs. Nucleic Acids Res..

[47] Altschul S.F., Wootton J.C., Gertz E.M., Agarwala R., Morgulis A., Schäffer A.A., Yu Y.-K. (2005). Protein database searches using compositionally adjusted substitution matrices. FEBS J..

