# Supplementary material for: Glycoproteomic and Phenotypic Elucidation of B4GALNT2 Expression Variants in the SID Histo-Blood Group System
Source: Int J Mol Sci. 2022 Apr 1;23(7):3936. doi: 10.3390/ijms23073936 (PMC8999409; doi:10.3390/ijms23073936)
Supplement: Supplementary file 1 [file ijms-23-03936-s001.zip › Stenfelt et al_SID_IJMS_Supplementary Figures S1-6_proof.pdf]

Supplementary Materials

Glycoproteomic and phenotypic elucidation of *B4GALNT2* expression variants in the SID histo-blood group system

Linn Stenfelt[1]*, Jonas Nilsson[2]**, Åsa Hellberg[3], Yew Wah Liew[4], Jenny Morrison[4], Göran Larson[5,6], Martin L Olsson[1,3]**

[1] Division of Hematology and Transfusion Medicine, Department of Laboratory Medicine, Lund University, Lund, Sweden
[2] Proteomics Core Facility, Sahlgrenska Academy at the University of Gothenburg, Gothenburg, Sweden
[3] Department of Clinical Immunology and Transfusion Medicine, Office for Medical Services, Region Skåne, Sweden
[4] Red Cell Reference Laboratory, Clinical Services and Research, Australian Red Cross Lifeblood, Kelvin Grove, Australia
[5]Laboratory of Clinical Chemistry, Sahlgrenska University Hospital, Gothenburg, Sweden
[6]Department of Laboratory Medicine, Institute of Biomedicine, Sahlgrenska Academy at the University of Gothenburg, Gothenburg, Sweden
*Currently at: Section for Protein Chemistry and Enzyme Technology, Department of Biotechnology and Biomedicine, Technical University of Denmark, Copenhagen, 2800 Kgs. Lyngby, Denmark

****Corresponding authors**:

Prof. Martin L Olsson, M.D., Ph.D.
E-mail: Martin_L.Olsson@med.lu.se

Jonas Nilsson, Ph.D.
E-mail: Jonas.GM.Nilsson@gu.se

Supplementary Figures S1-6

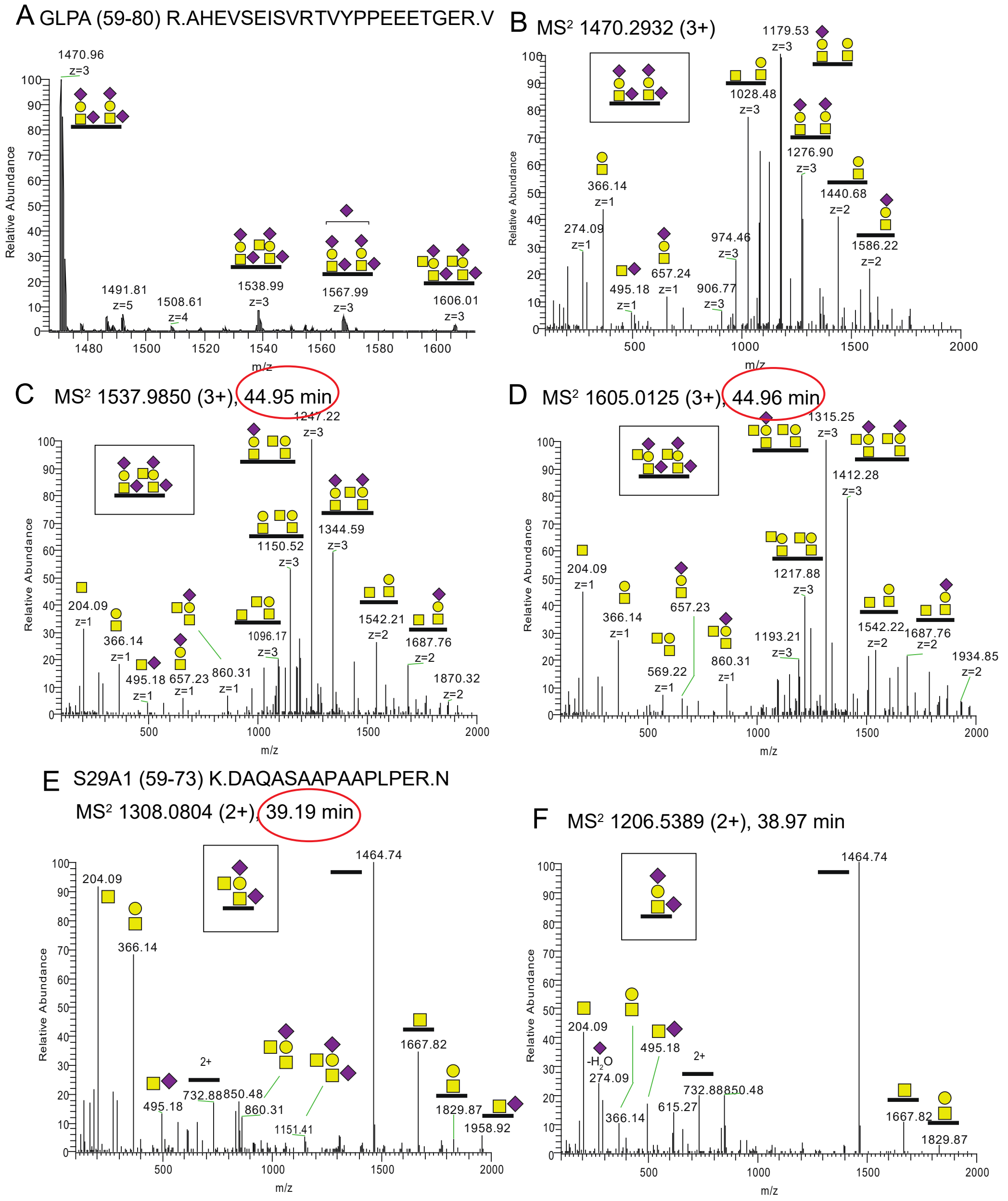

Supplementary Figure S1. Glycoproteomic analysis of O-glycopeptides carrying Sd^a in erythrocyte membranes (**A**) MS^1 of R.AHEVSEISVRTVYPPEEETGER.V (residues 59-80) of GLPA from the Cad-a sample carrying two O-glycans with 0-2 Sd^a epitopes. The relative peak intensities of the Sd^a glycoforms, calculated from extracted ion chromatograms (XICs) of the precursor ions, are ~10%. The MS^2 spectra of the precursor ions are shown in **B-D**. Only selective peaks are annotated. The *m/z* 860.31 ion typical for the Sd^a glycoforms at 44.95 and 44.96 min elution time are visualized in Fig. 7C (red encircled c.). The presence of *m/z* 657.23 in **D** is due to glycosidic fragmentation of the Sd^a epitope (oxonium ion at *m/z* 860.31) that loses the terminal HexNAc and leaves an ion at *m/z* 657.23, that further dissociates to *m/z* 366.14 etc. Such multiple fragmentations were observed in all disialylated core 1 and Sd^a core 1 O-glycopeptide spectra. (**E**) MS^2 spectrum of K.DAQASAAPAAPLPER.N (residues 59-73) of S29A1 carrying an O-glycan Sd^a epitope from the Cad-a sample and (**F**) the MS^2 spectrum of the corresponding glycopeptide with a disialylated core 1 O-glycan lacking the Sd^a epitope eluting slightly faster. The ion at *m/z* 860.31 of the Sd^a glycopeptide eluting at 39.19 min is annotated in Fig. 7C (red encircled d.).

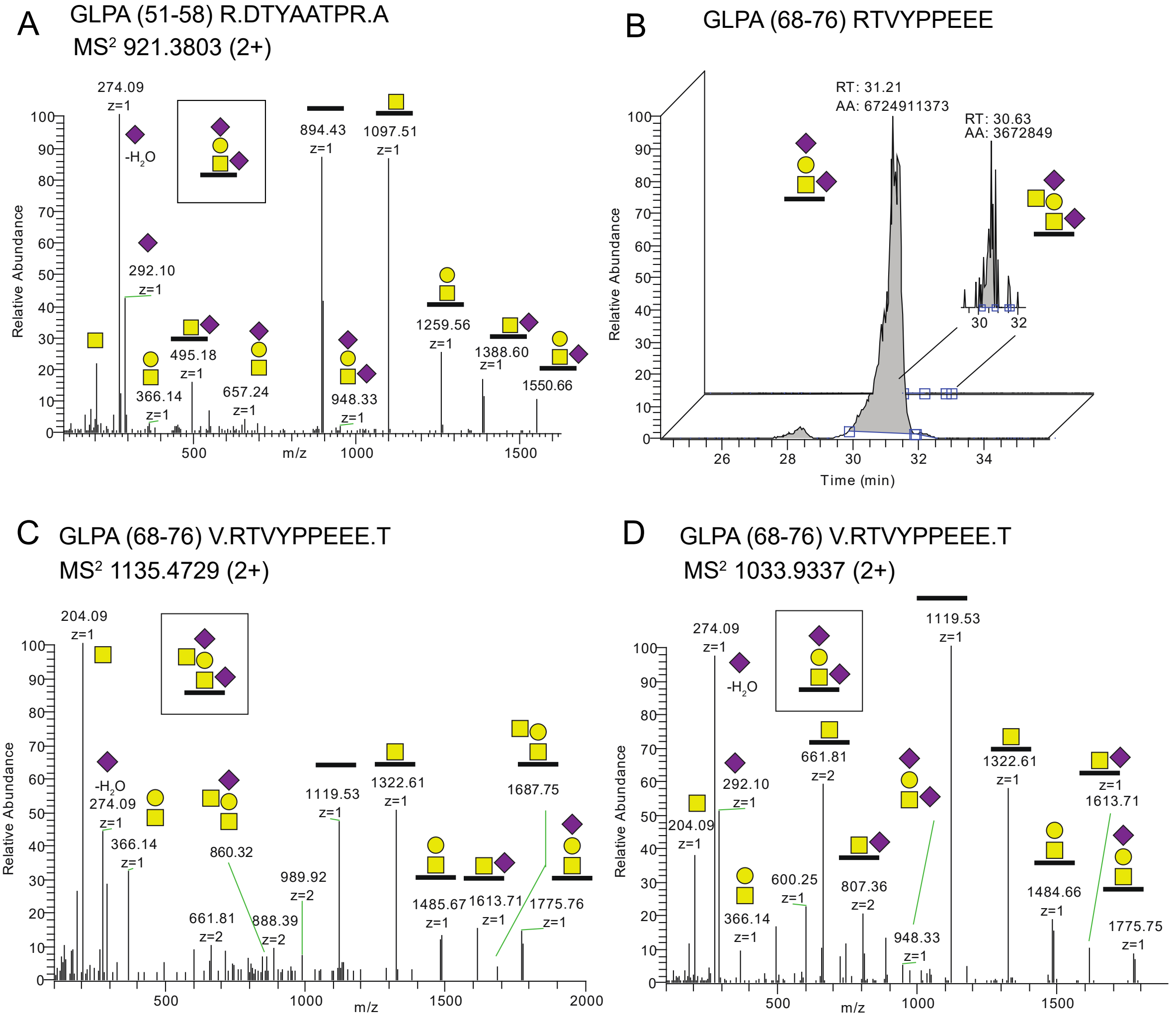

Supplementary Figure S2. Glycoproteomic analysis of O-glycopeptides carrying Sd^a in erythrocyte membranes (**A**) MS^2 spectrum of R.DTYAATPR.A (residues 51-58) of GLPA with the disialo core 1 O-glycan from the Cad-a sample. (**B**) A pronase-digested sample of Cad-b showing an alternative glycopeptide of GLPA (residues 68-76) carrying the Sd^a epitope at 0.1% relative precursor intensity. This glycopeptide was subjected to MS^2 confirming the Sd^a epitope structure (**C**), as was also the disialo core 1 O-glycopeptide (**D**).

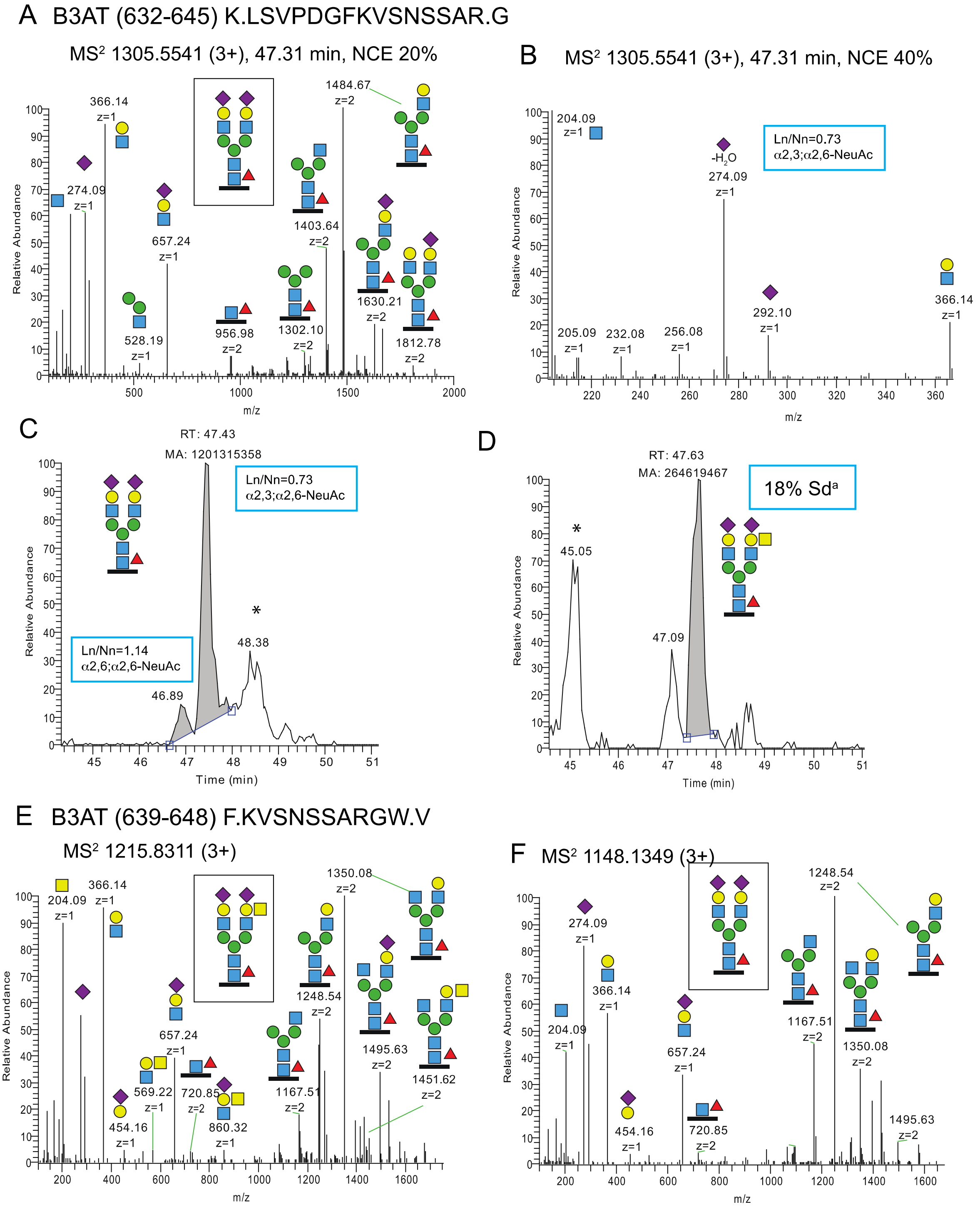

Supplementary Figure S3. Glycoproteomic analysis of band 3 anion transport protein (B3AT) N-glycopeptides. (**A**) MS^2 spectrum of K.LSVPDGFKVSNSSAR.G (residues 632-645) of B3AT carrying a core fucose and disialo biantennary N-glycan from the Cad-a individual. The corresponding MS^2 spectrum of the Sd^a glycoform is shown in Figure 7B. (**B**) MS^2 spectrum of the *m/z* 200-400 region taken at NCE 40% shows that the major eluting peak (47.43 min in **C**) had mixed NeuAcα2,3; NeuAcα2,6 terminals (Ln/Nn = 0.73). XICs of the corresponding glycopeptide precursor ions (**C**) without Sd^a (*m/z* 1305.55) and (**D**) with the Sd^a epitope (*m/z* 1373.24) showed that the relative peak intensity of Sd^a was 18%. An isobaric ion eluting at 47.09 min (in **D**) was not subjected to MS^2 and was excluded from the relative peak intensity calculation since it may have a non-Sd^a

glycan structure including a bisecting GlcNAc. The minor chromatographic peak at 46.89 min in **C** had NeuAcα2,6; NeuAcα2,6 terminals (Ln/Nn =1.14). The glycoproteomic discrimination of NeuAcα2,3 and NeuAcα2,6 isomers was recently described [1]. It is based on the relative intensities of the LacNAc (*m/z* 204+366) vs the NeuAc (*m/z* 274+292) generated oxonium ions (L/N) multiplied by a compensating factor for the relative number of HexNAc and NeuAc residues in the glycopeptide ($n_{(NeuAc)}/n_{(HexNAc)}$). For NeuAcα2,3 structure Ln/Nn <0.6; for NeuAcα2,6 structure Ln/Nn >0.8; and for mixed NeuAcα2,3;NeuAcα2,6 structure Ln/Nn=0.65–0.75. (**E**) MS^2 spectrum of chymotrypsin generated F.KVSNSSARGW.V (residues 639-648) of B3AT, including the same glycosite, showing an N-glycan with the Sd^a epitope and in another MS^2 spectrum (**F**) the corresponding non-Sd^a N-glycopeptide. The relative peak intensity of the Sd^a epitope was 27% for these glycopeptides. *Not related *m/z* peaks.

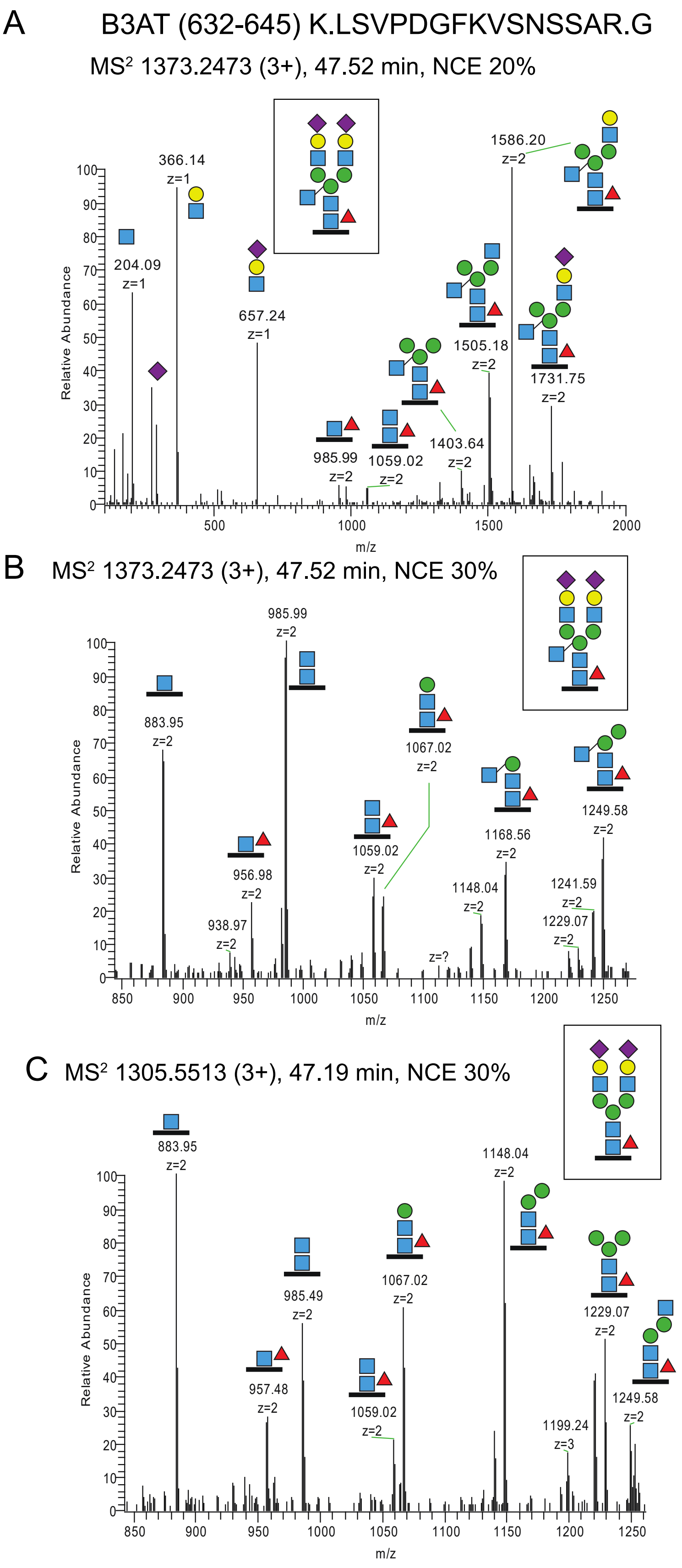

Supplementary Figure S4. Fragmentation analysis of B3AT N-glycopeptides (**A**) MS² spectrum at 20% NCE of K.LSVPDGFKVSNSSAR.G (residues 632-645) of B3AT carrying a core fucose, bisecting GlcNAc and disialo

biantennary N-glycan from an erythrocyte control individual and (**B**) a *m/z* 850-1250 expansion at 30% NCE. The lack of any ion at *m/z* 860.31 and annotated fragment ions demonstrating the presence of the bisecting GlcNAc (*m/z* 1168.56, prominent *m/z* 1586.20 and *m/z* 1505.18), compare with Fig. 8B and Supplementary Fig. S3A; and (**C**) the corresponding *m/z* 850-1250 expansion at 30% NCE for the core fucose and disialo biantennary N-glycopeptide.

```
β4GalNAc-T2 101     KPELP--SPAPGVQKLKLLPEERLRNLFSYDGIWLFPKNQCKCEAN---------KEQGG  149
                    +PELP  +P P    + +  +E++  L ++        N C CE++         K+
β4GalNAc-T1 48      RPELPDLAPEPRYAHIPVRIKEQVVGLLAW--------NNCSCESSGGGLPLPFQKQVRA  99
                                                            □ □

β4GalNAc-T2 150     YNFQDAYGQSDLPAVKARRQAEFEHFQRREGLPRPLPLLVQPNLPFGYPVHGVEVMPLHT  209
                     +   A+  ++L A  A R+ EF+ F  R   P    L+   N P  YP+ GVEV PL +
β4GalNAc-T1 100     IDLTKAFDPAELRAASATREQEFQAFLSRSQSPADQLLIAPANSPLQYPLQGVEVQPLRS  159

β4GalNAc-T2 210     VPIPGLQFEGPDA-PVYEVTLTASLGTLNTLADVPDSVVQGRGQKQLIISTSDRKLLKFI  268
                    + +PGL  +      VY+V LTASLGT +   +V    + G GQ  L + +     L
β4GalNAc-T1 160     ILVPGLSLQAASGQEVYQVNLTASLGTWDVAGEVTGVTLTGEGQADLTLVSPGLDQLNRQ  219

β4GalNAc-T2 269     LQHVTYTSTGYQHQKVDIVSLESRSSVAKFPVTIRHPVIPKLYDPG-----PERKLRNLV  323
                    LQ VTY+S  YQ    D V   +    A F + IRHP  P+LY PG      +  +  LV
β4GalNAc-T1 220     LQLVTYSSRSYQTNTADTVRFSTEGHEAAFTIRIRHPPNPRLYPPGSLPQGAQYNISALV  279

β4GalNAc-T2 324     TIATKTFLRPHKLMIMLRSIREYYPDLTVIVADDSQKPLEIKDNHVEYYTMPFGKGWFAG  383
                    TIATKTFLR  +L  ++ SIR +YP +TV++ADDS KP  +   +VE+Y MPFGKGWFAG
β4GalNAc-T1 280     TIATKTFLRYDRLRALITSIRRFYPTVTVVIADDSDKPERVSGPYVEHYLMPFGKGWFAG  339

β4GalNAc-T2 384     RNLAISQVTTKYVLWVDDDFLFNEETKIEVLVDVLEKTELDVVGGSVL---GNVFQFKLL  440
                    RNLA+SQVTTKYVLWVDDDF+F   T++E LVDVLE+T LD+VGG+V    G    ++ L
β4GalNAc-T1 340     RNLAVSQVTTKYVLWVDDDFVFTARTRLERLVDVLERTPLDLVGGAVREISGFATTYRQL  399

β4GalNAc-T2 441     LEQSEN----GACLHKRMGFFQPLDGFPSCVVTSGVVNFFLAHTERLQRVGFDPRLQRVA  496
                    L         G CL +R GF   L GFP CVVT GVVNFFLA T++++ VGFDPRL RVA
β4GalNAc-T1 400     LSVEPGAPGLGNCLRQRRGFHHELVGFPGCVVTDGVVNFFLARTDKVREVGFDPRLSRVA  459
                                ■                Δ

β4GalNAc-T2 497     HSEFFIDGLGTLLVGSCPEVIIGHQSRSPVVDSELAALEKTYNTYR-SNTLTRVQF-KLA  554
                    H EFF+DGLG+L VGSC +V++ H S+  +  +   A  +TY  YR   +L   Q  K
β4GalNAc-T1 460     HLEFFLDGLGSLRVGSCSDVVVDHASKLKLPWTSRDAGAETYARYRYPGSLDESQMAKHR  519
                                    Δ

β4GalNAc-T2 555     LHYFKNHLQC                                                    564
                    L +FK+ LQC
β4GalNAc-T1 520     LLFFKHRLQC                                                    529
                             ■
```

p.376/436Q>R (rs148441237)

p.406/466C>R (rs7224888)

p.463/523R>W (rs61743617)

Supplementary Figure S5. The β4GalNAc-T2 amino acid sequence encoded by the transcript with long exon 1 (top line) compared to β4GalNAc-T1 (bottom line). All cysteine residues of the soluble β4GalNAc-T1 are utilized to form disulfide bonds [2], here in bold and highlighted in yellow (C). Cysteine residues indicated with open squares (□) are involved in inter-protein disulfide bonds in homodimer formation and binds to the cysteines indicated with filled squares (■) in its binding partner. The bold underlined cysteines indicated with open triangles (Δ) are residues involved in intra-protein disulfide bonds. The bold blue letters indicated with a blue open circle (○) in the β4GalNAc-T2 sequence designate the amino acids affected by the mutations addressed in this paper. These amino acid changes are described in the boxes on the right with their positions in the short and long protein, e.g. p.406/466, respectively. Sequence comparison was done through BLASTP 2.13.0+, query of UniProt ID Q8NHY0 (β4GalNAc-T2) identifying subject NP_001469.1 (β4GalNAc-T1) [3,4]. Dashes in the protein sequences denote gaps. The middle line denotes amino acid match (letter) and conserved substitution (+).

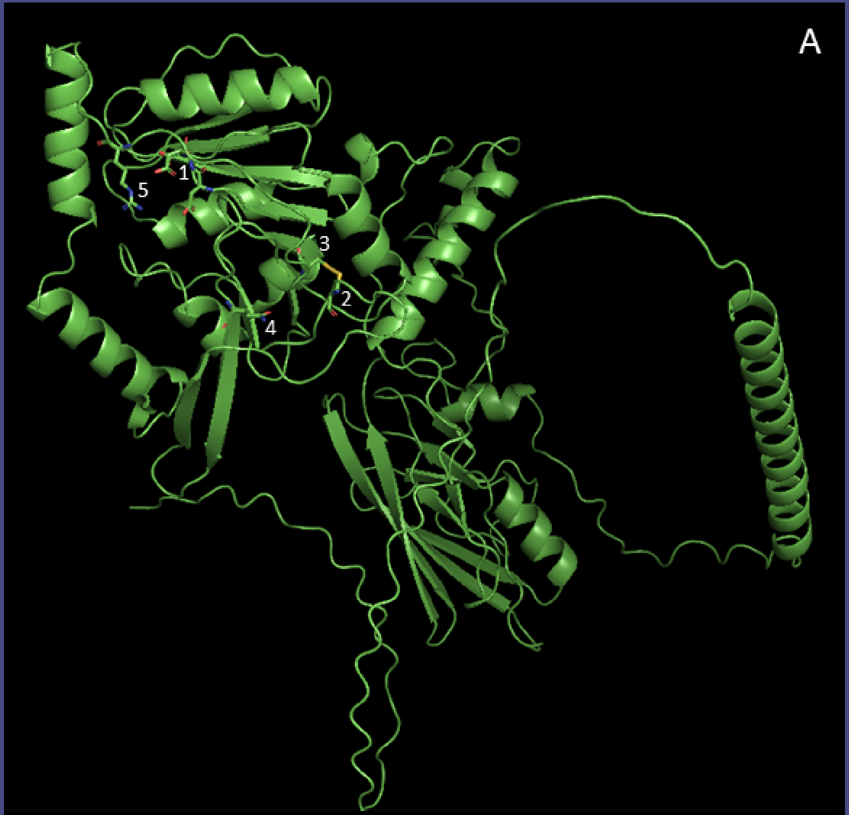

B4GALNT2 with the DDD motif[1] and disulfide bridge (yellow) between p.466Cys[2] and p.513Cys[3] highlighted, as well as the two other residues studied, p. 436Gln[4] and p. 523Arg[5] (side view).

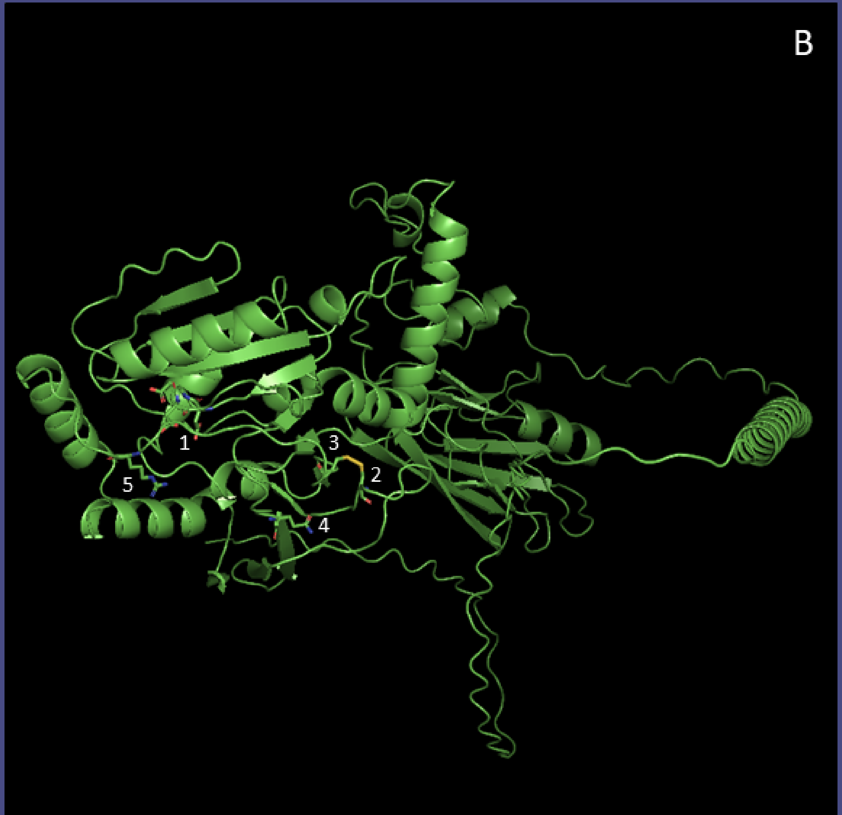

B4GALNT2 with the DDD motif[1] and disulfide bridge (yellow) between p.466Cys[2] and p.513Cys[3] highlighted, as well as the two other residues studied, p. 436Gln[4] and p. 523Arg[5] (top view).

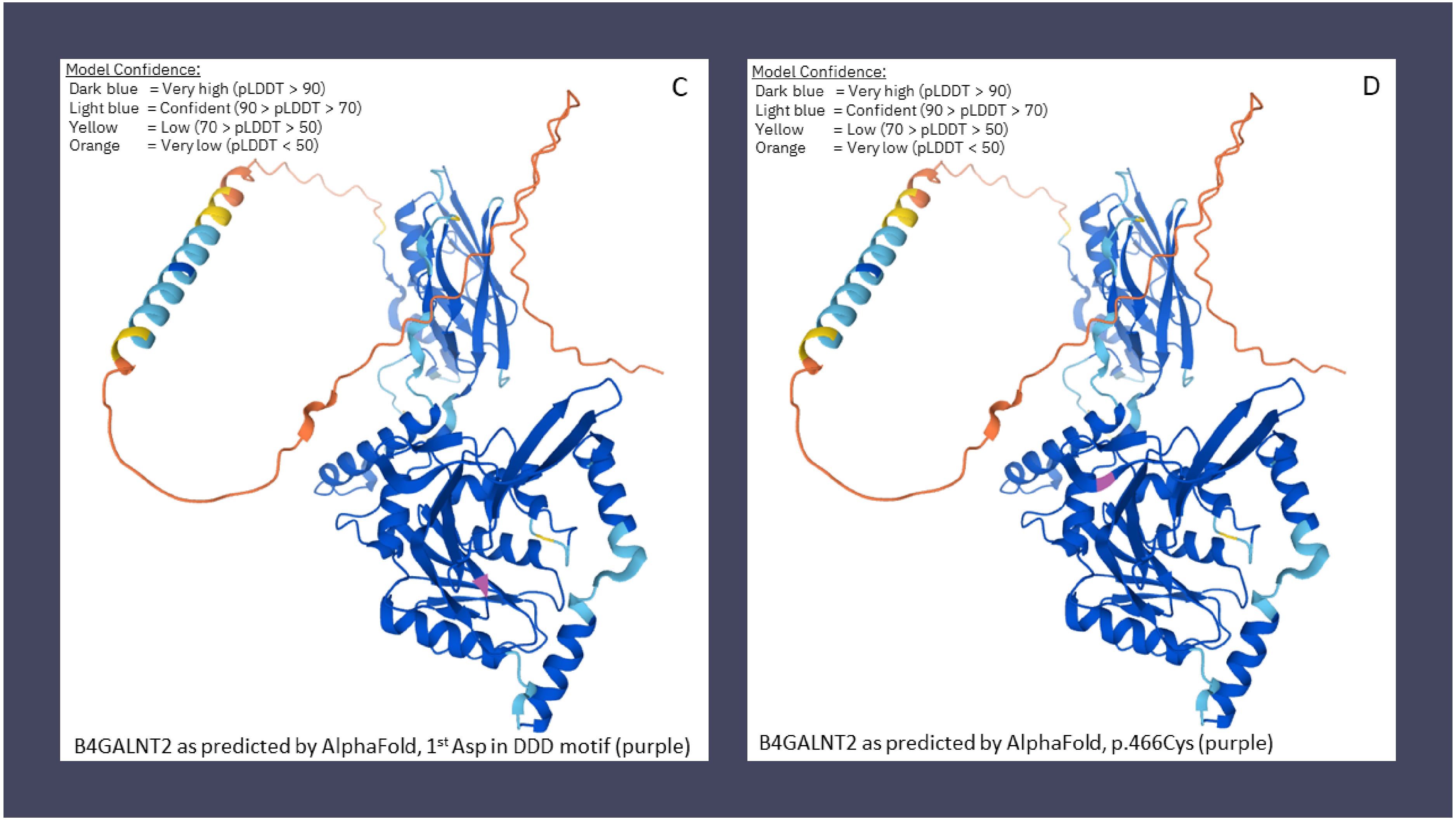

B4GALNT2 as predicted by AlphaFold, 1st Asp in DDD motif (purple)

B4GALNT2 as predicted by AlphaFold, p.466Cys (purple)

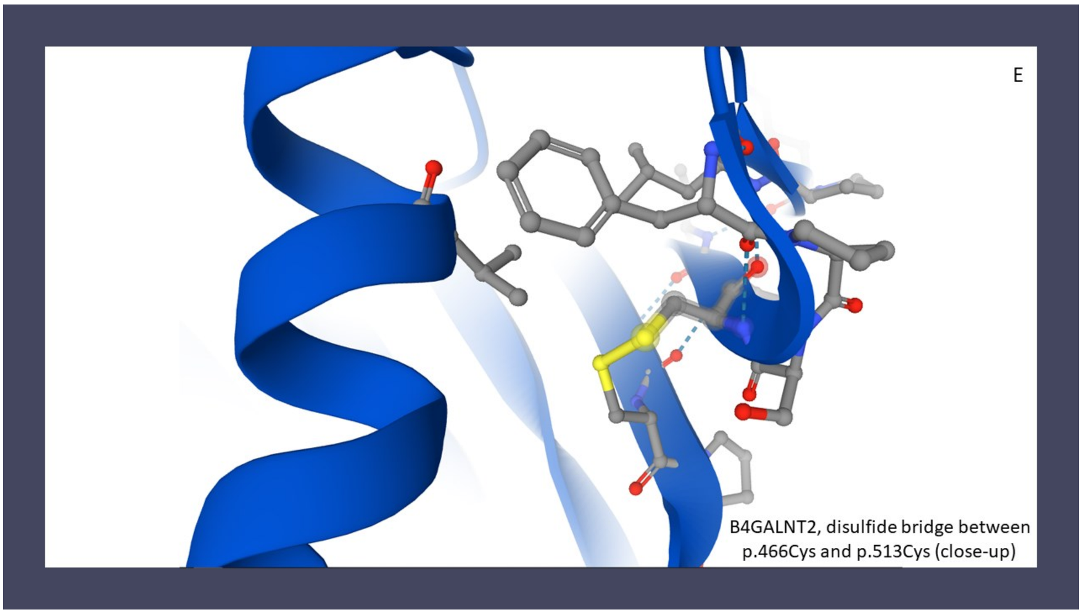
E
B4GALNT2, disulfide bridge between
p.466Cys and p.513Cys (close-up)

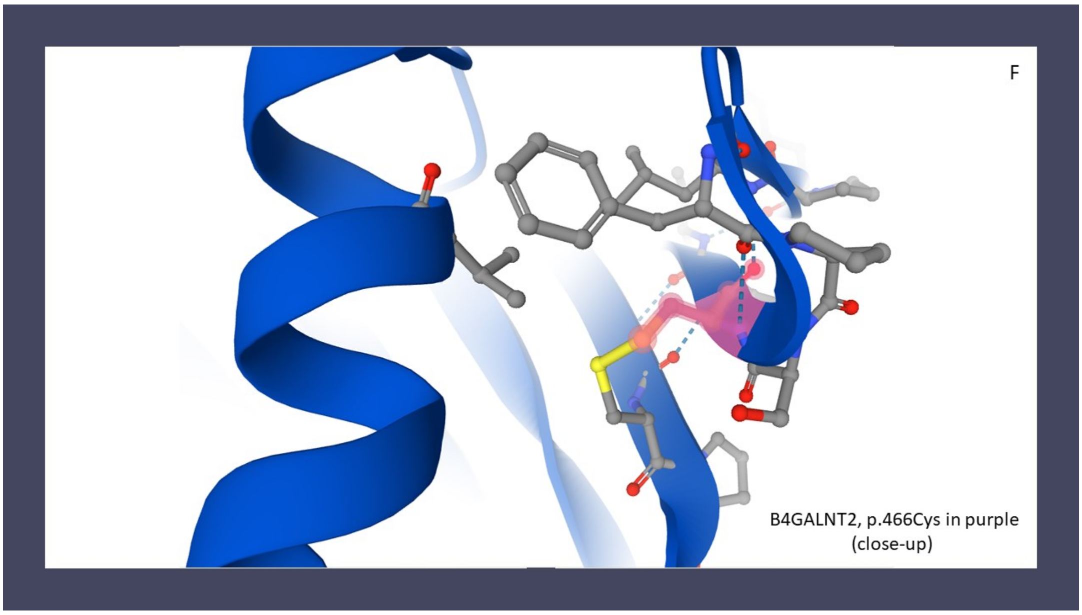
F
B4GALNT2, p.466Cys in purple
(close-up)

G

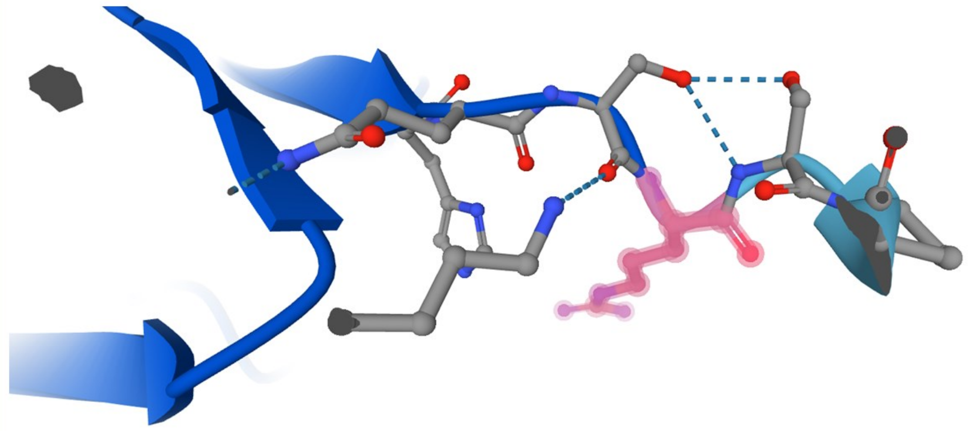

B4GALNT2 p.523Arg in purple (close-up)

H

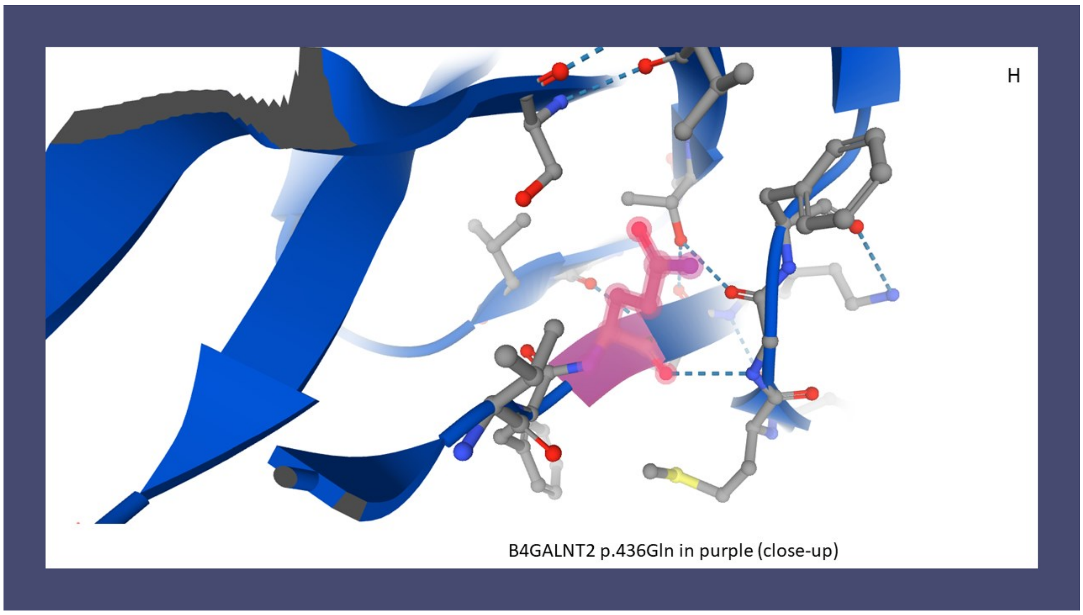

B4GALNT2 p.436Gln in purple (close-up)

I

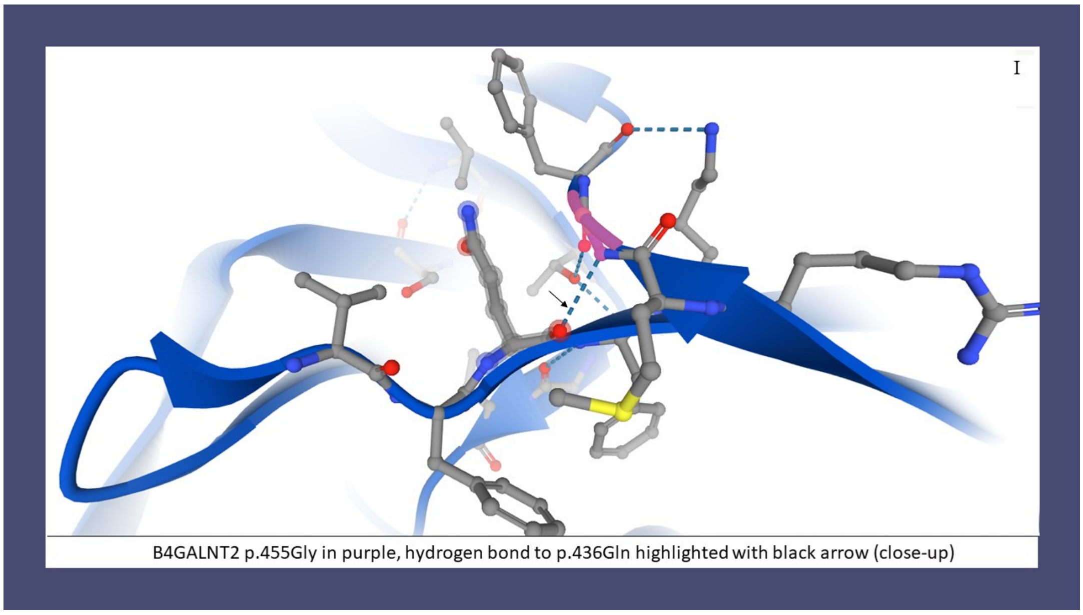

B4GALNT2 p.455Gly in purple, hydrogen bond to p.436Gln highlighted with black arrow (close-up)

Supplementary Figure S6. Three-dimensional molecular modelling of β4GalNAc-T2. Overviews of the enzyme based on Uniprot ID Q8NHY0, as predicted by AlphaFold [5] and also visualized in Pymol [6], were obtained. The relative locations of the DXD motif and the mutants studied here are highlighted specifically (S6A-D). pLDDT confidence values are shown in S6C and D. Close-ups of the relative location and molecular interactions predicted for the residues affected by alterations in Sd(a–) individuals were obtained by AlphaFold (S6E-I) as detailed in each figure.

References

1. Pett, C., Nasir, W., Sihlbom, C., Olsson, B. M., Caixeta, V., Schorlemer, M., Zahedi, R. P., Larson, G., Nilsson, J., and Westerlind, U. (2018) Effective Assignment of α2,3/α2,6-Sialic Acid Isomers by LC-MS/MS-Based Glycoproteomics. *Angew Chem Int Ed Engl* **57**, 9320-9324.
2. Li, J.; Yen, T. Y.; Allende, M. L.; Joshi, R. K.; Cai, J.; Pierce, W. M.; Jaskiewicz, E.; Darling, D. S.; Macher, B. A.; Young, W. W., Jr., Disulfide bonds of GM2 synthase homodimers. Antiparallel orientation of the catalytic domains. *J Biol Chem* 2000, 275, (52), 41476-86.
3. Altschul SF, Madden T.L., Schäffer A.A., Zhang J., Zhang Z., Miller W. and Lipman D.J. (1997) Gapped BLAST and PSI-BLAST: a new generation of protein database search programs. *Nucleic Acids Res* **25**(17), 3389-3402.
4. Altschul S.F, Wootton J.C., Gertz E.M., Agarwala R., Morgulis A., Schäffer A.A. and Yu Y-K. (2005) Protein database searches using compositionally adjusted substitution matrices. *FEBS J* **272**(20), 5101-5109.
5. Varadi, M.; Anyango, S.; Deshpande, M.; Nair, S.; Natassia, C.; Yordanova, G.; Yuan, D.; Stroe, O.; Wood, G.; Laydon, A.; Žídek, A.; Green, T.; Tunyasuvunakool, K.; Petersen, S.; Jumper, J.; Clancy, E.; Green, R.; Vora, A.; Lutfi, M.; Figurnov, M.; Cowie, A.; Hobbs, N.; Kohli, P.; Kleywegt, G.; Birney, E.; Hassabis, D.; Velankar, S., AlphaFold Protein Structure Database: massively expanding the structural coverage of protein-sequence space with high-accuracy models. *Nucleic Acids Res* 2022, 50, (D1), D439-d444.
6. Schrödinger, L. L. C. The PyMOL Molecular Graphics System, Version 2.3.4.
