# Supplementary material for: Glycoproteomic and Phenotypic Elucidation of B4GALNT2 Expression Variants in the SID Histo-Blood Group System
Source: Int J Mol Sci. 2022 Apr 1;23(7):3936. doi: 10.3390/ijms23073936 (PMC8999409; doi:10.3390/ijms23073936)

Supplementary Materials

Glycoproteomic and phenotypic elucidation of *B4GALNT2* expression variants in the SID histo-blood group system

Linn Stenfelt[1]*, Jonas Nilsson[2]**, Åsa Hellberg[3], Yew Wah Liew[4], Jenny Morrison[4], Göran Larson[5,6], Martin L Olsson[1,3]**

[1] Division of Hematology and Transfusion Medicine, Department of Laboratory Medicine, Lund University, Lund, Sweden
[2] Proteomics Core Facility, Sahlgrenska Academy at the University of Gothenburg, Gothenburg, Sweden
[3] Department of Clinical Immunology and Transfusion Medicine, Office for Medical Services, Region Skåne, Sweden
[4] Red Cell Reference Laboratory, Clinical Services and Research, Australian Red Cross Lifeblood, Kelvin Grove, Australia
[5]Laboratory of Clinical Chemistry, Sahlgrenska University Hospital, Gothenburg, Sweden
[6]Department of Laboratory Medicine, Institute of Biomedicine, Sahlgrenska Academy at the University of Gothenburg, Gothenburg, Sweden
*Currently at: Section for Protein Chemistry and Enzyme Technology, Department of Biotechnology and Biomedicine, Technical University of Denmark, Copenhagen, 2800 Kgs. Lyngby, Denmark

****Corresponding authors**:

Prof. Martin L Olsson, M.D., Ph.D.
E-mail: Martin_L.Olsson@med.lu.se

Jonas Nilsson, Ph.D.
E-mail: Jonas.GM.Nilsson@gu.se

Supplementary Spectra Collections 1 and 2

Spectrum collection 1. Glycosidic fragmentation analysis of Sd ^{a} epitope glycopeptides of HEK293 cells transfected with *B4GALNT2* constructs. A normalized collision energy (NCE) of 20% was used. The panel order is according to Fig. 5. Spectra of TM9S3 and TFR1 (O-glycopeptide) are provided in Fig. 4. Information regarding MS files, scan numbers, precuror masses and charges are provided in Table S1 and S2. The Byonic annotated spectra at NCE 30%/40% are in **Spectrum collection 2**.

a. AT1B3

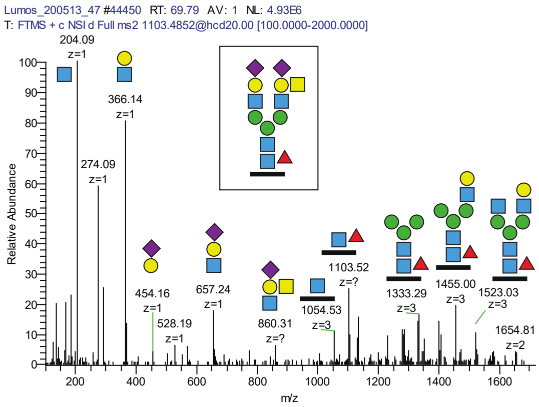

b. AT1B3

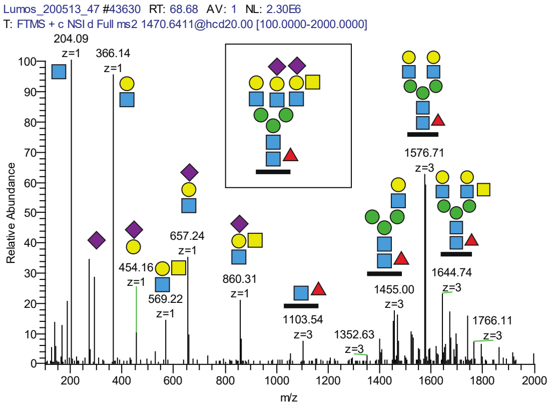

c. CD276

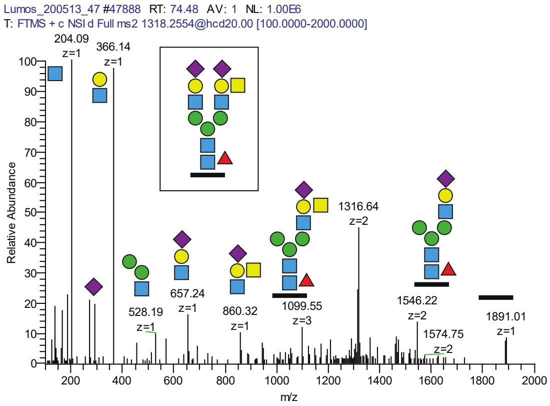

d. PTPRF

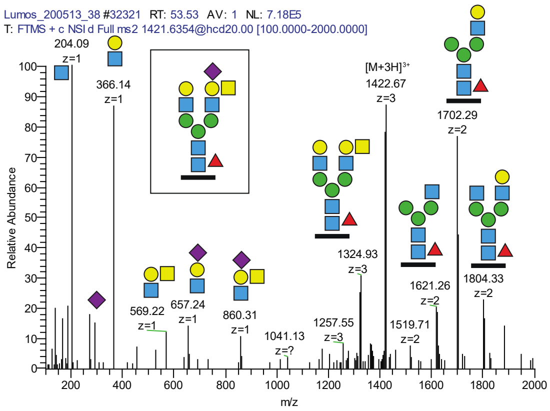

e. TFR1

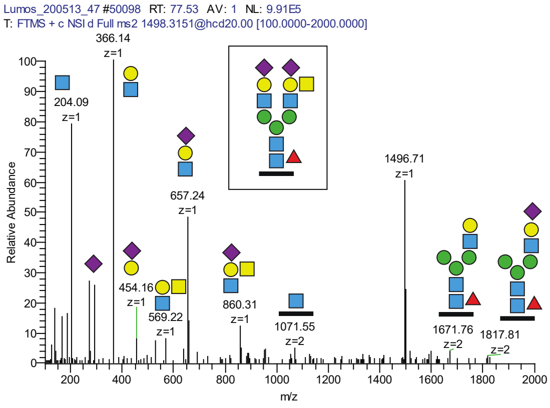

f. TFR1

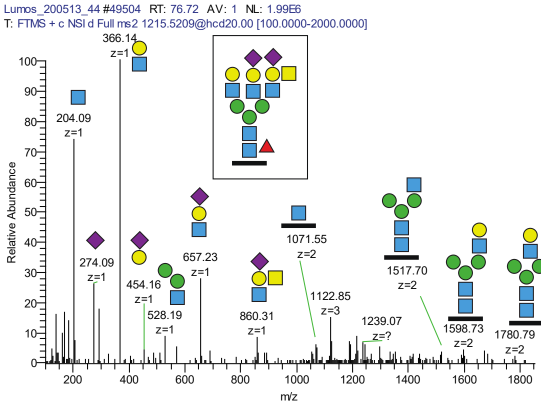

g. 4F2

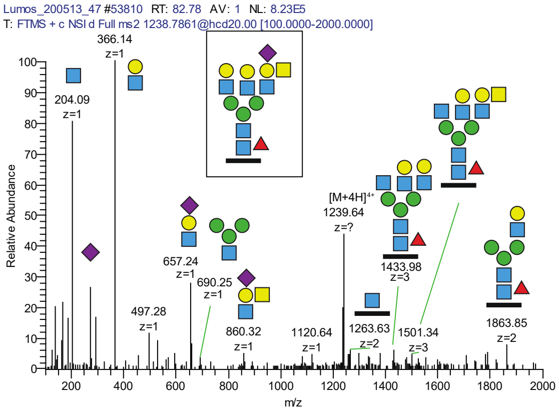

h. CD63

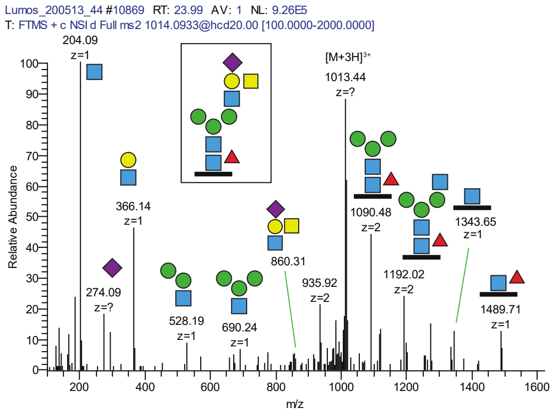

i. LAMP1

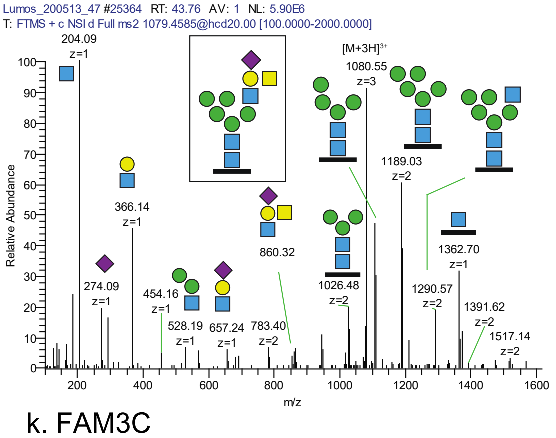

j. GPC4

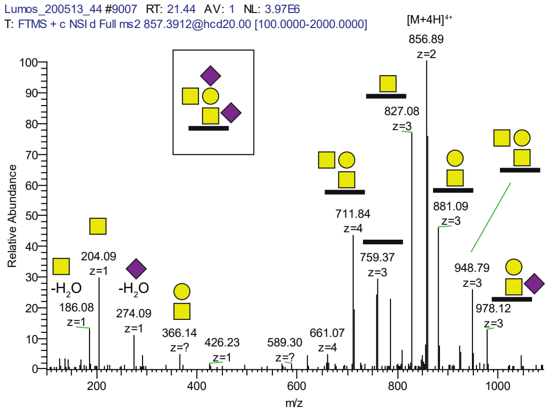

k. FAM3C

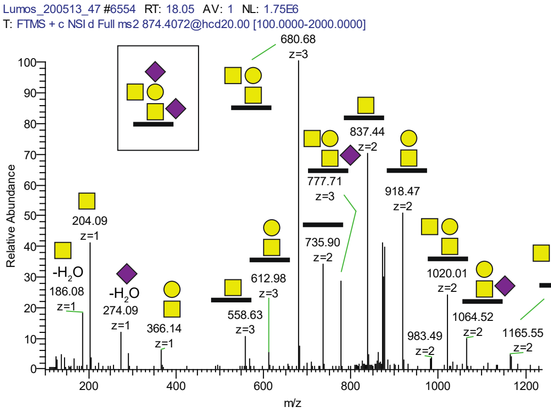

Index of Spectrum collection 2. Part 1. Glycopeptides from HEK293 cells.

Spectrum#	Sample	Sequence	Glycan composition	Protein
N-glycopeptides HEK293				
1	Mock	R.IVDVNLTSEGK.V	HexNAc(4)Hex(5)NeuAc(2)	Q9HD45|TM9S3
2	Mock	K.FLKPYTLEEQKNLTVCPDGALFEQK.G	HexNAc(4)Hex(5)Fuc(1)NeuAc(2)	P54709|AT1B3
3	Mock	K.FLKPYTLEEQKNLTVCPDGALFEQK.G	HexNAc(5)Hex(6)Fuc(1)NeuAc(3)	P54709|AT1B3
4	Mock	R.TALFPDLLAQGNASLR.L	HexNAc(4)Hex(5)Fuc(1)NeuAc(1)	Q5ZPR3|CD276
5	Mock	R.KVEVEPLNSTAVHVYWK.L	HexNAc(4)Hex(5)Fuc(1)NeuAc(1)	P10586|PTPRF
6	Mock	K.KDFEDLYTPVNGSIVIVR.A	HexNAc(4)Hex(5)Fuc(1)NeuAc(1)	P02786|TFR1
7	Mock	K.KDFEDLYTPVNGSIVIVR.A	HexNAc(5)Hex(6)Fuc(1)NeuAc(2)	P02786|TFR1
8	Mock	R.DIENLKDASSFLAEWQNITK.G	HexNAc(5)Hex(6)Fuc(1)NeuAc(1)	P08195|4F2
9	Mock	K.NNHTASILDR.M	HexNAc(3)Hex(4)Fuc(1)NeuAc(1)	P08962|CD63
10	Mock	K.NNHTASILDR.M	HexNAc(2)Hex(3)Fuc(1)	P08962|CD63
11	Mock	R.GHTLTLNFTR.N	HexNAc(3)Hex(6)NeuAc(1)	P11279|LAMP1
12	B4GALNT2 WT	R.IVDVNLTSEGK.V	HexNAc(6)Hex(5)NeuAc(2)	Q9HD45|TM9S3
13	B4GALNT2 WT	K.FLKPYTLEEQKNLTVCPDGALFEQK.G	HexNAc(5)Hex(5)Fuc(1)NeuAc(2)	P54709|AT1B3
14	B4GALNT2 WT	K.FLKPYTLEEQKNLTVCPDGALFEQK.G	HexNAc(4)Hex(5)Fuc(1)NeuAc(2)	P54709|AT1B3
15	B4GALNT2 WT	K.FLKPYTLEEQKNLTVCPDGALFEQK.G	HexNAc(6)Hex(6)Fuc(1)NeuAc(2)	P54709|AT1B3
16	B4GALNT2 WT	K.FLKPYTLEEQKNLTVCPDGALFEQK.G	HexNAc(5)Hex(6)Fuc(1)NeuAc(2)	P54709|AT1B3
17	B4GALNT2 WT	R.TALFPDLLAQGNASLR.L	HexNAc(5)Hex(5)Fuc(1)NeuAc(1)	Q5ZPR3|CD276
18	B4GALNT2 WT	R.TALFPDLLAQGNASLR.L	HexNAc(4)Hex(5)Fuc(1)NeuAc(1)	Q5ZPR3|CD276
19	B4GALNT2 WT	R.KVEVEPLNSTAVHVYWK.L	HexNAc(4)Hex(5)Fuc(1)NeuAc(1)	P10586|PTPRF
20	B4GALNT2 WT	K.DFEDLYTPVNGSIVIVR.A	HexNAc(5)Hex(5)Fuc(1)NeuAc(2)	P02786|TFR1
21	B4GALNT2 WT	K.DFEDLYTPVNGSIVIVR.A	HexNAc(4)Hex(5)Fuc(1)NeuAc(1)	P02786|TFR1
22	B4GALNT2 WT	K.DFEDLYTPVNGSIVIVR.A	HexNAc(6)Hex(6)Fuc(1)NeuAc(2)	P02786|TFR1
23	B4GALNT2 WT	K.KDFEDLYTPVNGSIVIVR.A	HexNAc(5)Hex(6)Fuc(1)NeuAc(3)	P02786|TFR1
24	B4GALNT2 WT	R.DIENLKDASSFLAEWQNITK.G	HexNAc(6)Hex(6)Fuc(1)NeuAc(1)	P08195|4F2
25	B4GALNT2 WT	R.GHTLTLNFTR.N	HexNAc(4)Hex(6)NeuAc(1)	P11279|LAMP1
26	B4GALNT2 WT	R.GHTLTLNFTR.N	HexNAc(3)Hex(6)NeuAc(1)	P11279|LAMP1
27	rs7224888	R.IVDVNLTSEGK.V	HexNAc(4)Hex(5)NeuAc(2)	Q9HD45|TM9S3
28	rs7224888	K.FLKPYTLEEQKNLTVCPDGALFEQK.G	HexNAc(4)Hex(5)Fuc(1)NeuAc(2)	P54709|AT1B3
29	rs7224888	K.FLKPYTLEEQKNLTVCPDGALFEQK.G	HexNAc(5)Hex(6)Fuc(1)NeuAc(2)	P54709|AT1B3
30	rs7224888	R.TALFPDLLAQGNASLR.L	HexNAc(4)Hex(5)Fuc(1)NeuAc(1)	Q5ZPR3|CD276
31	rs7224888	R.KVEVEPLNSTAVHVYWK.L	HexNAc(4)Hex(5)Fuc(1)NeuAc(1)	P10586|PTPRF
32	rs7224888	K.DFEDLYTPVNGSIVIVR.A	HexNAc(4)Hex(5)Fuc(1)NeuAc(1)	P02786|TFR1
33	rs7224888	K.KDFEDLYTPVNGSIVIVR.A	HexNAc(5)Hex(6)Fuc(1)NeuAc(2)	P02786|TFR1
34	rs7224888	R.DIENLKDASSFLAEWQNITK.G	HexNAc(5)Hex(6)Fuc(1)NeuAc(1)	P08195|4F2
35	rs7224888	K.NNHTASILDR.M	HexNAc(3)Hex(4)Fuc(1)NeuAc(1)	P08962|CD63
36	rs7224888	R.GHTLTLNFTR.N	HexNAc(3)Hex(6)NeuAc(1)	P11279|LAMP1
37	rs148441237	R.IVDVNLTSEGK.V	HexNAc(6)Hex(5)NeuAc(2)	Q9HD45|TM9S3
38	rs148441237	K.FLKPYTLEEQKNLTVCPDGALFEQK.G	HexNAc(5)Hex(5)Fuc(1)NeuAc(2)	P54709|AT1B3
39	rs148441237	K.FLKPYTLEEQKNLTVCPDGALFEQK.G	HexNAc(4)Hex(5)Fuc(1)NeuAc(1)	P54709|AT1B3
40	rs148441237	K.FLKPYTLEEQKNLTVCPDGALFEQK.G	HexNAc(6)Hex(6)Fuc(1)NeuAc(2)	P54709|AT1B3

41	rs148441237	K.FLKPYTLEEQKNLTVCPDGALFEQK.G	HexNAc(5)Hex(6)Fuc(1)NeuAc(1)	P54709|AT1B3
42	rs148441237	R.TALFPDLLAQGNASLR.L	HexNAc(5)Hex(5)NeuAc(1)	Q5ZPR3|CD276
43	rs148441237	R.TALFPDLLAQGNASLR.L	HexNAc(4)Hex(5)NeuAc(1)	Q5ZPR3|CD276
44	rs148441237	R.KVEVEPLNSTAVHVYWK.L	HexNAc(5)Hex(5)Fuc(1)NeuAc(1)	P10586|PTPRF
45	rs148441237	R.KVEVEPLNSTAVHVYWK.L	HexNAc(4)Hex(5)Fuc(1)NeuAc(1)	P10586|PTPRF
46	rs148441237	K.DFEDLYTPVNGSIVIVR.A	HexNAc(5)Hex(5)Fuc(1)NeuAc(2)	P02786|TFR1
47	rs148441237	K.DFEDLYTPVNGSIVIVR.A	HexNAc(6)Hex(6)Fuc(1)NeuAc(2)	P02786|TFR1
48	rs148441237	K.KDFEDLYTPVNGSIVIVR.A	HexNAc(5)Hex(6)Fuc(1)NeuAc(1)	P02786|TFR1
49	rs148441237	K.NNHTASILDR.M	HexNAc(4)Hex(4)Fuc(1)NeuAc(1)	P08962|CD63
50	rs148441237	K.NNHTASILDR.M	HexNAc(3)Hex(4)Fuc(1)NeuAc(1)	P08962|CD63
51	rs148441237	R.GHTLTLNFTR.N	HexNAc(4)Hex(6)NeuAc(1)	P11279|LAMP1
52	rs148441237	R.GHTLTLNFTR.N	HexNAc(3)Hex(6)NeuAc(1)	P11279|LAMP1
53	rs61743617	R.IVDVNLTSEGK.V	HexNAc(4)Hex(5)NeuAc(2)	Q9HD45|TM9S3
54	rs61743617	K.FLKPYTLEEQKNLTVCPDGALFEQK.G	HexNAc(5)Hex(5)Fuc(1)NeuAc(1)	P54709|AT1B3
55	rs61743617	K.FLKPYTLEEQKNLTVCPDGALFEQK.G	HexNAc(4)Hex(5)Fuc(1)NeuAc(1)	P54709|AT1B3
56	rs61743617	K.FLKPYTLEEQKNLTVCPDGALFEQK.G	HexNAc(6)Hex(6)Fuc(1)NeuAc(2)	P54709|AT1B3
57	rs61743617	K.FLKPYTLEEQKNLTVCPDGALFEQK.G	HexNAc(5)Hex(6)Fuc(1)NeuAc(1)	P54709|AT1B3
58	rs61743617	R.TALFPDLLAQGNASLR.L	HexNAc(5)Hex(5)Fuc(1)NeuAc(1)	Q5ZPR3|CD276
59	rs61743617	R.TALFPDLLAQGNASLR.L	HexNAc(4)Hex(5)Fuc(1)NeuAc(1)	Q5ZPR3|CD276
60	rs61743617	R.KVEVEPLNSTAVHVYWK.L	HexNAc(5)Hex(5)Fuc(1)NeuAc(1)	P10586|PTPRF
61	rs61743617	R.KVEVEPLNSTAVHVYWK.L	HexNAc(4)Hex(5)Fuc(1)NeuAc(1)	P10586|PTPRF
62	rs61743617	K.KDFEDLYTPVNGSIVIVR.A	HexNAc(6)Hex(6)Fuc(1)NeuAc(2)	P02786|TFR1
63	rs61743617	K.KDFEDLYTPVNGSIVIVR.A	HexNAc(5)Hex(6)Fuc(1)NeuAc(1)	P02786|TFR1
64	rs61743617	K.NNHTASILDR.M	HexNAc(4)Hex(4)Fuc(1)NeuAc(1)	P08962|CD63
65	rs61743617	R.GHTLTLNFTR.N	HexNAc(4)Hex(6)NeuAc(1)	P11279|LAMP1
66	rs61743617	R.GHTLTLNFTR.N	HexNAc(3)Hex(6)NeuAc(1)	P11279|LAMP1
O-glycopeptides HEK 293				
67	Mock	R.FRPHHPEERPTTAAGTSLDR.L	HexNAc(1)Hex(1)NeuAc(2)	O75487|GPC4
68	Mock	R.SALDTAARSTKPPR.Y	HexNAc(1)Hex(1)NeuAc(2)	Q92520|FAM3C
69	Mock	R.LAGTESPVREEPGEDFPAAR.R	HexNAc(1)Hex(1)NeuAc(2)	P02786|TFR1
70	B4 GALNT2 wt	R.FRPHHPEERPTTAAGTSLDR.L	HexNAc(2)Hex(1)NeuAc(2)	O75487|GPC4
71	B4 GALNT2 wt	R.SALDTAARSTKPPR.Y	HexNAc(2)Hex(1)NeuAc(2)	Q92520|FAM3C
72	B4 GALNT2 wt	R.SALDTAARSTKPPR.Y	HexNAc(1)Hex(1)NeuAc(2)	Q92520|FAM3C
73	B4 GALNT2 wt	R.LAGTESPVREEPGEDFPAAR.R	HexNAc(2)Hex(1)NeuAc(2)	P02786|TFR1
74	rs7224888	R.FRPHHPEERPTTAAGTSLDR.L	HexNAc(1)Hex(1)NeuAc(2)	O75487|GPC4
75	rs7224888	R.SALDTAARSTKPPR.Y	HexNAc(1)Hex(1)NeuAc(2)	Q92520|FAM3C
76	rs7224888	R.LAGTESPVREEPGEDFPAAR.R	HexNAc(1)Hex(1)NeuAc(2)	P02786|TFR1
77	rs148441237	R.FRPHHPEERPTTAAGTSLDR.L	HexNAc(2)Hex(1)NeuAc(2)	O75487|GPC4
78	rs148441237	R.SALDTAARSTKPPR.Y	HexNAc(2)Hex(1)NeuAc(2)	Q92520|FAM3C
79	rs148441237	R.LAGTESPVREEPGEDFPAAR.R	HexNAc(2)Hex(1)NeuAc(2)	P02786|TFR1
80	rs61743617	R.FRPHHPEERPTTAAGTSLDR.L	HexNAc(2)Hex(1)NeuAc(2)	O75487|GPC4
81	rs61743617	R.FRPHHPEERPTTAAGTSLDR.L	HexNAc(1)Hex(1)NeuAc(2)	O75487|GPC4
82	rs61743617	R.SALDTAARSTKPPR.Y	HexNAc(2)Hex(1)NeuAc(2)	Q92520|FAM3C

83	rs61743617	R.LAGTESPVREEPGEDFPAAR.R	HexNAc(2)Hex(1)NeuAc(2)	P02786|TFR1
84	rs61743617	R.LAGTESPVREEPGEDFPAAR.R	HexNAc(1)Hex(1)NeuAc(2)	P02786|TFR1

Index of Spectrum collection 2. Part 2. Glycopeptides from erythrocytes.

Spectrum#	Sample	Sequence	Glycan composition	Protein
N-glycopeptides erythrocytes				
1	Cad-a	K.LSVPDGFKVSNSSAR.G	HexNAc(5)Hex(5)Fuc(1)NeuAc(2)	P02730|B3AT
2	Cad-a	K.LSVPDGFKVSNSSAR.G	HexNAc(4)Hex(5)Fuc(1)NeuAc(2)	P02730|B3AT
3	Control	K.LSVPDGFKVSNSSAR.G	HexNAc(4)Hex(5)Fuc(1)NeuAc(2)	P02730|B3AT
4	Cad-a (chymotrypsin)	F.KVSNSSARGW.V	HexNAc(5)Hex(5)Fuc(1)NeuAc(2)	P02730|B3AT
5	Cad-a (chymotrypsin)	F.KVSNSSARGW.V	HexNAc(4)Hex(5)Fuc(1)NeuAc(2)	P02730|B3AT
O-glycopeptides erythrocytes				
6	Cad-a	R.DTYAATPR.A	HexNAc(2)Hex(1)NeuAc(2)	P02724|GLPA
7	Cad-a	R.DTYAATPR.A	HexNAc(1)Hex(1)NeuAc(2)	P02724|GLPA
8	Cad-a	R.AHEVSEISVRTVYPPEEETGER.V	HexNAc(4)Hex(2)NeuAc(4)	P02724|GLPA
9	Cad-a	R.AHEVSEISVRTVYPPEEETGER.V	HexNAc(3)Hex(2)NeuAc(4)	P02724|GLPA
10	Cad-a	R.AHEVSEISVRTVYPPEEETGER.V	HexNAc(2)Hex(2)NeuAc(4)	P02724|GLPA
11	Cad-a	K.DAQASAAPAAPLPER.N	HexNAc(2)Hex(1)NeuAc(2)	Q99808|S29A1
12	Cad-a	K.DAQASAAPAAPLPER.N	HexNAc(1)Hex(1)NeuAc(2)	Q99808|S29A1
13	Cad-b	R.DTYAATPR.A	HexNAc(1)Hex(1)NeuAc(2)	P02724|GLPA
14	Cad-b	R.AHEVSEISVRTVYPPEEETGER.V	HexNAc(2)Hex(2)NeuAc(4)	P02724|GLPA
15	Cad-b	K.DAQASAAPAAPLPER.N	HexNAc(1)Hex(1)NeuAc(2)	Q99808|S29A1
16	Cad-b (pronase)	V.RTVYPPEEE.T	HexNAc(2)Hex(1)NeuAc(2)	P02724|GLPA
17	Cad-b (pronase)	V.RTVYPPEEE.T	HexNAc(1)Hex(1)NeuAc(2)	P02724|GLPA
18	Control (pronase)	V.RTVYPPEEE.T	HexNAc(1)Hex(1)NeuAc(2)	P02724|GLPA
19	Control	R.DTYAATPR.A	HexNAc(1)Hex(1)NeuAc(2)	P02724|GLPA
20	Control	R.AHEVSEISVRTVYPPEEETGER.V	HexNAc(2)Hex(2)NeuAc(4)	P02724|GLPA
21	Control	K.DAQASAAPAAPLPER.N	HexNAc(1)Hex(1)NeuAc(2)	Q99808|S29A1

Spectrum collection 2, Part 1. Byonic annotated MS^2 spectra of glycopeptides from HEK293 cells transfected with B4GALNT2 constructs. Information regarding MS files, scan numbers, precuror masses and charges are provided in Supplementary Table S1 and S2.

The spectra of (9) does not include significant b-/y-ions, and (10) is alternatively provided showing the same peptide+HexNAc ion.

1. TM9S3, Mock transfected

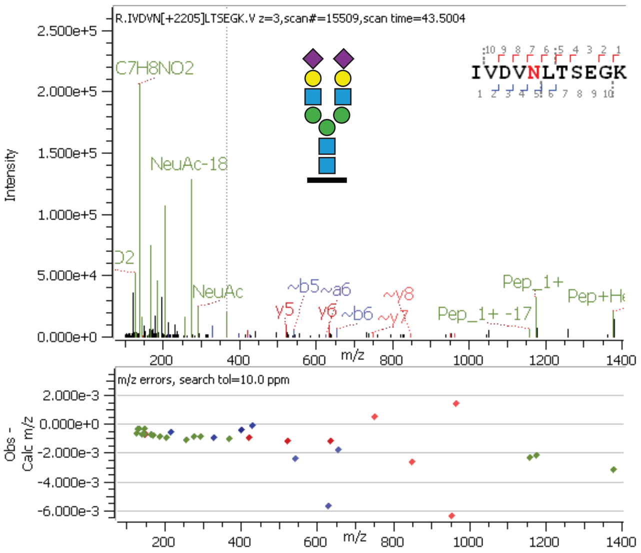

2. AT1B3, Mock transfected

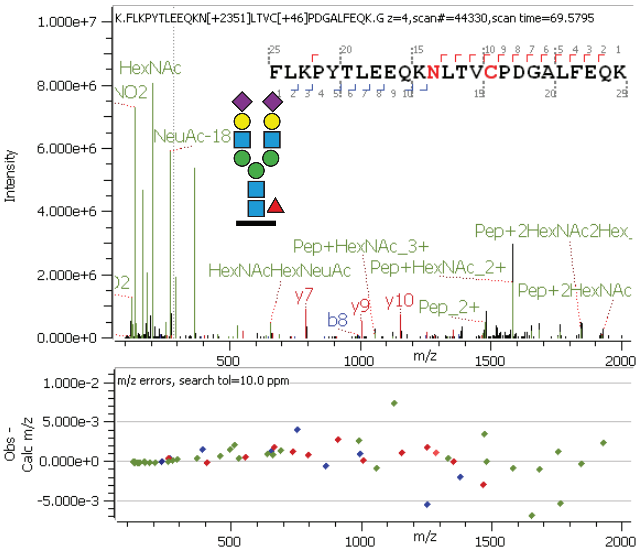

3. AT1B3, Mock transfected

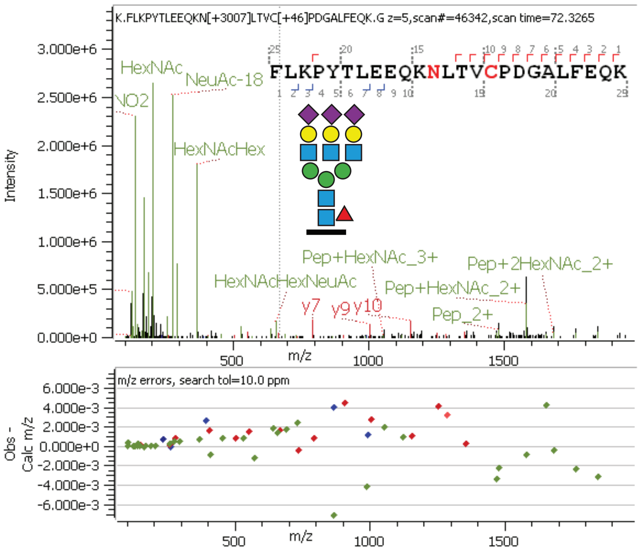

4. CD276, Mock transfected

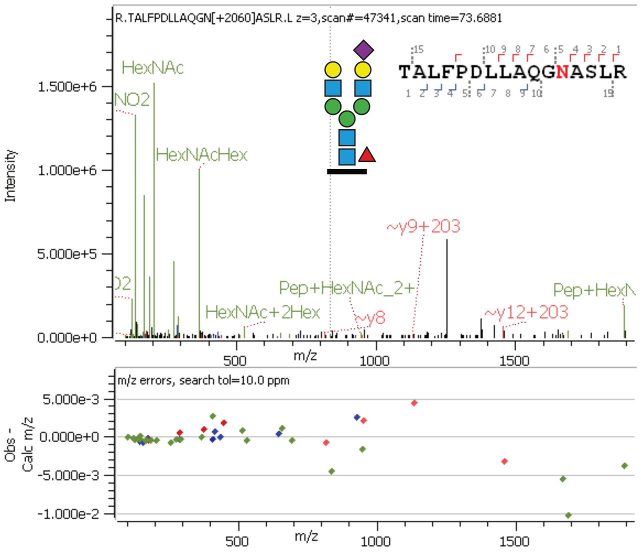

5. PTPRF, Mock transfected

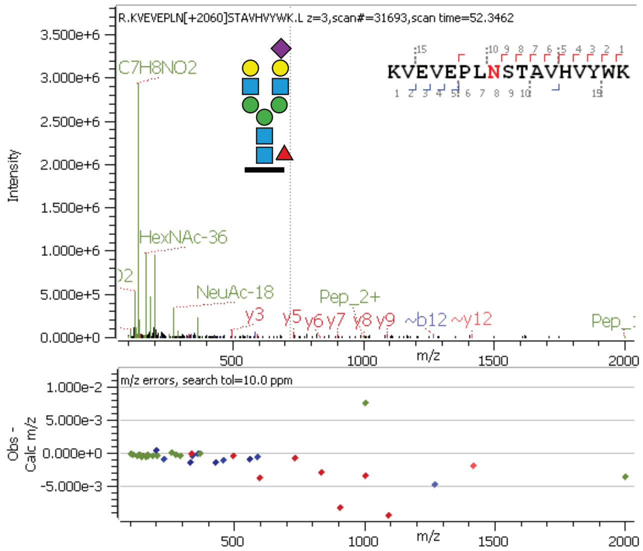

6. TFR1, Mock transfected

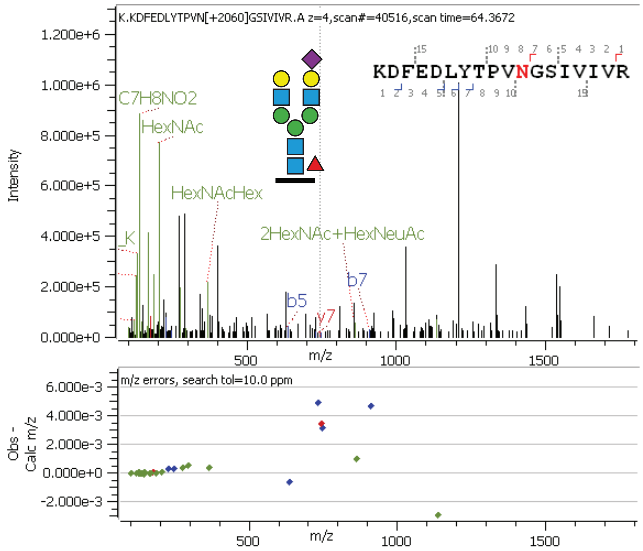

7. TFR1, Mock transfected

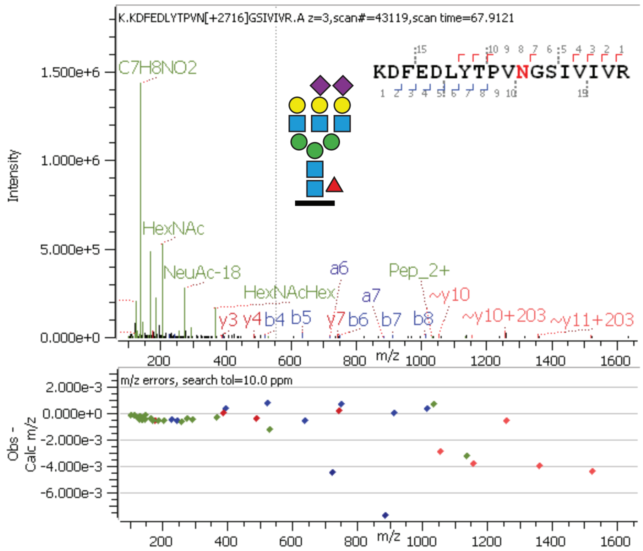

8. 4F2, Mock transfected

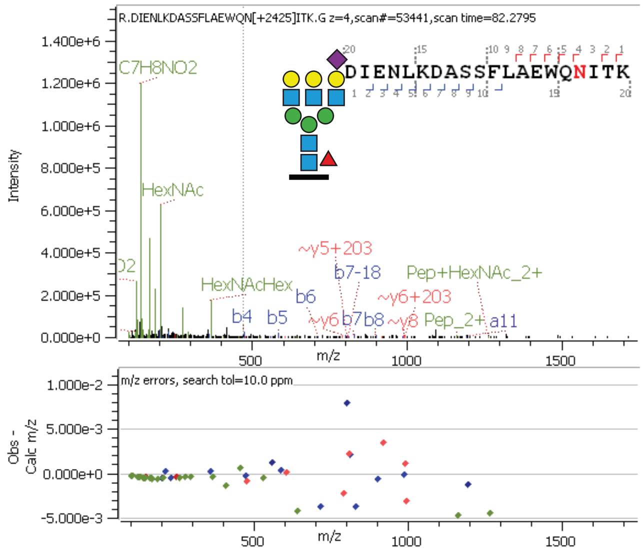

9. CD63, Mock transfected

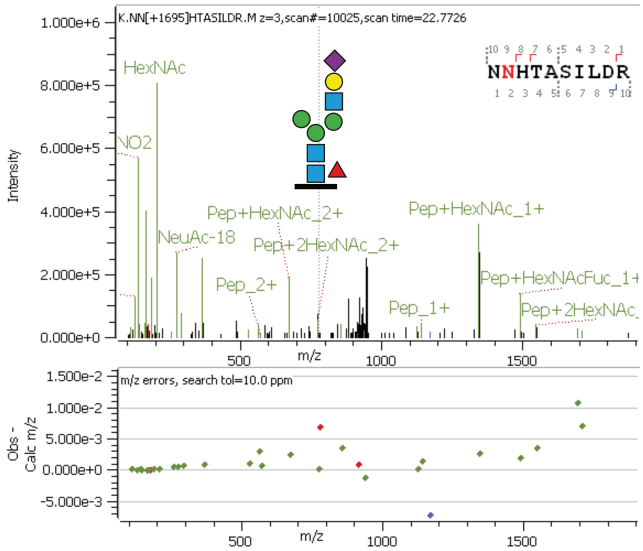

10. CD63, Mock transfected

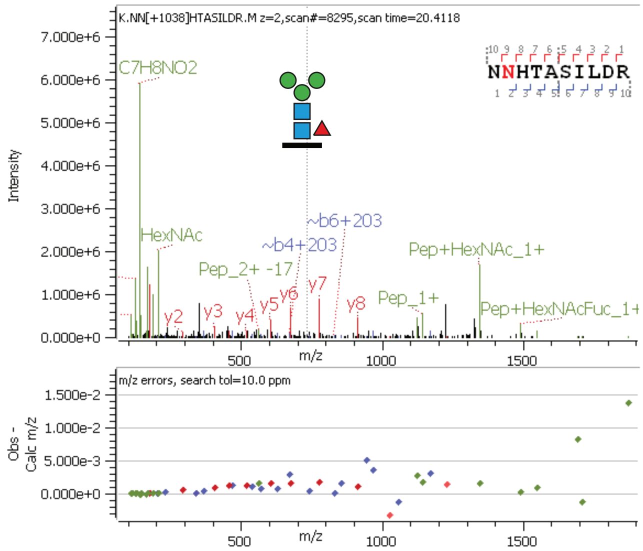

11. LAMP1, Mock transfected

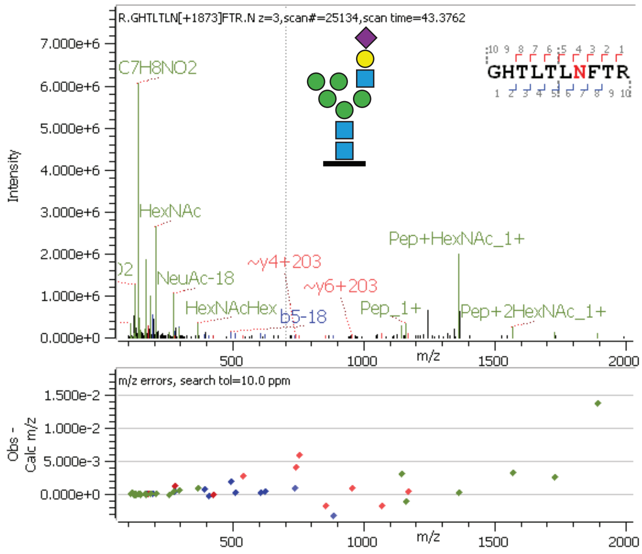

12. TM9S3, B4GALNT2 WT

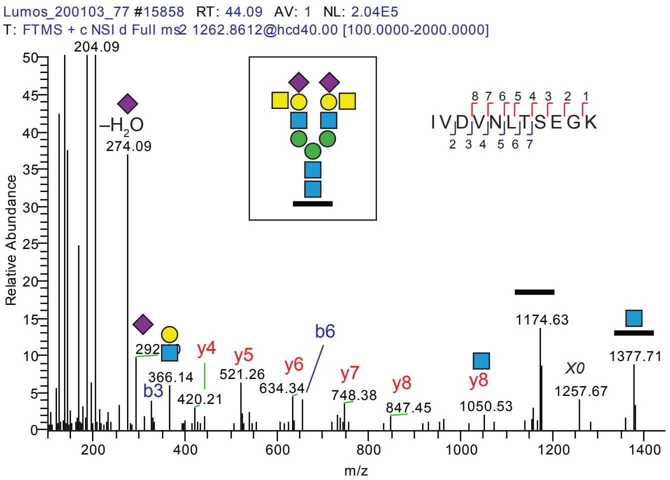

13. AT1B3, B4GALNT2 WT

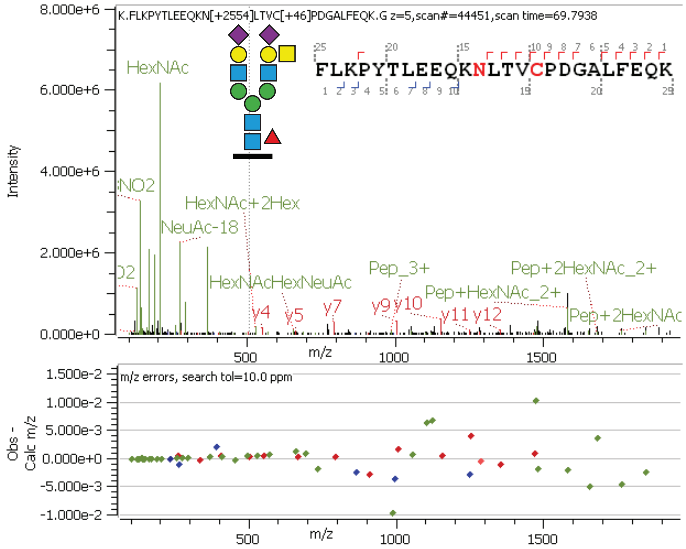

14. AT1B3, B4GALNT2 WT

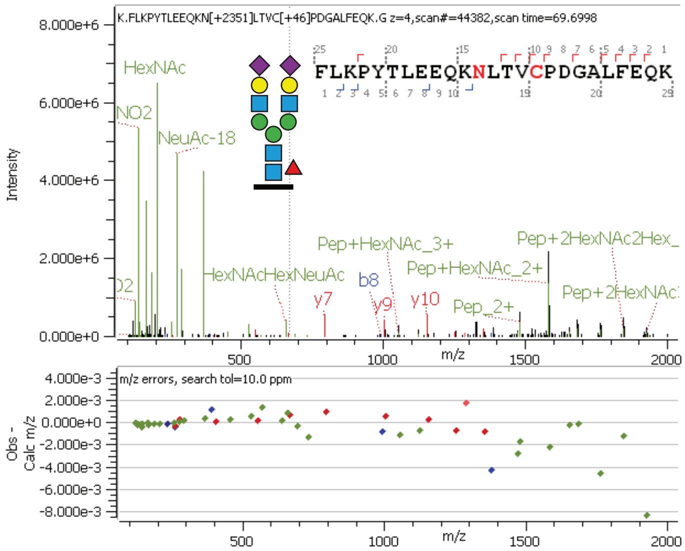

15. AT1B3, B4GALNT2 WT

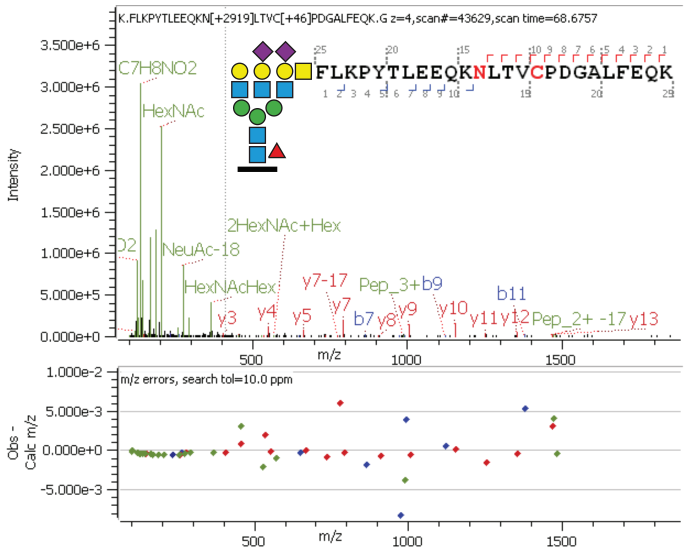

16. AT1B3, B4GALNT2 WT

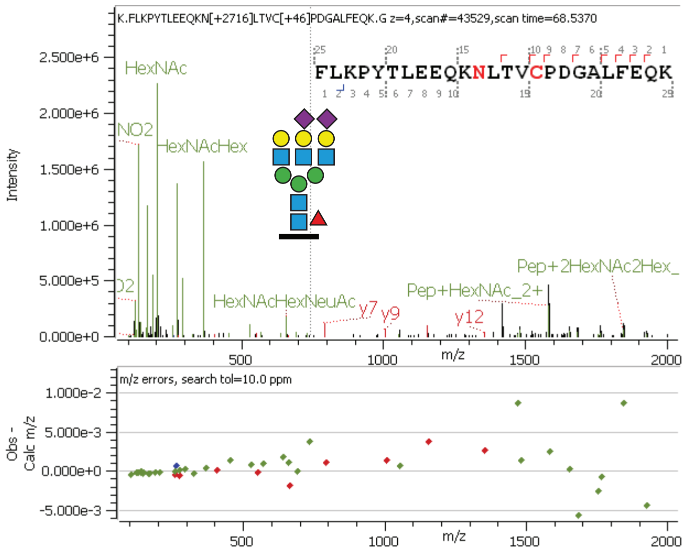

17. CD276, B4GALNT2 WT

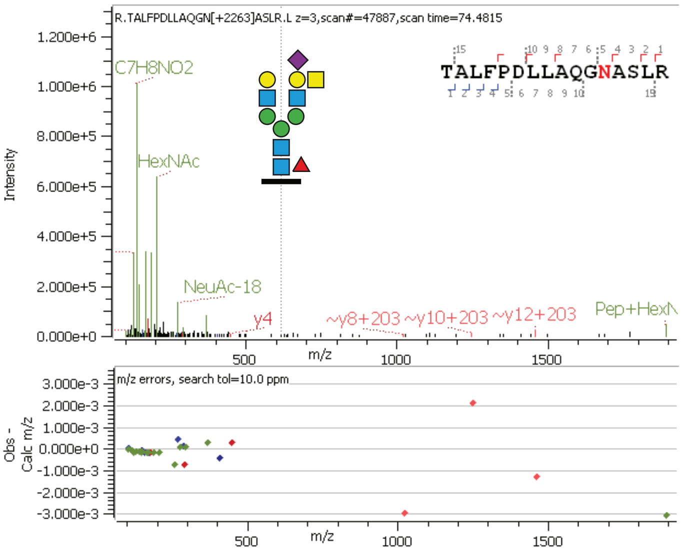

18. CD276, B4GALNT2 WT

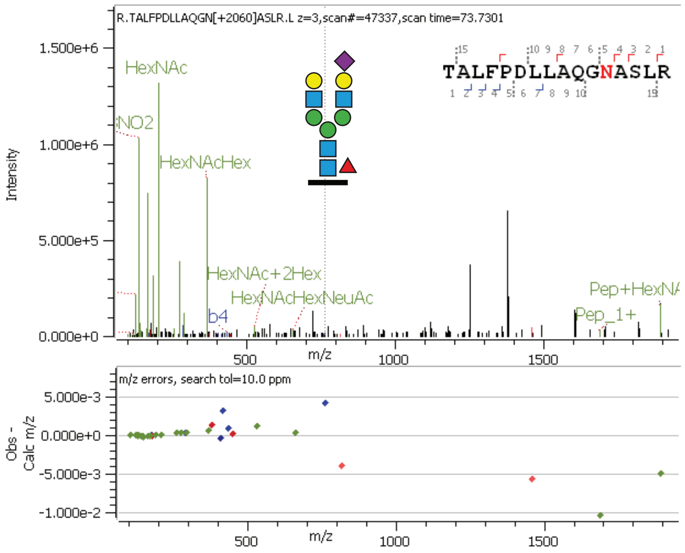

19. PTPRF, B4GALNT2 WT

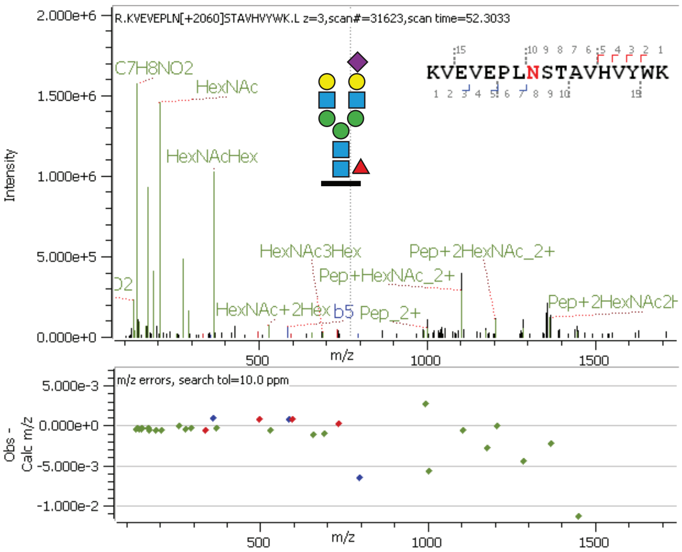

20. TFR1, B4GALNT2 WT

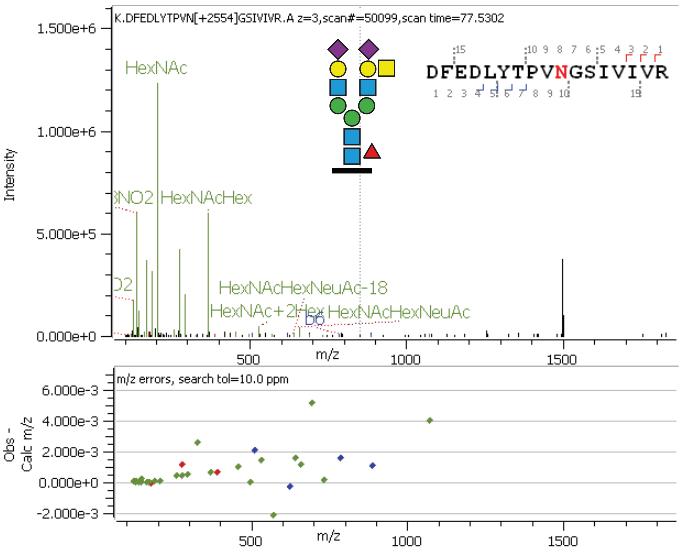

21. TFR1, B4GALNT2 WT

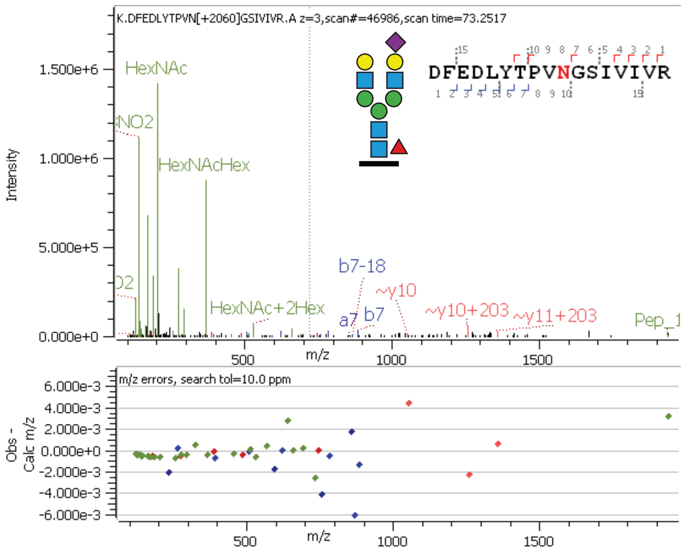

22. TFR1, B4GALNT2 WT

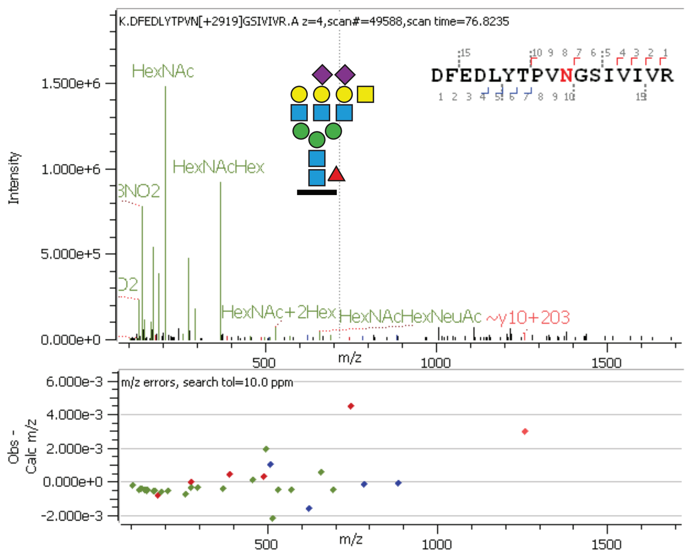

23. TFR1, B4GALNT2 WT

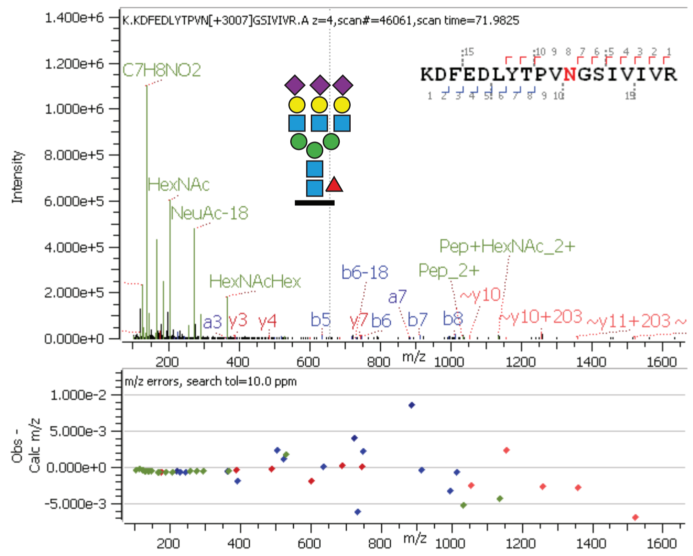

24. 4F2, B4GALNT2 WT

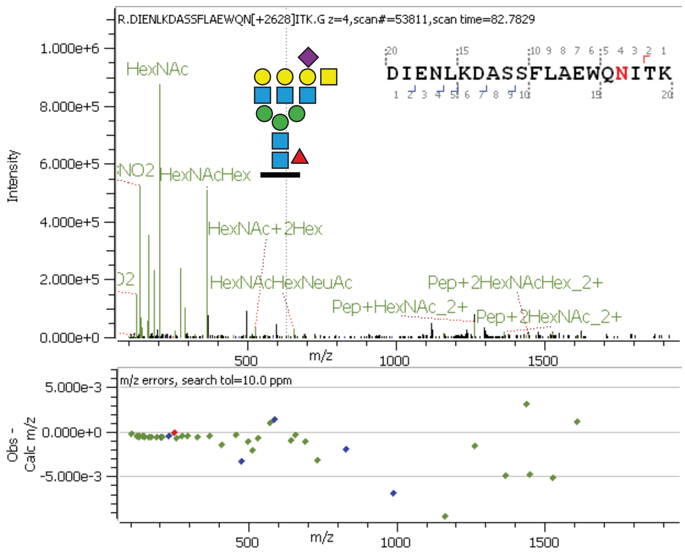

25. LAMP1, B4GALNT2 WT

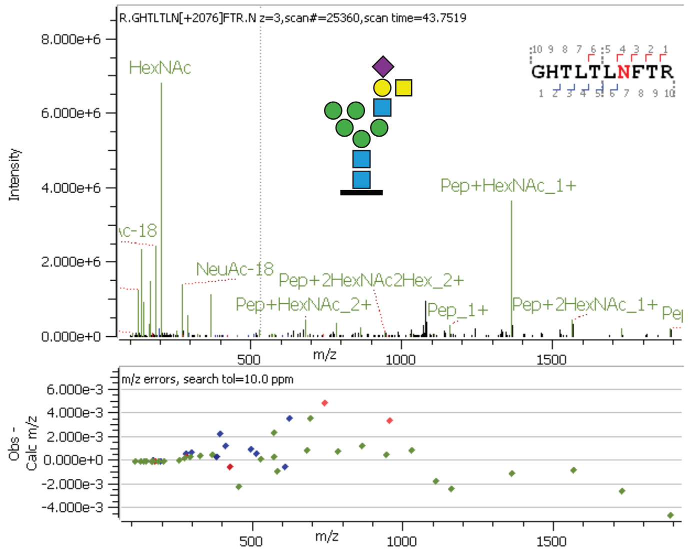

26. LAMP1, B4GALNT2 WT

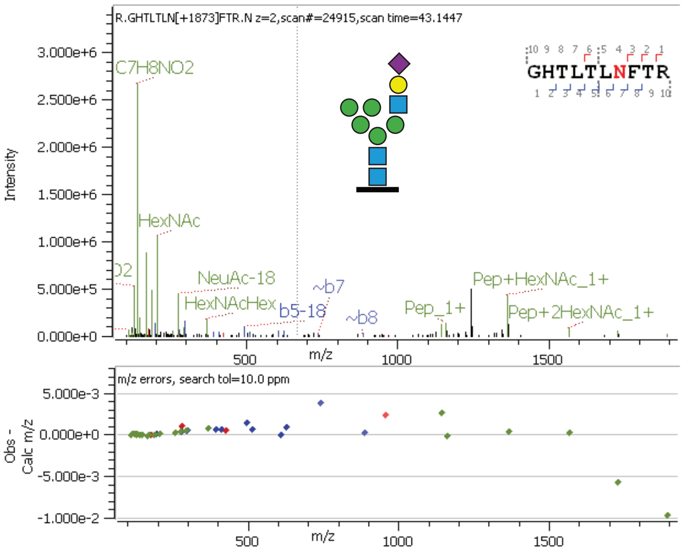

27. TM9S3, rs7224888 (only HCD 20)

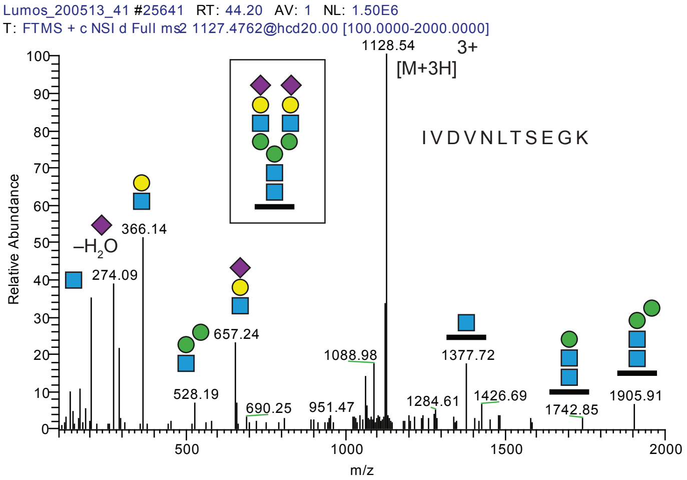

28. AT1B3, rs7224888

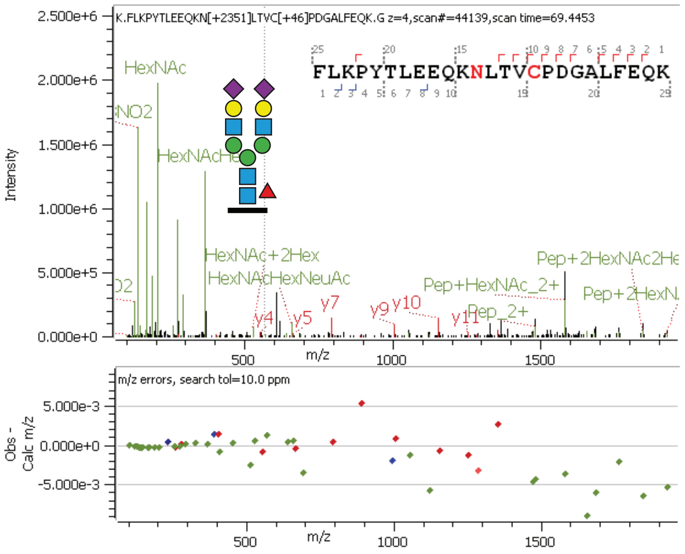

29. AT1B3, rs7224888

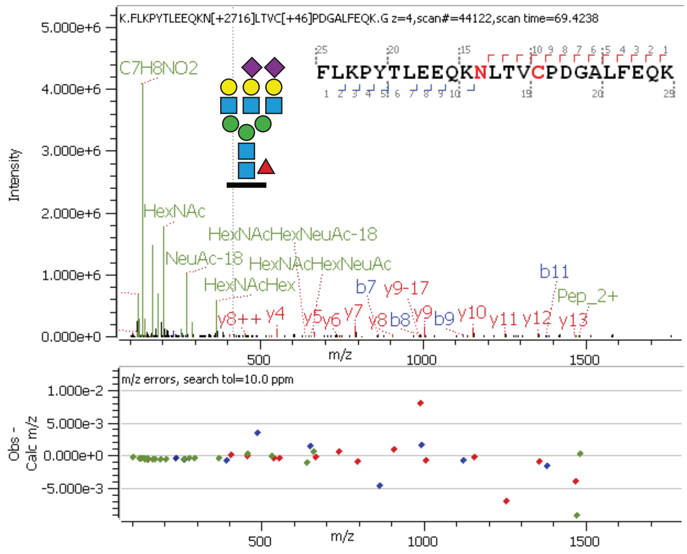

30. CD276, rs7224888

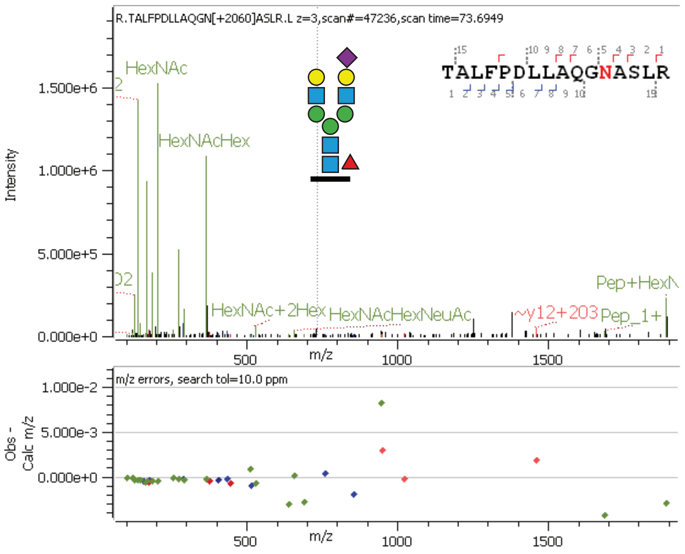

31. PTPRF, rs7224888

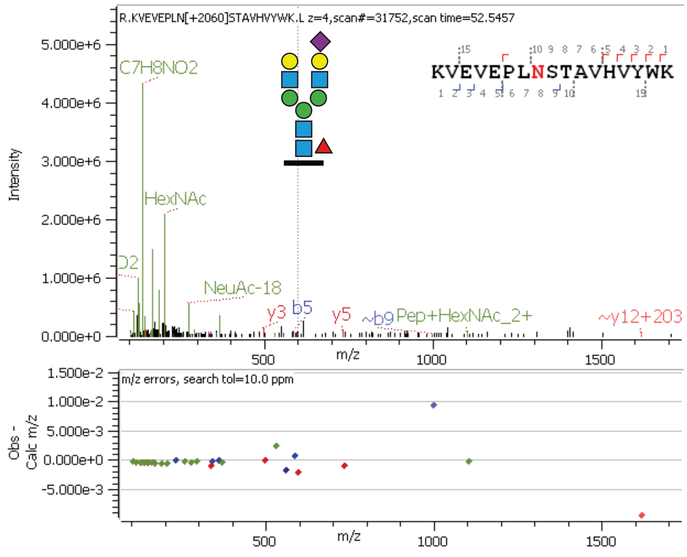

32. TFR1, rs7224888

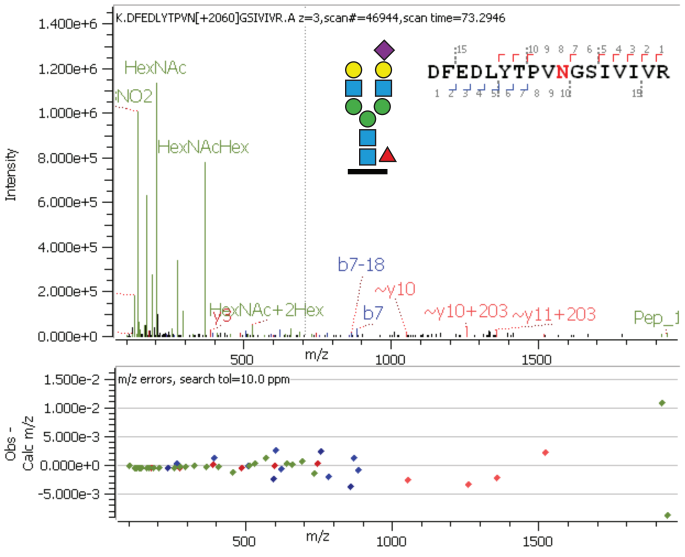

33. TFR1, rs7224888

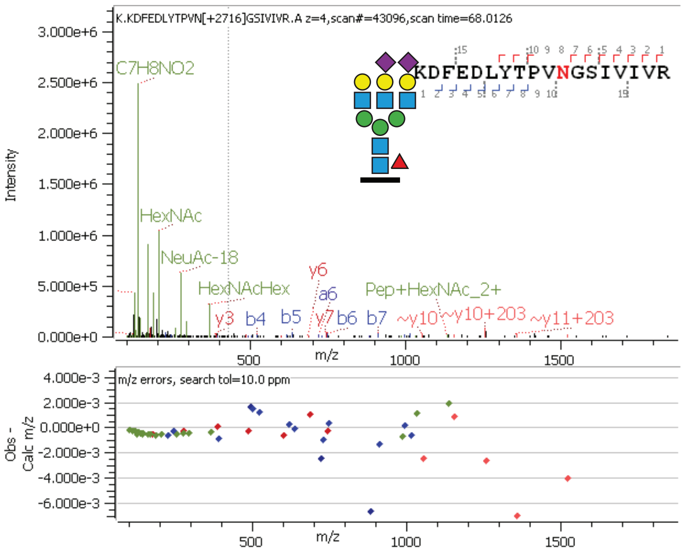

34. 4F2, rs7224888

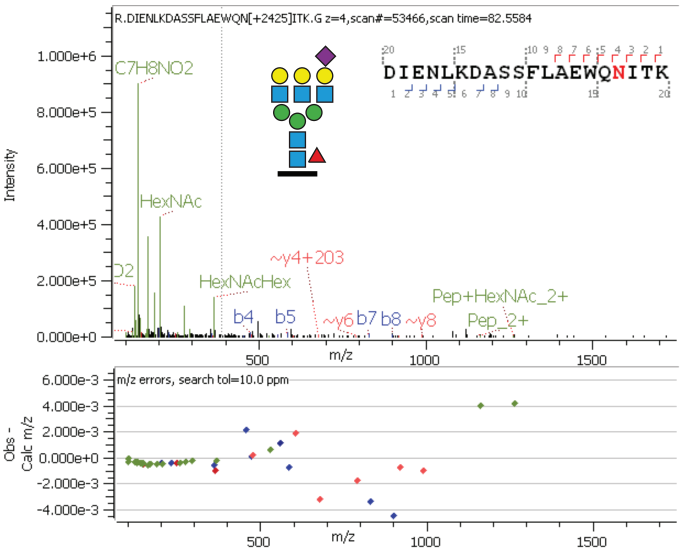

35. CD63, rs7224888

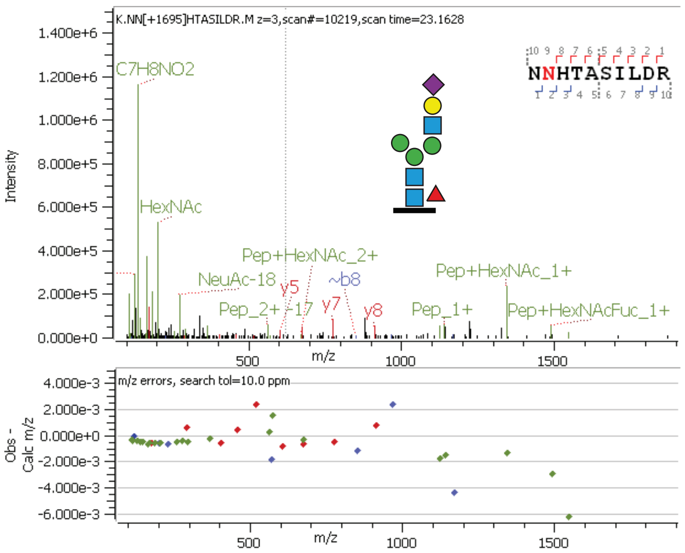

36. LAMP1, rs7224888

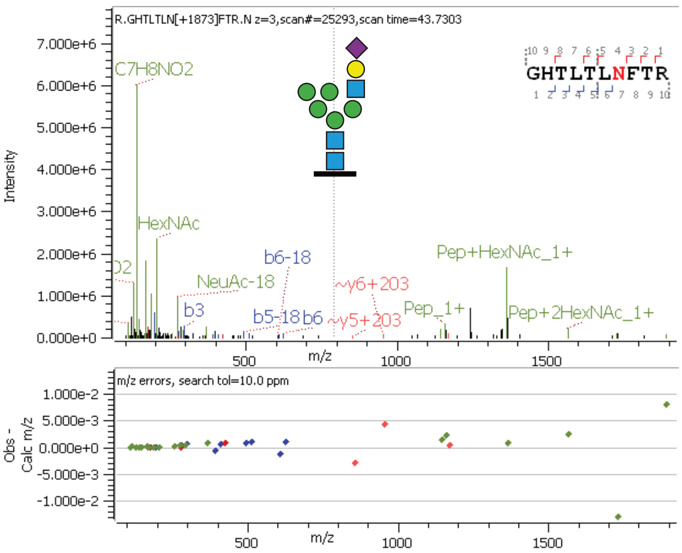

37. TM9S3, rs148441237

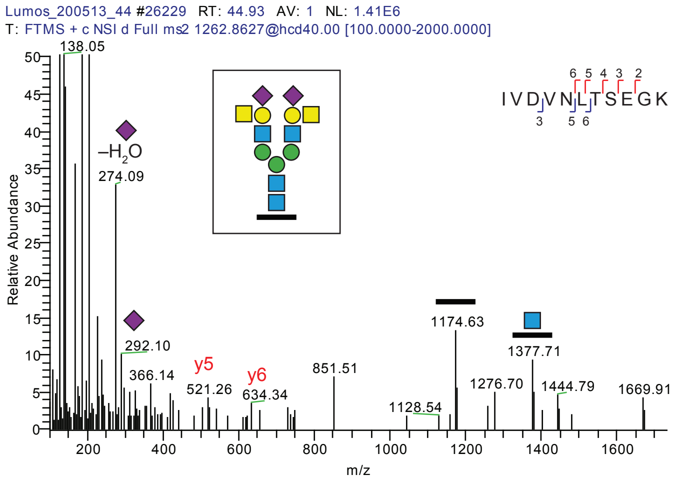

38. AT1B3, rs148441237

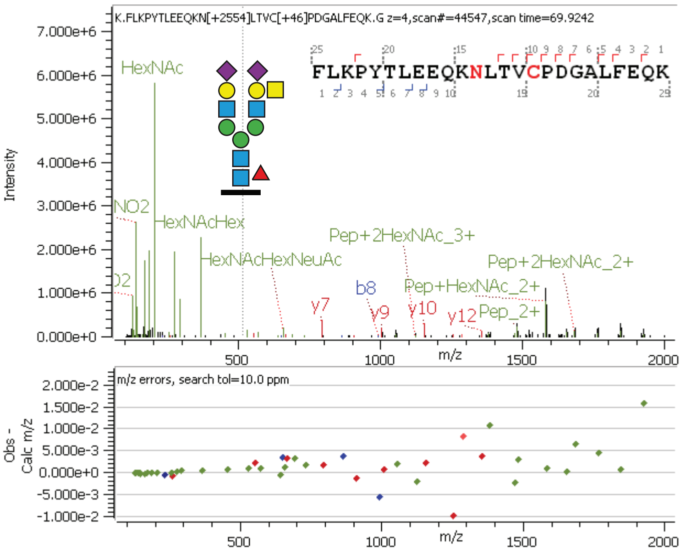

39. AT1B3, rs148441237

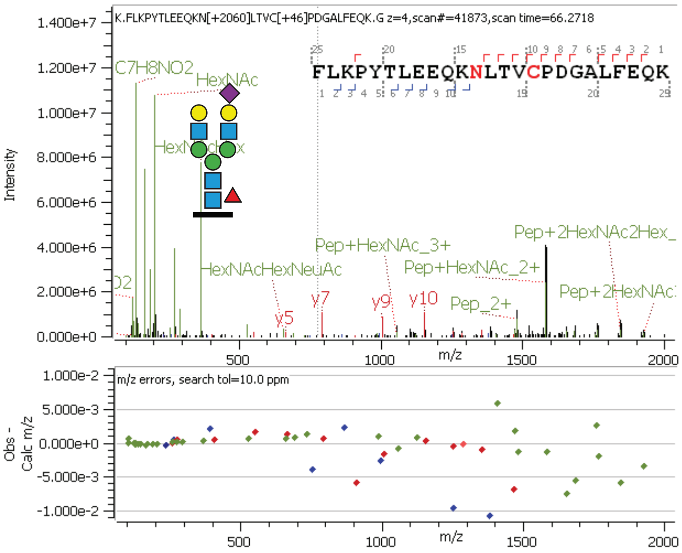

40. AT1B3, rs148441237

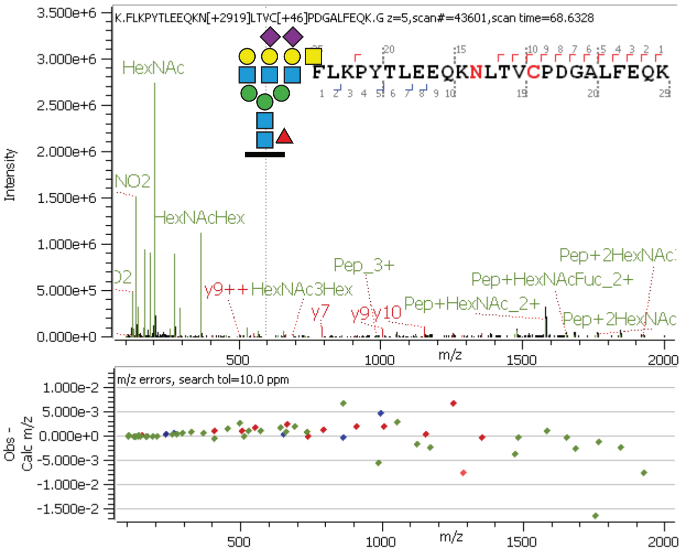

41. AT1B3, rs148441237

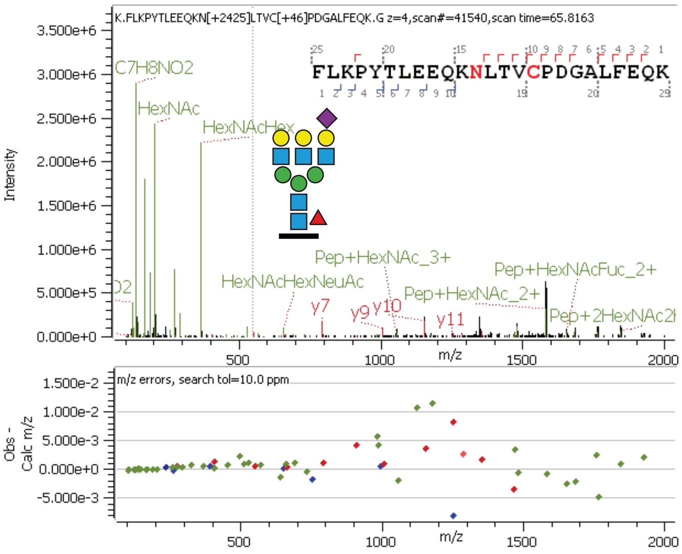

42. CD276, rs148441237

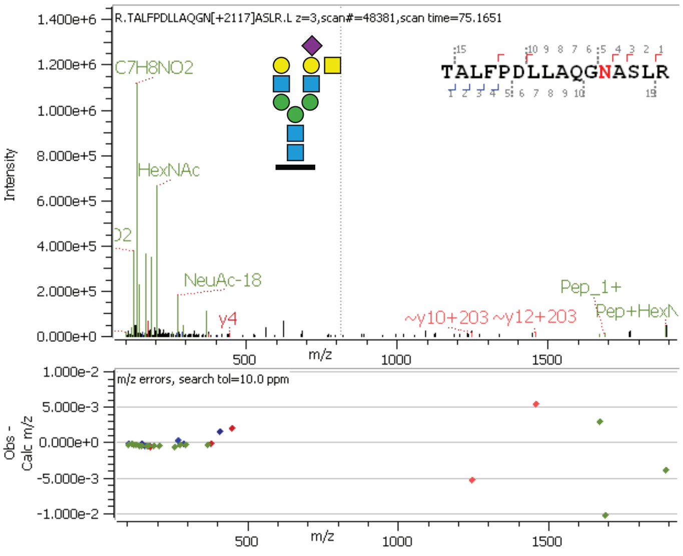

43. CD276, rs148441237

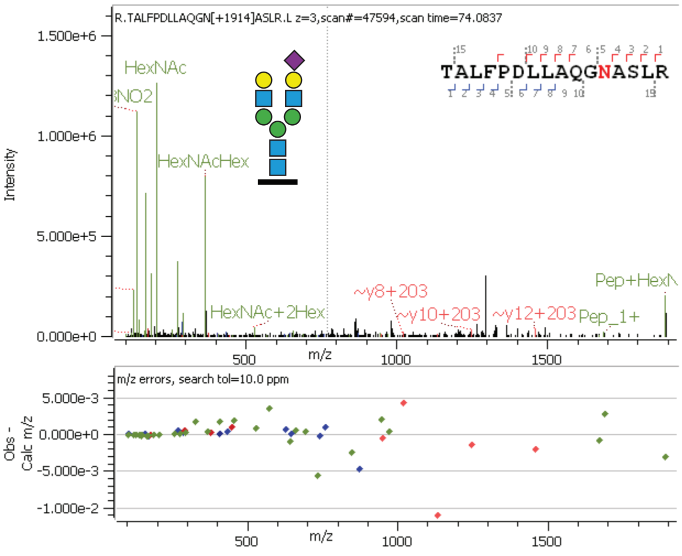

44. PTPRF, rs148441237

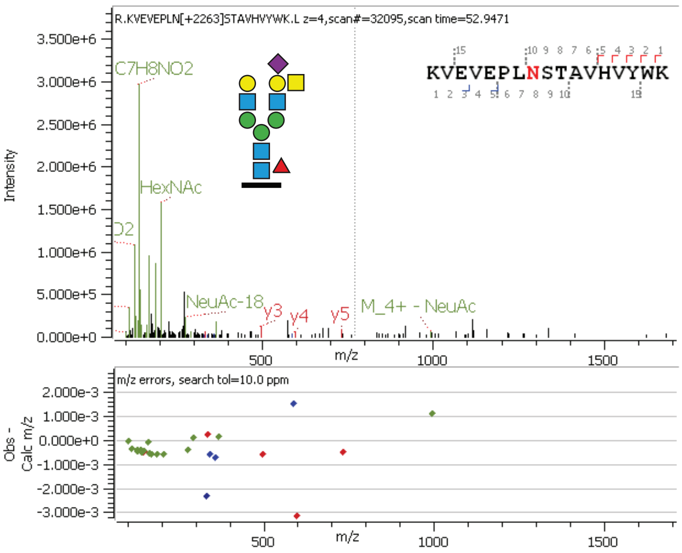

45. PTPRF, rs148441237

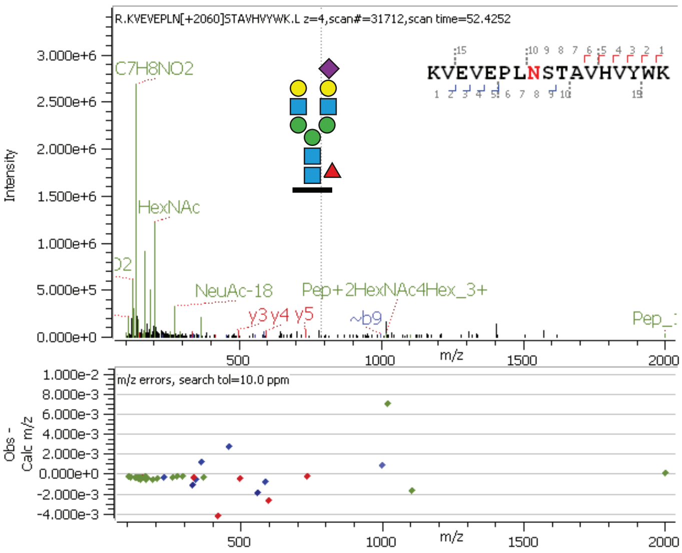

46. TFR1, rs148441237

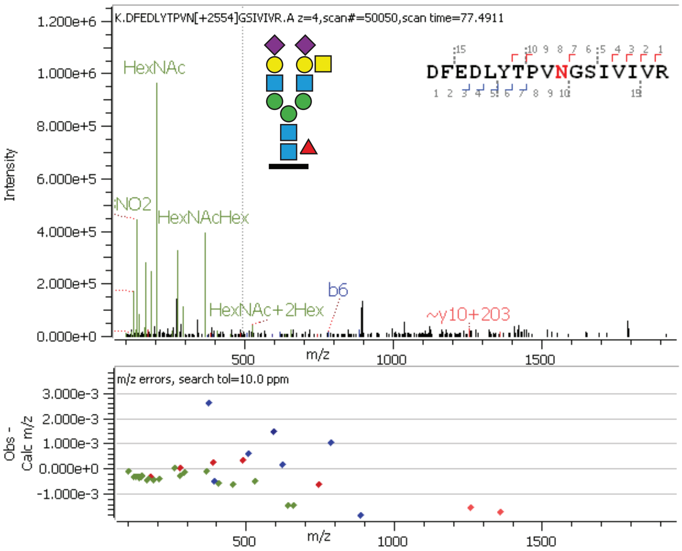

47. TFR1, rs148441237

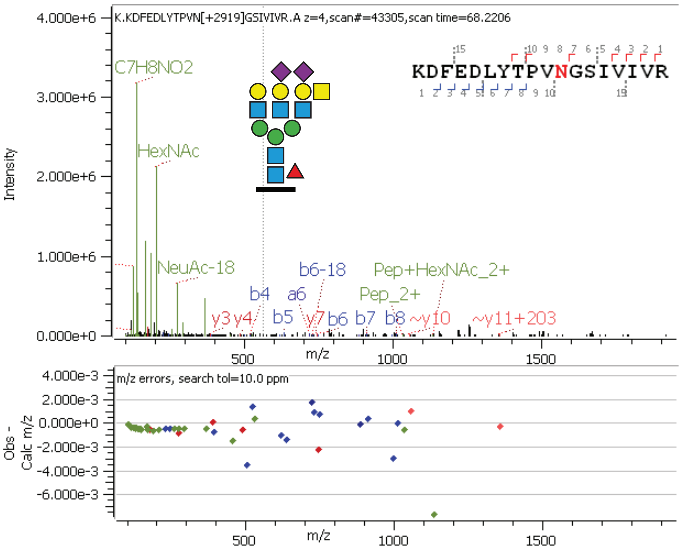

48. TFR1, rs148441237

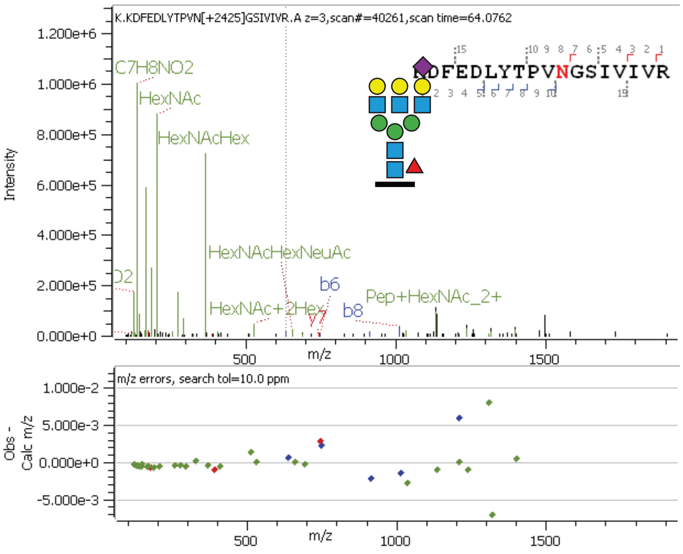

49. CD63, rs148441237

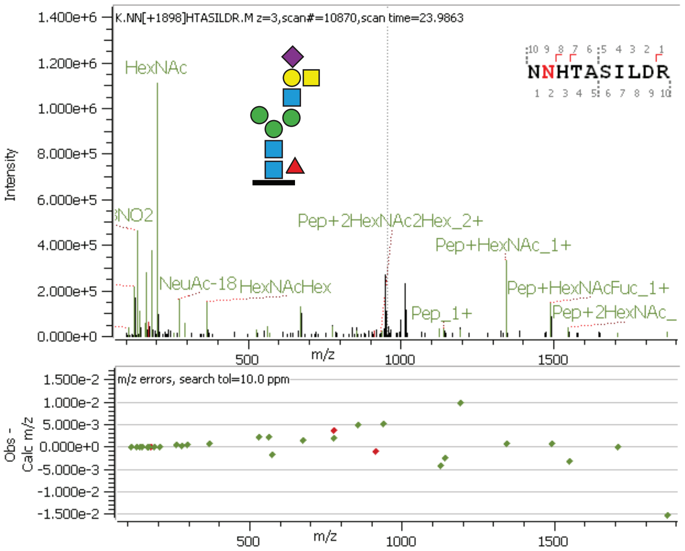

50. CD63, rs148441237

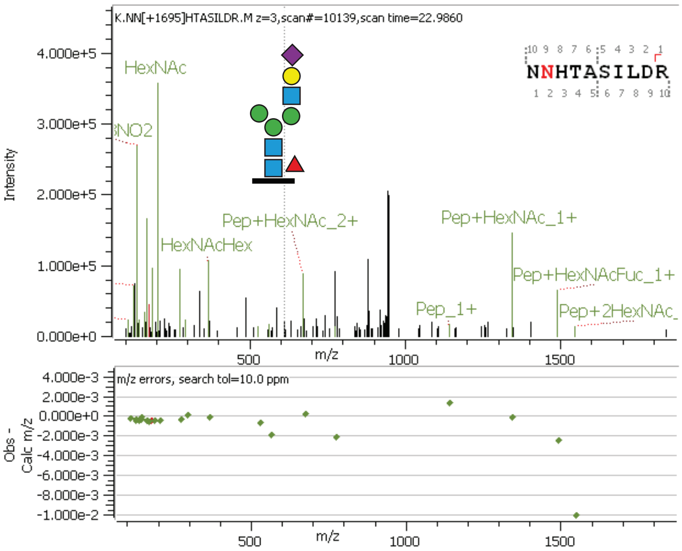

51. LAMP1, rs148441237

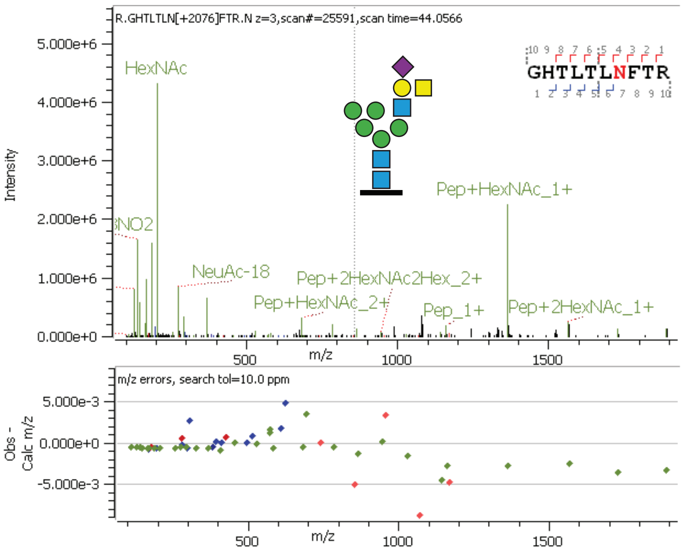

52. LAMP1, rs148441237

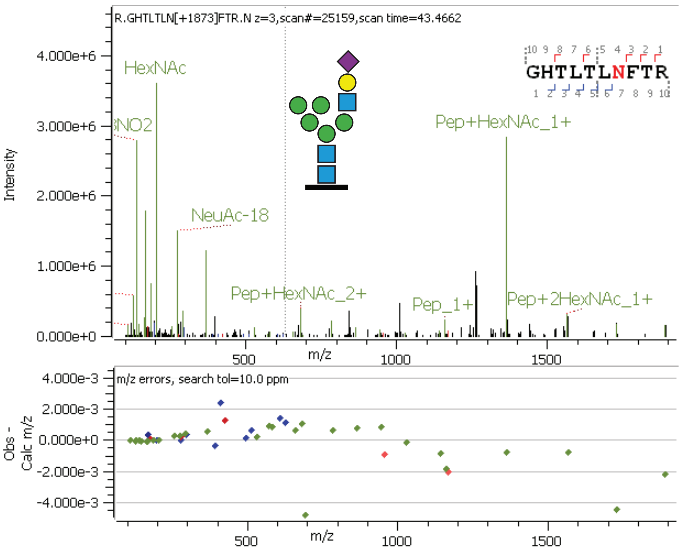

53. TM9S3, rs61743617

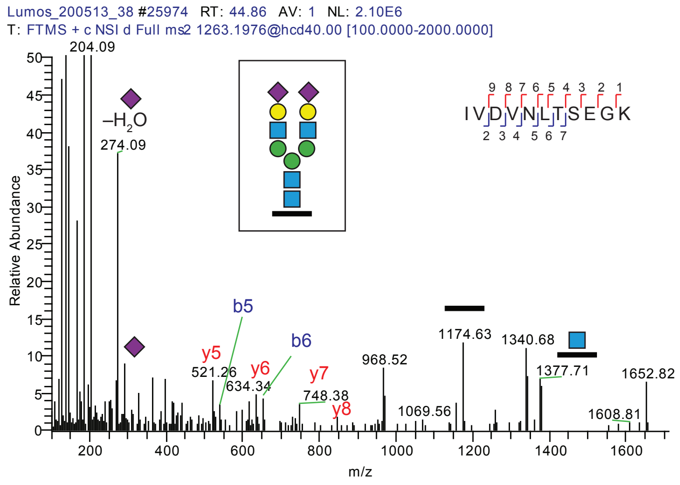

54. AT1B3, rs61743617

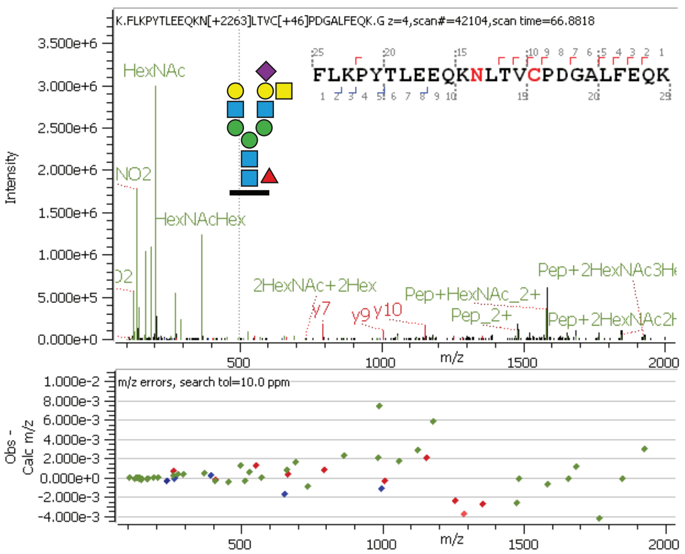

55. AT1B3, rs61743617

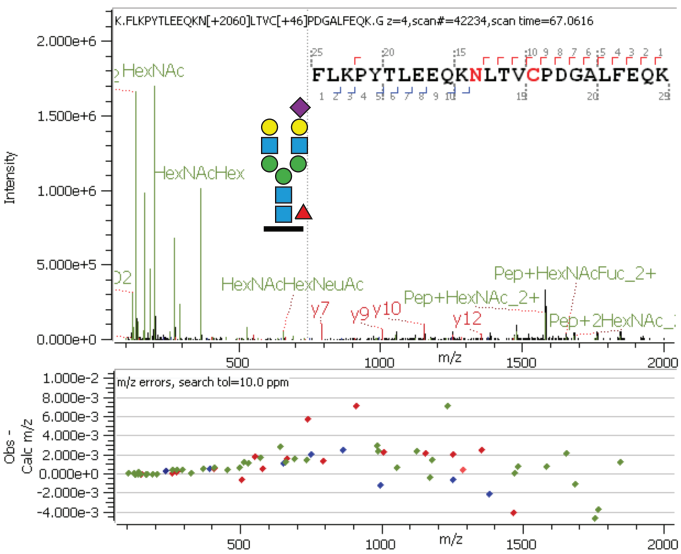

56. AT1B3, rs61743617

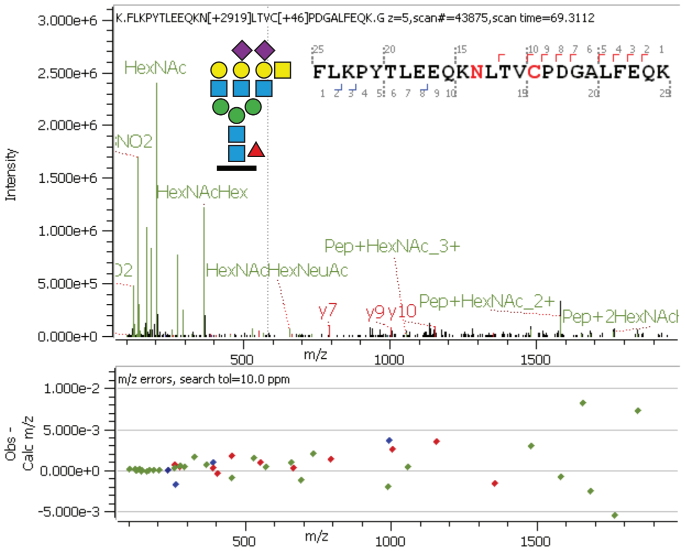

57. AT1B3, rs61743617

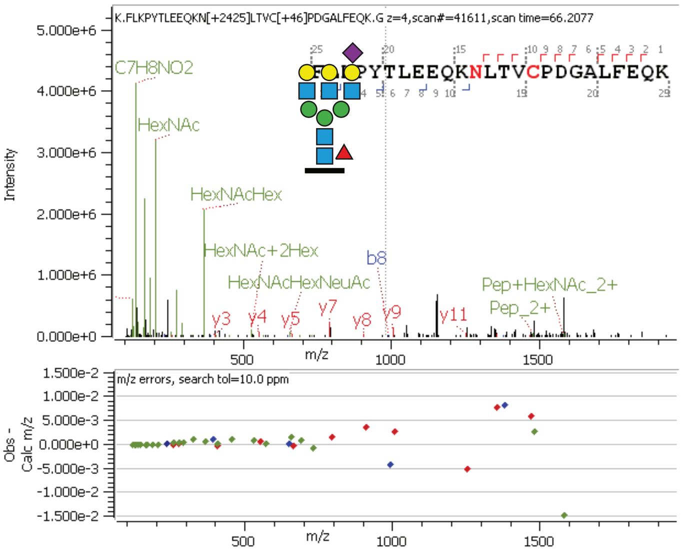

58. CD276, rs61743617

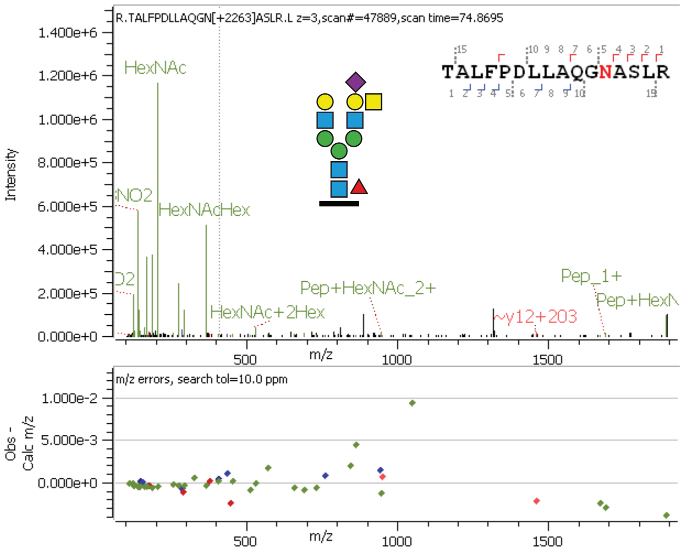

59. CD276, rs61743617

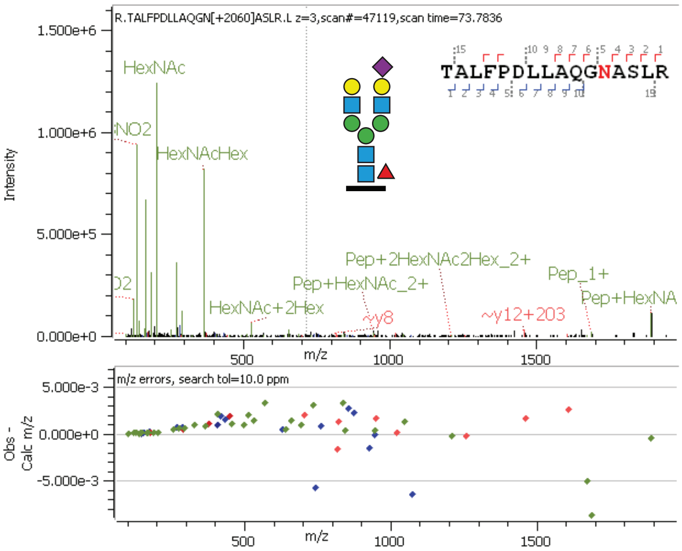

60. PTPRF, rs61743617

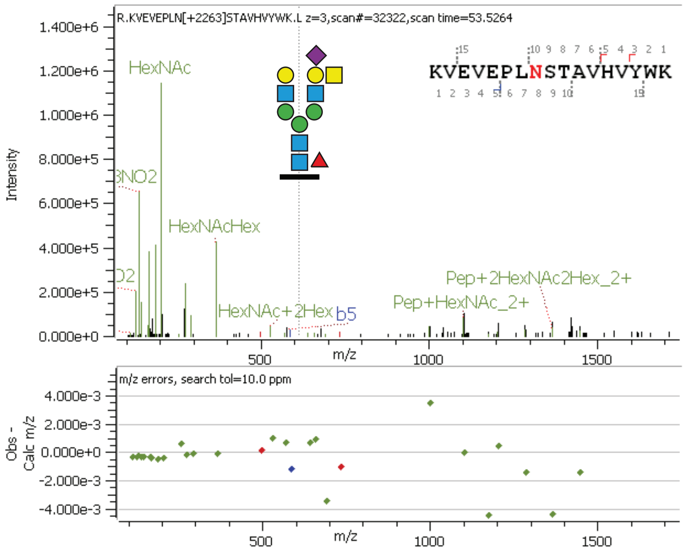

61. PTPRF, rs61743617

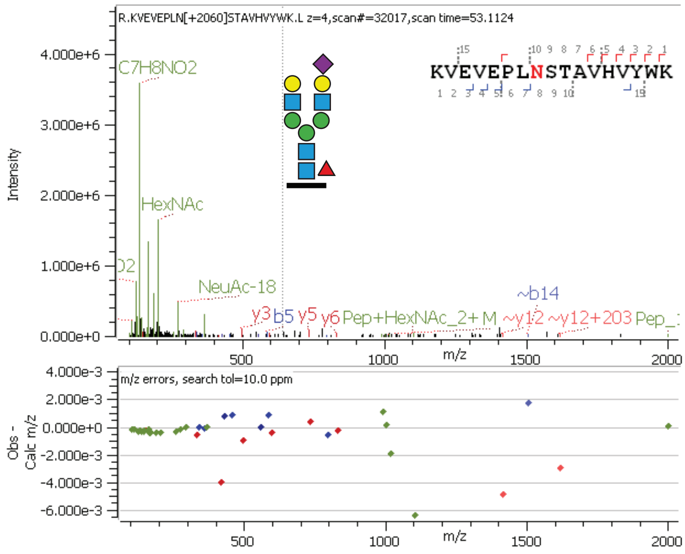

62. TFR1, rs61743617

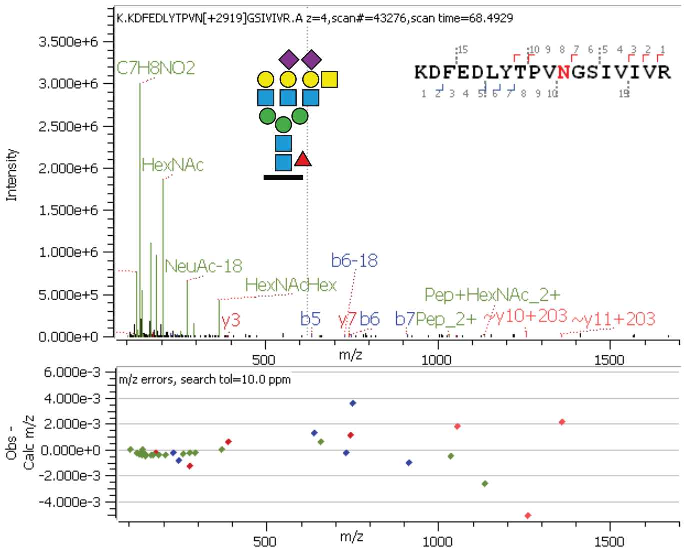

63. TFR1, rs61743617

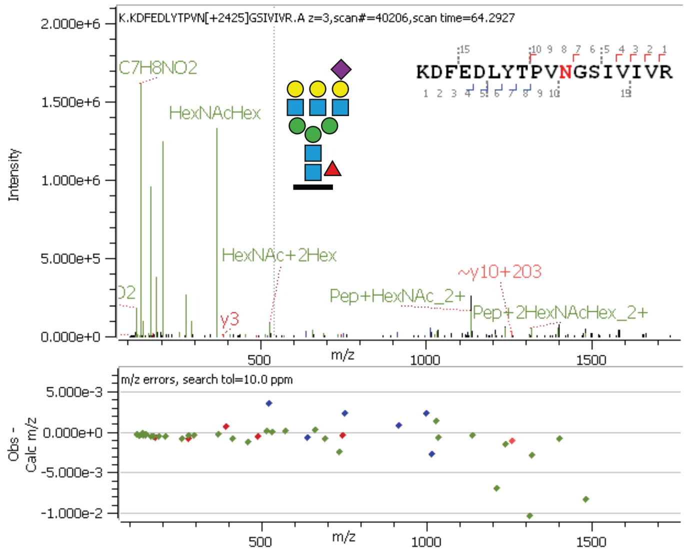

64. CD63, rs61743617

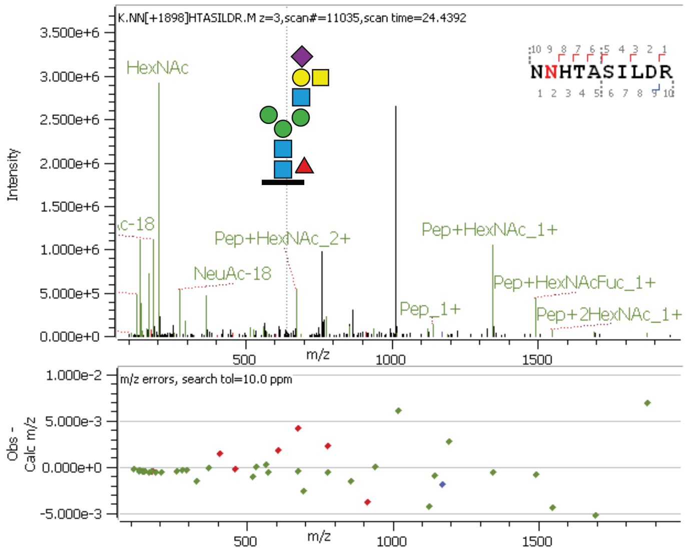

65. LAMP1, rs61743617

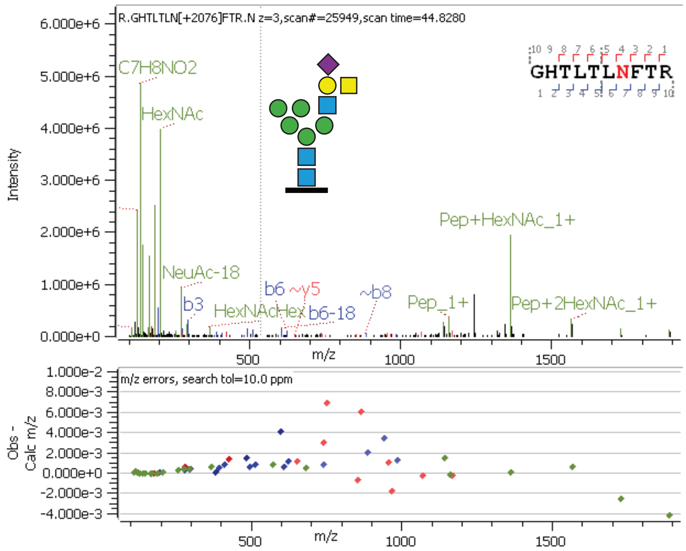

66. LAMP1, rs61743617

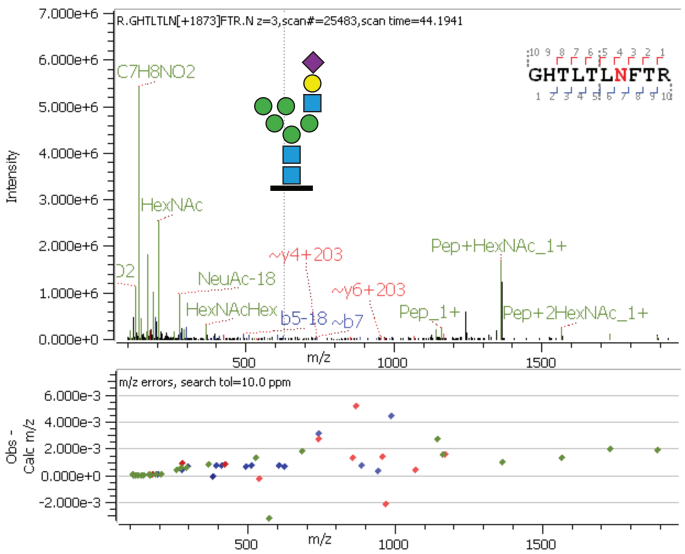

67. GPC4, Mock transfected

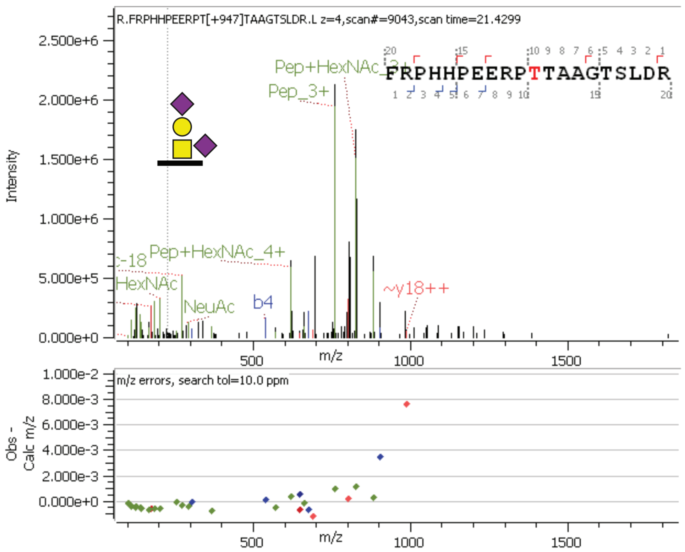

68. FAM3C, Mock transfected

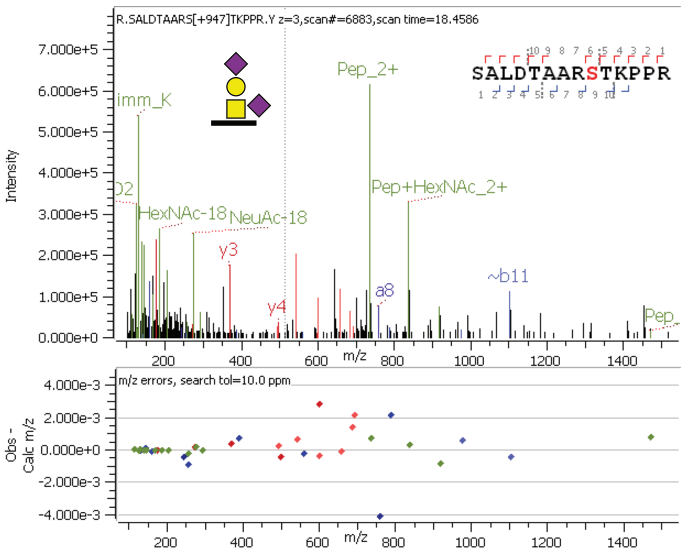

69. TFR1, Mock transfected

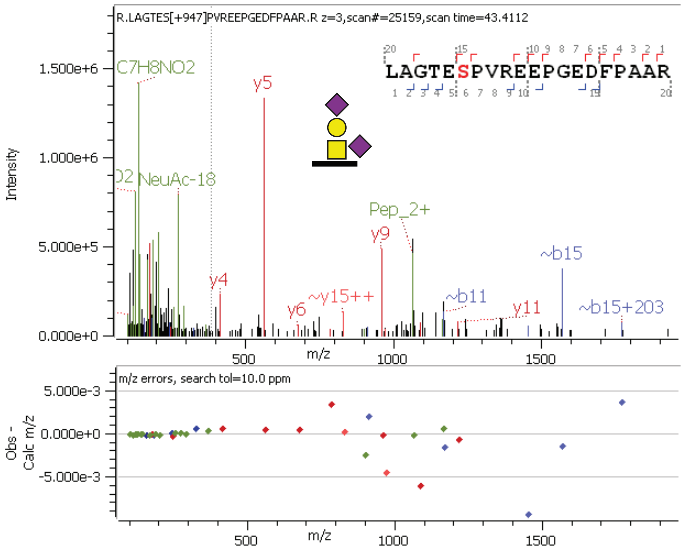

70. GPC4, B4GALNT2 WT

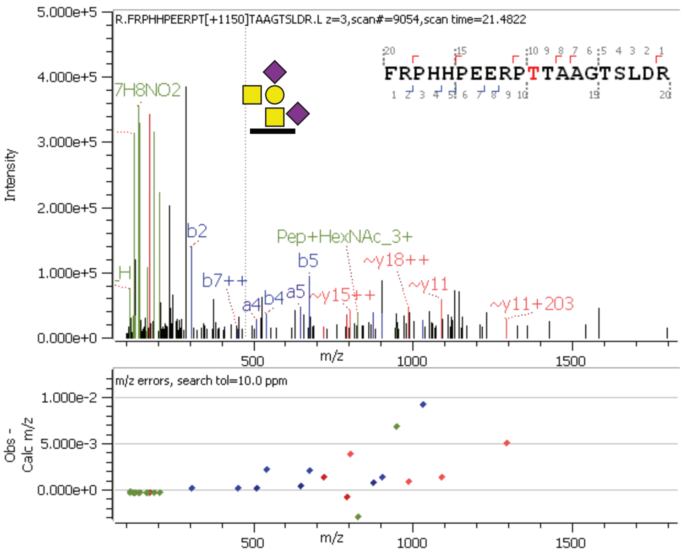

71. FAM3C, B4GALNT2 WT

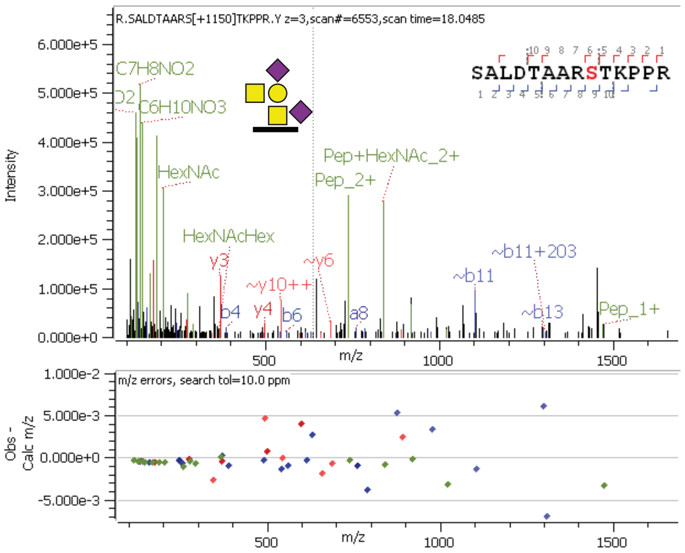

72. FAM3C, B4GALNT2 WT

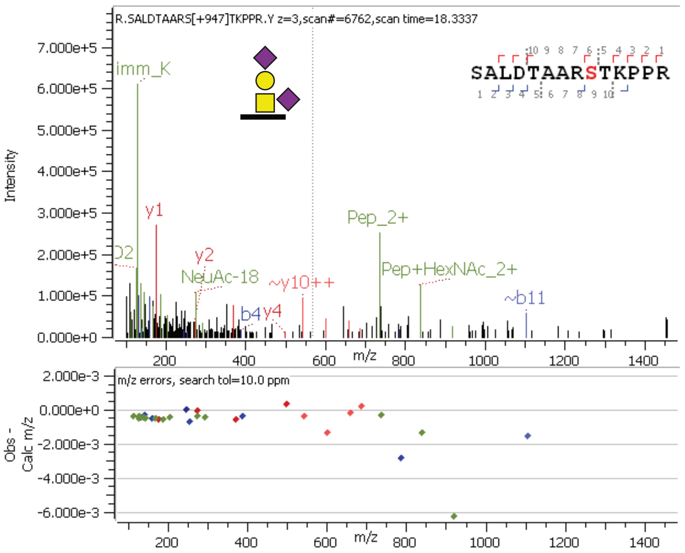

73. TFR1, B4GALNT2 WT

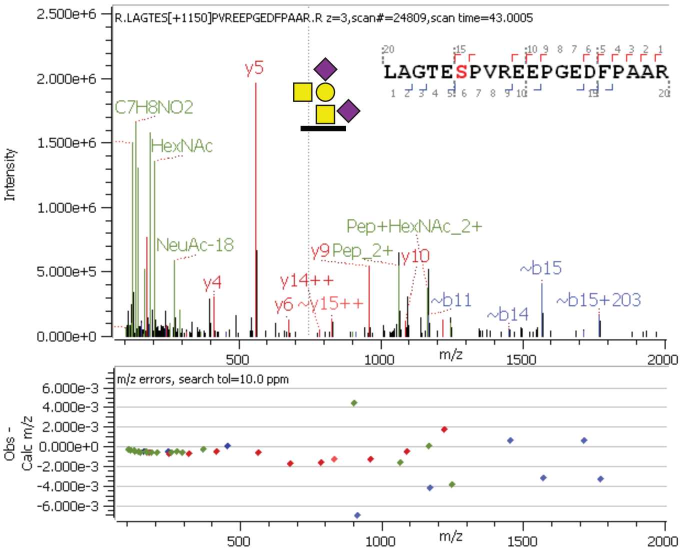

74. GPC4, rs7224888

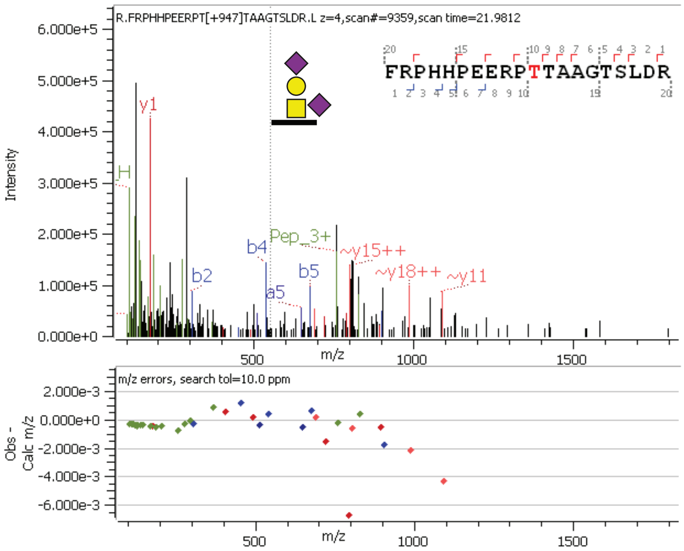

75. FAM3C, rs7224888

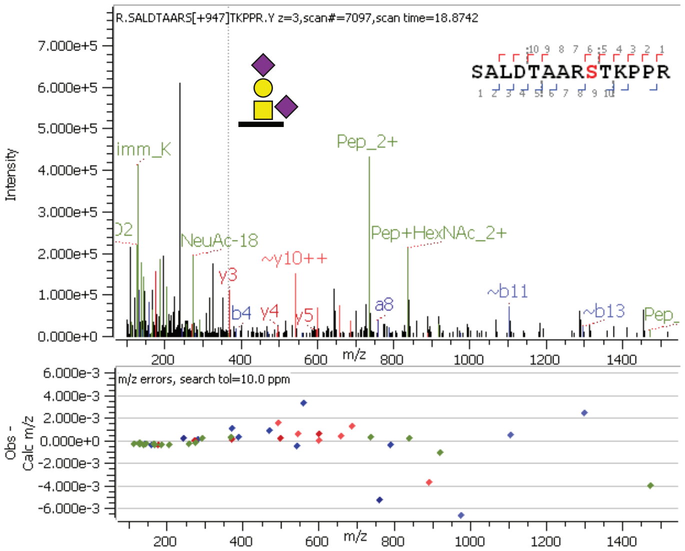

76. TFR1, rs7224888

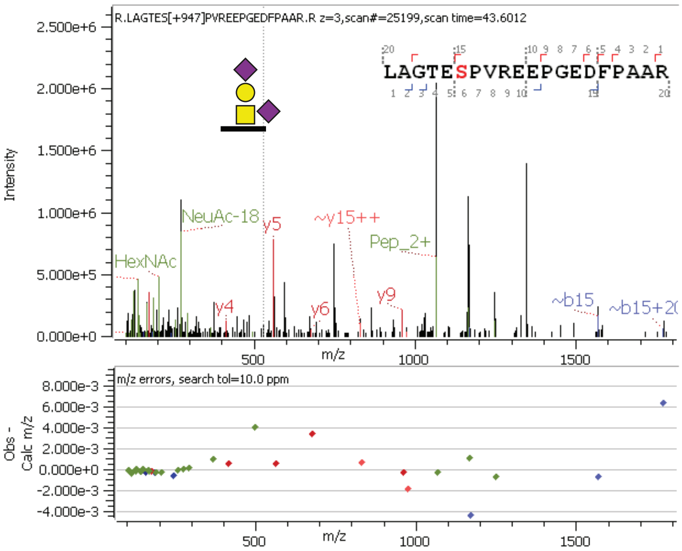

77. GPC4, rs148441237

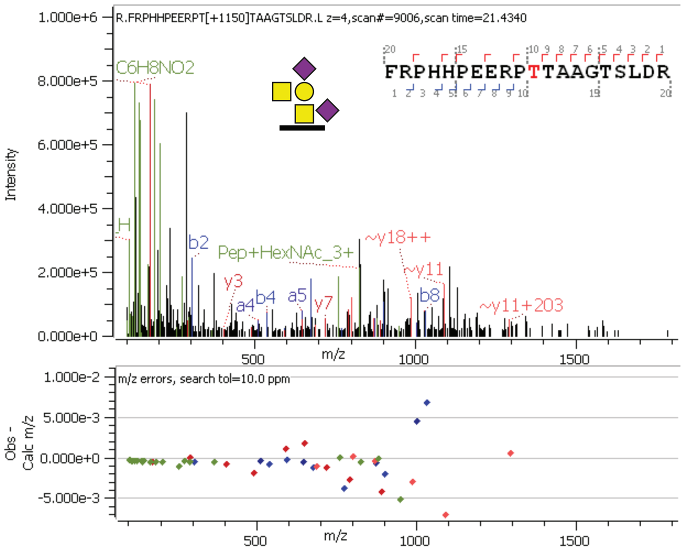

78. FAM3C, rs148441237

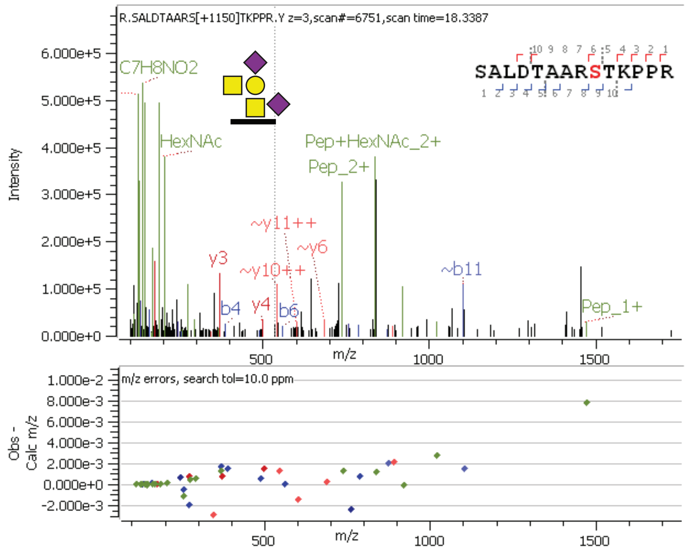

79. TFR1, rs148441237

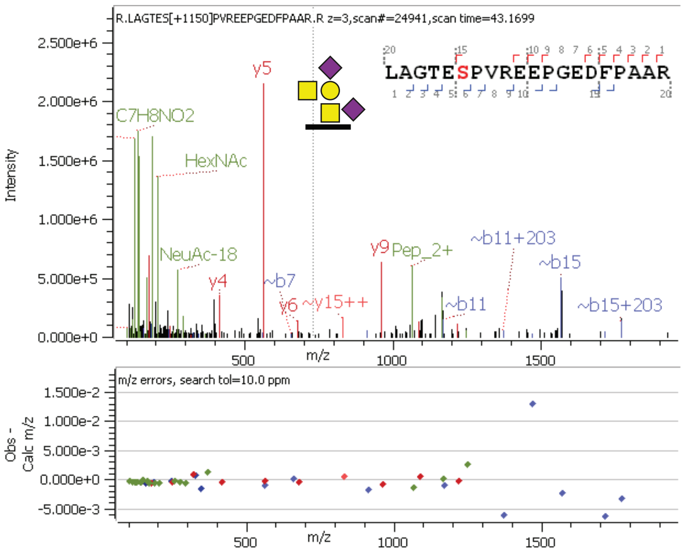

80. GPC4, rs61743617

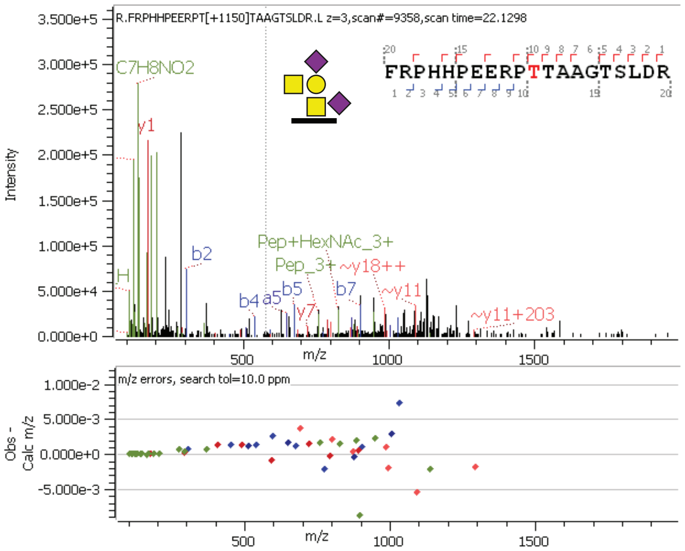

81. GPC4, rs61743617

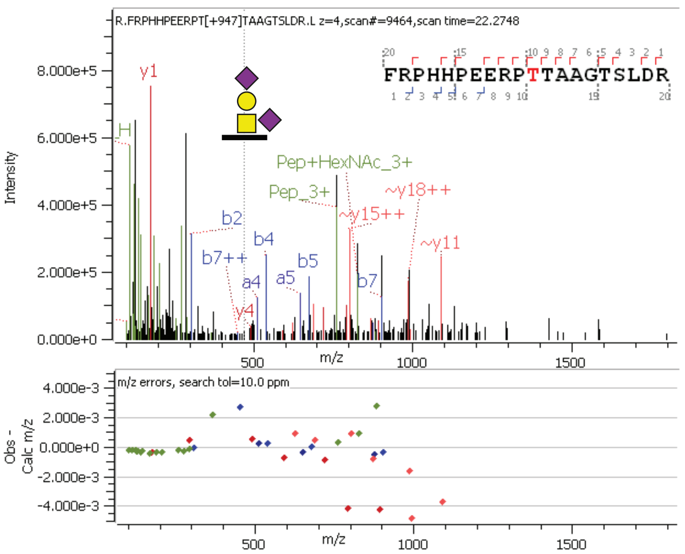

82. FAM3C, rs61743617

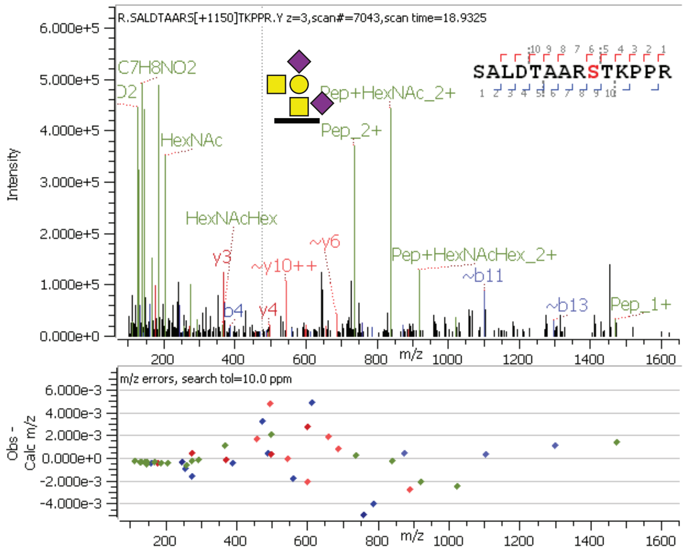

83. TFR1, rs61743617

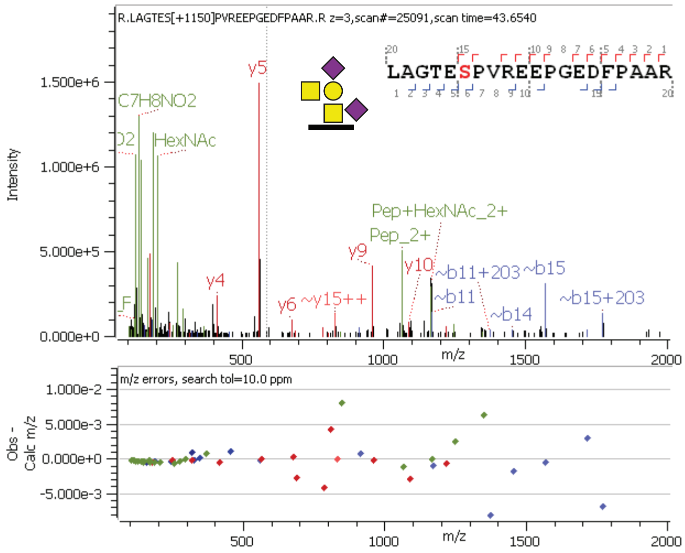

84. TFR1, rs61743617

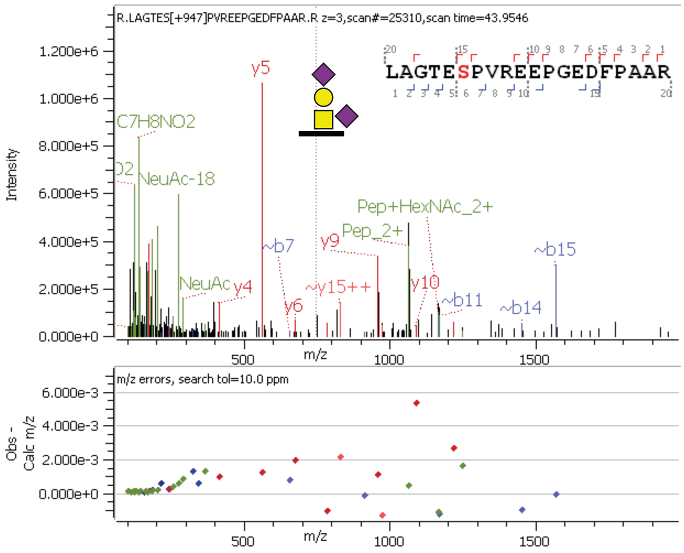

Spectrum collection 2, **Part 2.** Byonic annotated MS^2 spectra of glycopeptides from erythrocytes. Information regarding MS files, scan numbers, precuror masses and charges are provided in Supplementary Table S4 and S5.

1. BAND3, Cad-a

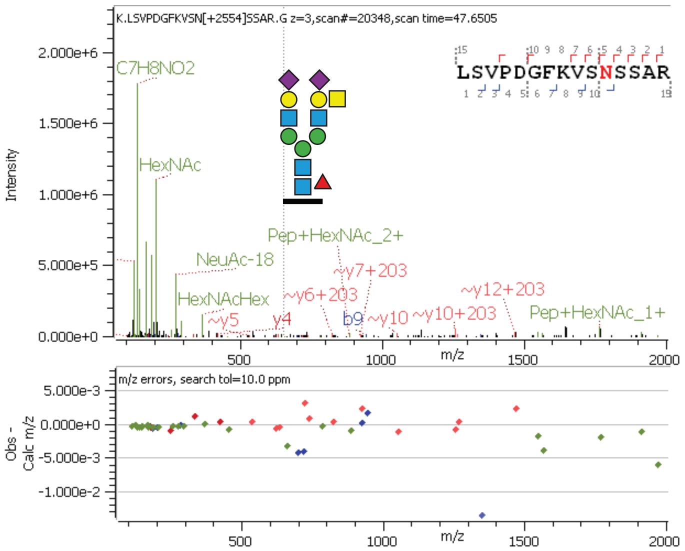

2. BAND3, Cad-a

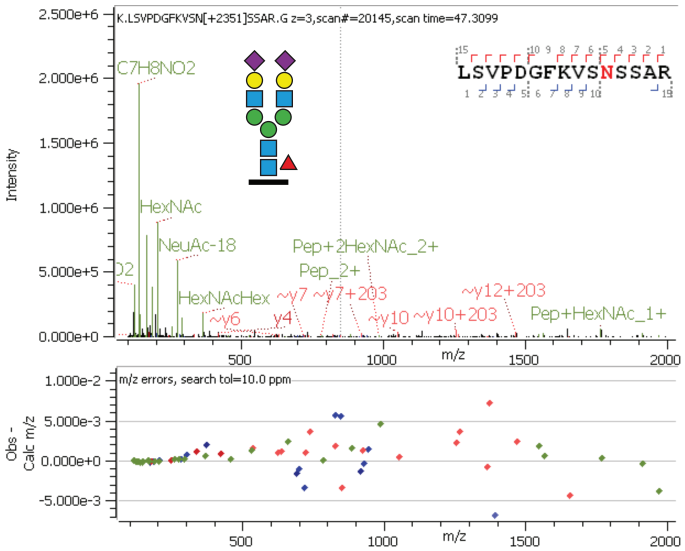

3. BAND3, Control

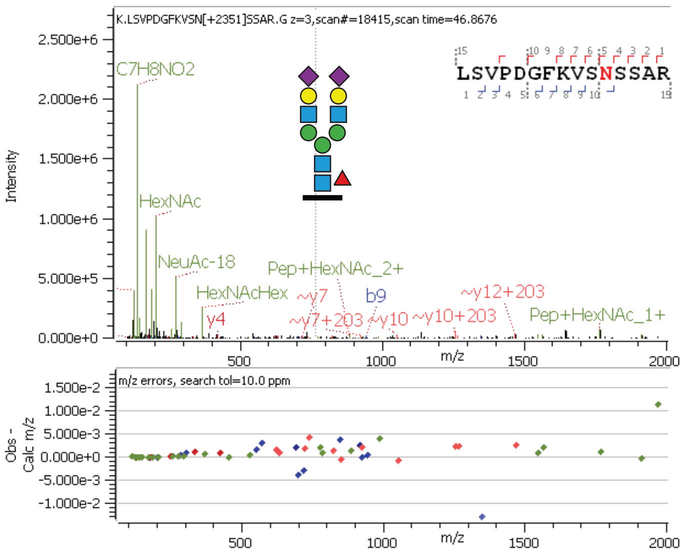

4. BAND3, Cad-a (chymotrypsin)

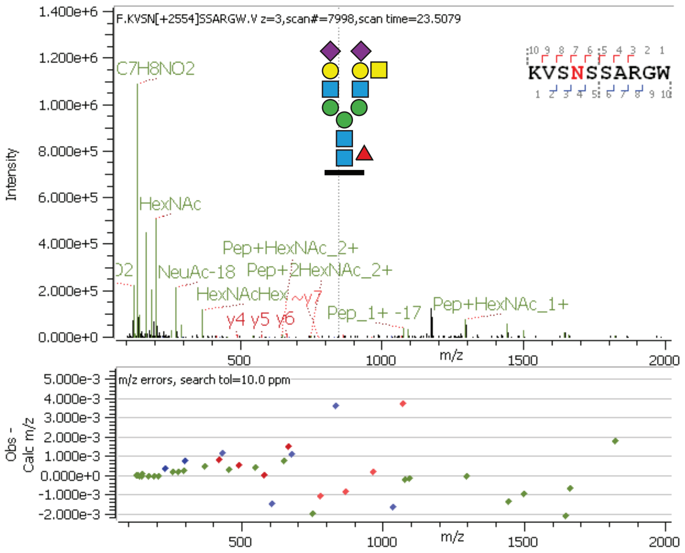

5. BAND3, Cad-a (chymotrypsin)

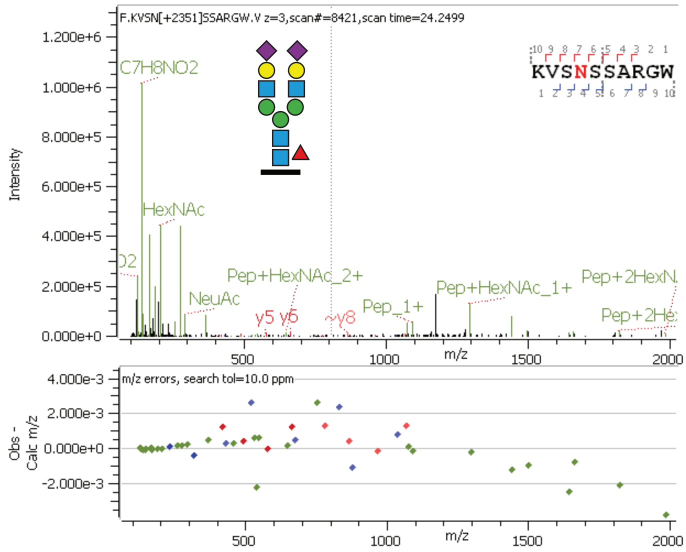

6. GLPA, Cad-a

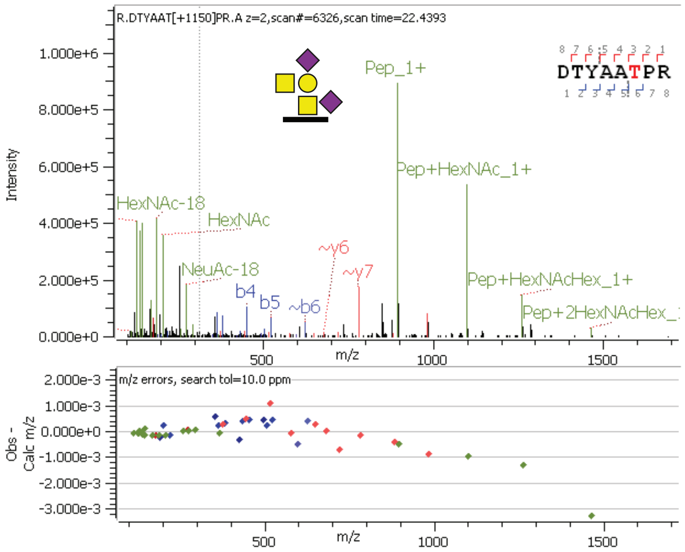

7. GLPA, Cad-a

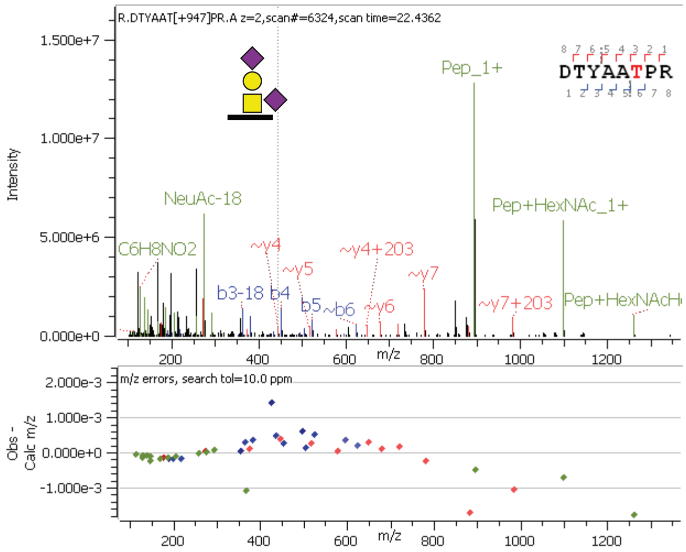

8. GLPA, Cad-a

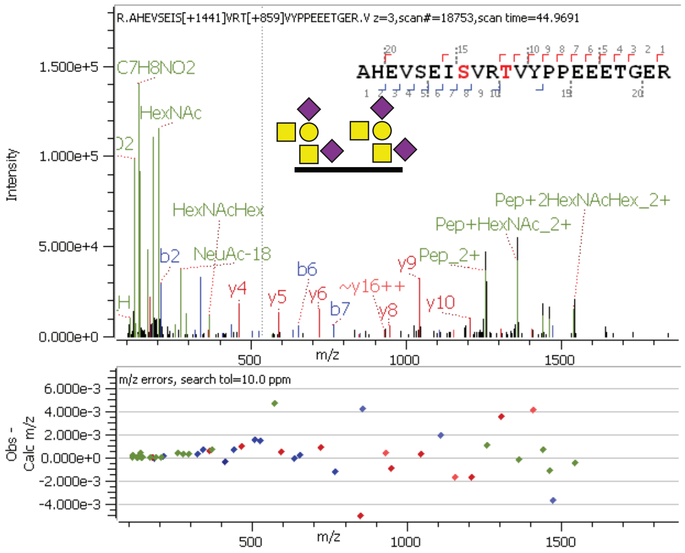

9. GLPA, Cad-a

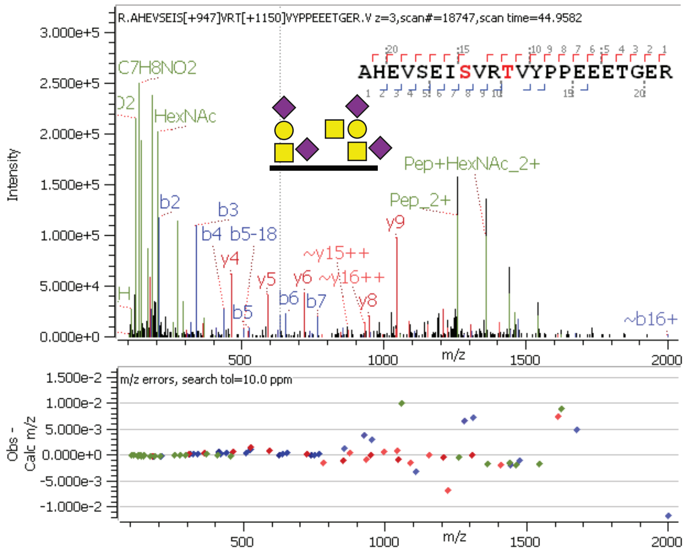

10. GLPA, Cad-a

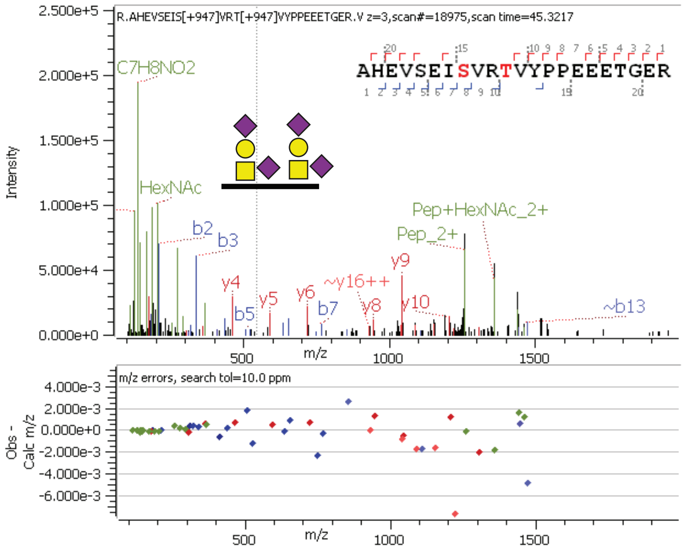

11. S29A1, Cad-a

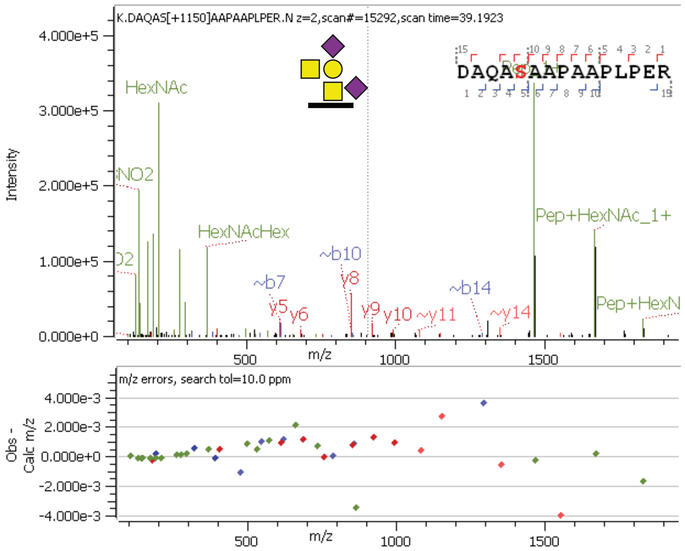

12. S29A1, Cad-a

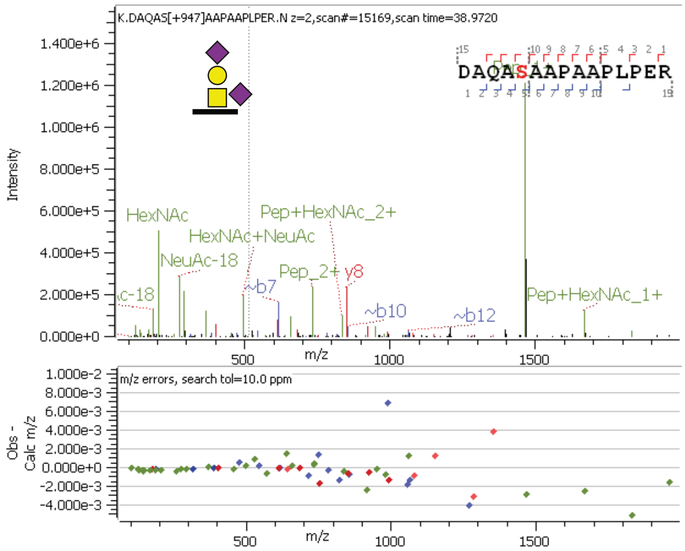

13. GLPA, Cad-b

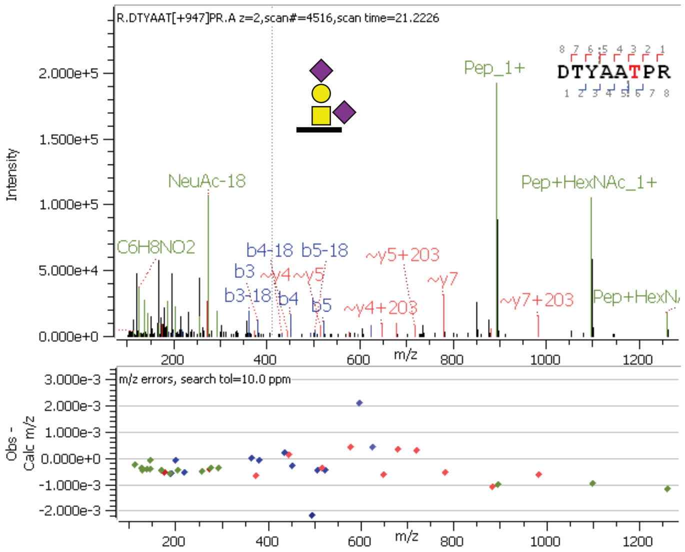

14. GLPA, Cad-b

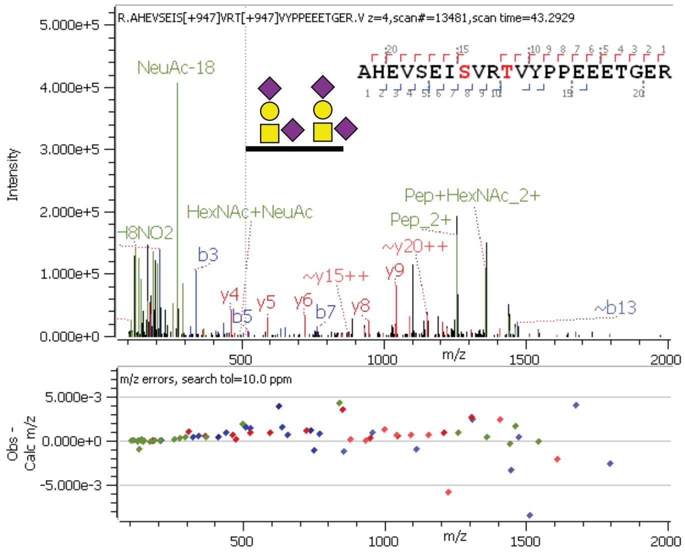

15. S29A1, Cad-b

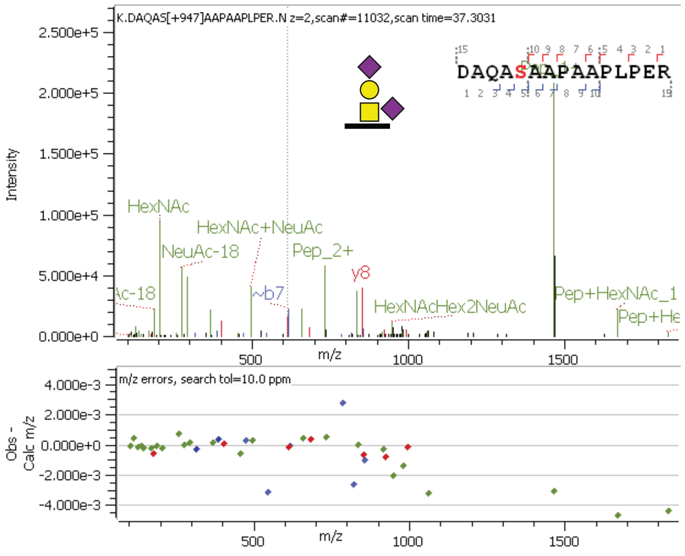

16. GLPA, Cad-b (pronase)

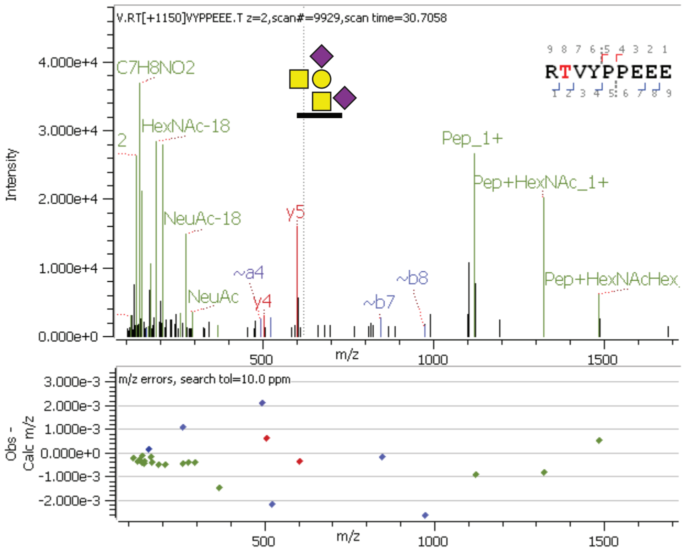

17. GLPA, Cad-b (pronase)

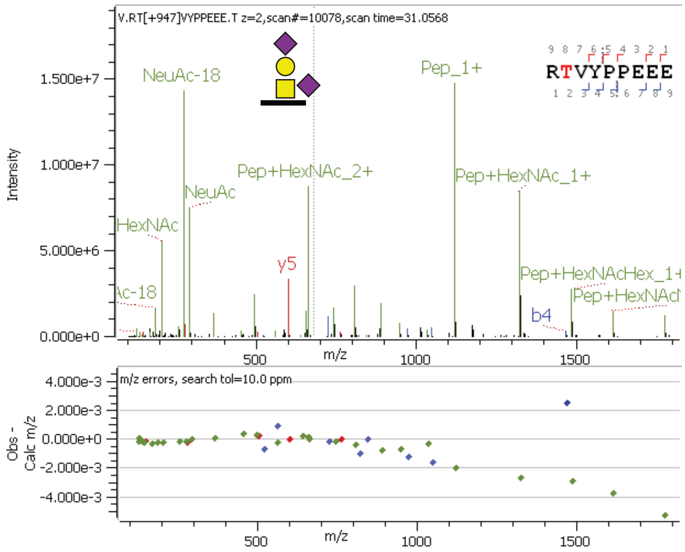

18. GLPA, Control (pronase)

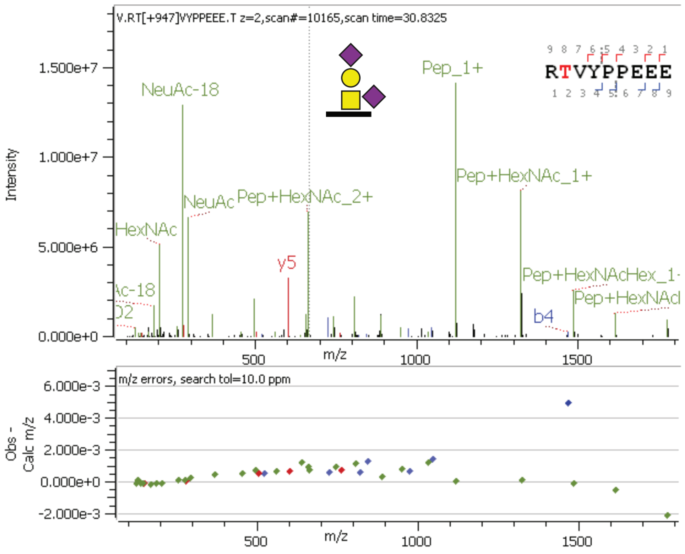

19. GLPA, Control

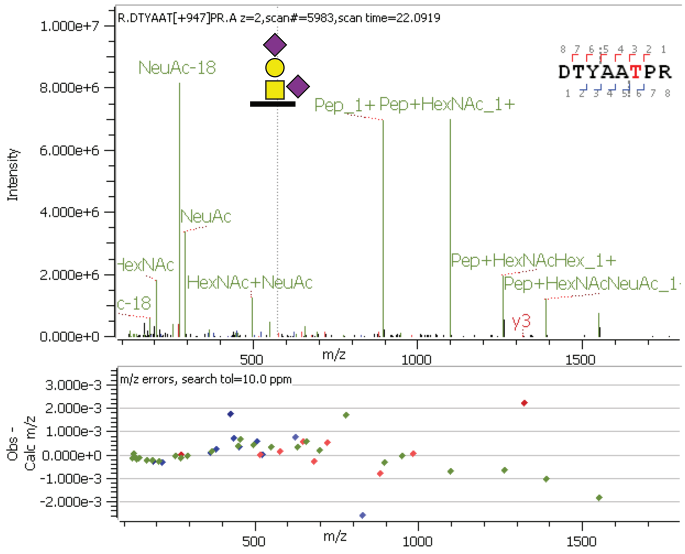

20. GLPA, Control

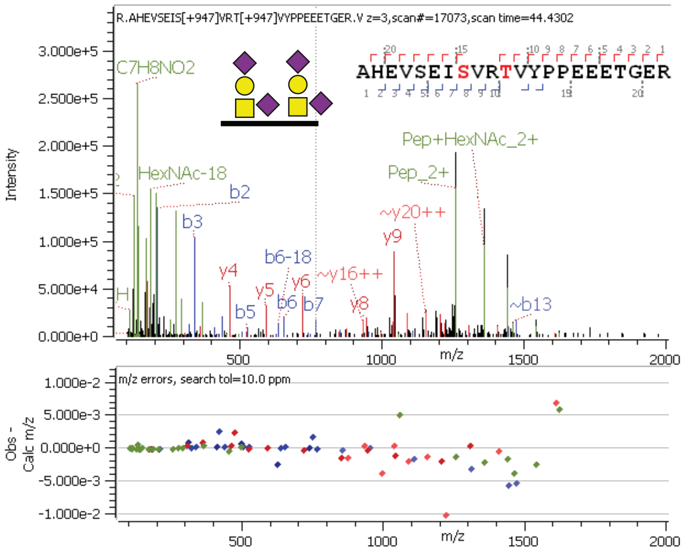

21. S29A1, Control

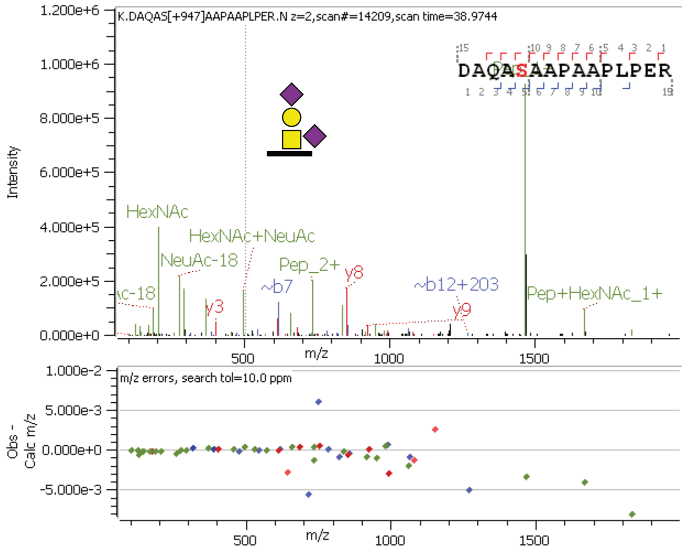

Supplement: Supplementary file 1 [file ijms-23-03936-s001.zip › Supplementary Materials - Spectra Collections 1 and 2_proof.pdf]
